# Genome-wide identification, structural analysis and new insights into late embryogenesis abundant (*LEA*) gene family formation pattern in *Brassica napus*

**DOI:** 10.1038/srep24265

**Published:** 2016-04-13

**Authors:** Yu Liang, Ziyi Xiong, Jianxiao Zheng, Dongyang Xu, Zeyang Zhu, Jun Xiang, Jianping Gan, Nadia Raboanatahiry, Yongtai Yin, Maoteng Li

**Affiliations:** 1 Department of Biotechnology, College of Life Science and Technology, Huazhong University of Science and Technology, Wuhan, 430074 China; 2 Hubei Collaborative Innovation Center for the Characteristic Resources Exploitation of Dabie Mountains, Huanggang Normal University, Huanggang 438000, China

## Abstract

Late embryogenesis abundant (LEA) proteins are a diverse and large group of polypeptides that play important roles in desiccation and freezing tolerance in plants. The LEA family has been systematically characterized in some plants but not *Brassica napus*. In this study, 108 *BnLEA* genes were identified in the *B. napus* genome and classified into eight families based on their conserved domains. Protein sequence alignments revealed an abundance of alanine, lysine and glutamic acid residues in BnLEA proteins. The *BnLEA* gene structure has few introns (<3), and they are distributed unevenly across all 19 chromosomes in *B. napus*, occurring as gene clusters in chromosomes A9, C2, C4 and C5. More than two-thirds of the *BnLEA* genes are associated with segmental duplication. Synteny analysis revealed that most *LEA* genes are conserved, although gene losses or gains were also identified. These results suggest that segmental duplication and whole-genome duplication played a major role in the expansion of the *BnLEA* gene family. Expression profiles analysis indicated that expression of most *BnLEA*s was increased in leaves and late stage seeds. This study presents a comprehensive overview of the *LEA* gene family in *B. napus* and provides new insights into the formation of this family.

Drought stress is an abiotic environmental state that can affect the morphological, physiological and biochemical characteristics of plants and lead to reductions in crop productivity due to adverse effects on plant growth^1^. Signaling pathways that are activated in response to drought challenge include ionic and osmotic stress signaling, detoxification signaling, and signaling of cell division coordination^2^. The expression of many signal transduction genes has been observed; for example, significant drought stress can induce *DREB2A* over-expression in transgenic *Arabidopsis*^3^. The *galactinol synthase* (GolS) genes of *Arabidopsis* are induced by drought and play a role in the accumulation of raffinose family oligosaccharides (RFOs), which might act as osmoprotectants in drought stress^4^.

Late embryogenesis abundant (LEA) proteins accumulate during late embryogenesis and contribute to drought tolerance^5^. In plants, LEA proteins are produced during the last period of seed development concurrent with dehydration. LEA proteins were first observed and studied in late-developing cotton seeds^6^ and were subsequently identified in many other plants, such as rice, barley, wheat, maize, bean, sunflower^7^ and *Arabidopsis*^8^. LEA proteins have also been identified in other species, such as nematodes^9^ and chironomids (*Polypedilum vanderplanki*)^10^. Subcellular localization analysis has revealed that LEA proteins are mainly located in nuclear regions and the cytoplasm^11^. Although LEA proteins are mainly observed in plant seeds, they have also been detected in the seedlings, buds and roots of plants^7^,^8^. In contrast to other proteins involved in desiccation tolerance, LEA proteins have no apparent enzymatic activity and likely act as protectants of biomolecules and membranes under stress conditions^11^. However, some studies have indicated that individual LEA proteins might function as intrinsically disordered proteins to protect enzymes from induced aggregation^12^,^13^. This protection may be due to space-filling by LEA proteins, referred to as the “molecular shield function”, which decreases the rate of collisions between aggregating proteins^14^. Moreover, LEA proteins contribute to the isolation of calcium and metal ions, which participate in signaling pathways in plants^15^. LEA proteins also aid the formation of the glassy state, in which nonreducing sugars accumulate in the cytoplasm of plants during periods of desiccation^16^. These finding imply that LEA proteins play a role in protecting plants from dehydration.

LEA proteins are low-molecular-weight proteins composed of hydrophilic amino acids and are characterized by repeat motifs, structural disorder and high hydrophilicity in their natural forms^7^,^8^,^17^. LEA proteins are classified into at least eight families in the Pfam database based on primary sequence and homology: LEA_1, LEA_2, LEA_3, LEA_4, LEA_5, LEA_6, dehydrin and seed maturation protein (SMP)^18^. In the LEAPdb database, these proteins are regrouped using a more detailed classification system, with 12 nonredundant classes^19^. Group 1–5 are considered the major members^17^. Group 1 proteins contain a 20-amino-acid motif (GGETRKEQLGEEGYREMGRK) and a high proportion of Gly, Glu and Gln residues^20^. A sequence called the K-segment (EKKGIMDKIKEKLPG), which functions as a chaperone to protect proteins that function in cell metabolism,^21^. Group 3 proteins have an 11-amino-acid (TAQAAKEKAGE) fragment with 13 repeats^17^. Group 4 contains no repeated motif sequences but features a conserved structure at the N-terminus that can form α-helical structure^7^. The amino acid residue homology of Group 5 proteins is low, which implies that these proteins are probably involved in seed maturation and dehydration^21^.

*Brassica napus* (AACC, 2n = 38) originated from hybridization between *Brassica rapa* (AA, 2n = 20) and *Brassica oleracea* (CC, 2n = 18). *B. napus* is the third largest oil seed crops in the world. Quite a few studies have been conducted on different groups of *LEA* genes in *B. napus* in recent years^22^,^23^,^24^. The Group 4 LEA protein of *B. napus* enhances abiotic stress tolerance in both *Escherichia coli* and transgenic *Arabidopsis* plants^22^. The dehydrin genes of *Brassica juncea* and *B. napus* are expressed at the late stages of silique development, suggesting that gene expression might be induced by water deficit and low temperatures, conditions that also affect seed germination^23^. Expression of the *B. napus* LEA protein gene in Chinese cabbage enhanced its growth ability under salt and drought stress^24^. Moreover, LEA proteins have been observed in *B. napus* lines with higher oil contents, suggesting that LEA proteins might contribute to dehydration tolerance during the oil-accumulation period and increased *B. napus* oil content^25^.

Because *B. napus* is a hybrid species, its genome contains many duplications as well as inversions and translocations^26^. Previous studies have mainly focused on the function of different LEA families, and an analysis of the evolution, distribution and origin of the *LEA* gene family in *B. napus* has not been reported. In this study, the *LEA* gene families in *B. napus* were identified, and the structure, evolution and chromosome location of *BnLEA*s were analyzed. This study provides a foundation for further studies of the functions of the LEA family in *B. napus*.

## Results

### Genome-wide identification of *BnLEA* gene families in *B. napus*

**Figure 1:**
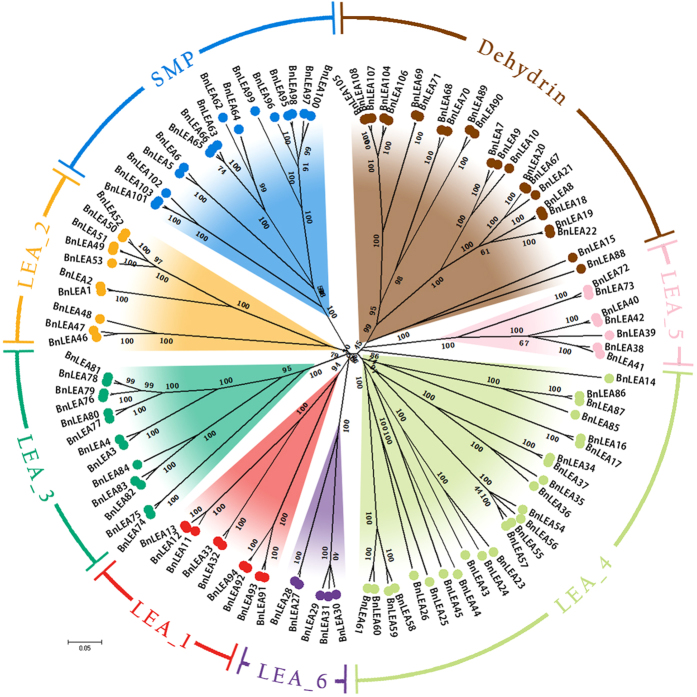
Phylogenetic analysis of the *B. napus LEA* genes. LEA gene families are distinguished by different colors. The unrooted tree was generated using ClustalW in MEGA6 using the full-length amino acid sequences of the 108 *B. napus* LEA proteins.

The genome-wide identification of *LEA* gene families in *B. napus* was based on homology with *LEA* genes from *Arabidopsis* identified using the CNS-Genoscope database. A total of 108 *LEA* genes were identified in the genome of *B. napus* and named *BnLEA1* to *BnLEA108* (Table 1). The *BnLEA* genes were classified into eight families based on their conserved domain structures. The LEA_4, dehydrin and seed mature protein (SMP) families are the largest (25, 23 and 16 members, respectively) among the families (Fig. 1). The LEA_2 and LEA_3 family include 10 and 13 members, respectively. Fewer than 10 members of the other families were identified. *LEA* genes were also identified in sixteen other species, including both lower and higher plant species (Figure S1). Only two *LEA* genes were identified in the bacillariophyta. Vascular plants (except cotton) have more *LEA* genes than *Physcomitrella patens*, implying that *LEA* genes accumulated during the landing process^27^. Interestingly, in nearly half of the species containing *LEA* genes, the majority belong to the LEA_4 and dehydrin families, consistent with the predominance of the LEA_4 and dehydrin families in *B. napus*.

The physicochemical parameters of each *LEA* gene were calculated using ExPASy. Most proteins in the same family have similar parameters. BnLEAs of the dehydrin family contain a greater number of amino acid residues (except for BnLEA15) than the other LEAs. LEA_6 family members all have relatively low molecular masses (Table 1). Approximately two-thirds of the BnLEA proteins have relatively low isoelectric points (pI < 7), including the LEA_2, LEA_5, LEA_6 and SMP families. The remaining proteins, particularly those in the LEA_1 and LEA_3 families, have pI > 7. The grand average of hydropathy (GRAVY) value was defined by the sum of the hydropathy values of all amino acids divided by the protein length (Table 1). LEA_2 proteins are the most hydrophobic, and LEA_5 members are the most hydrophilic; these results are consistent with those of the LEA proteins in *Arabidopsis*^8^. Nearly all of the BnLEAs are hydrophilic, with a GRAVY value <0, indicating that a large proportion of the LEA proteins are hydrophilic. Because low hydrophobicity and a large net charge are features of other LEA proteins^8^,^28^,^29^ that allow them to be “completely or partially disordered”, these proteins may form flexible structural elements such as molecular chaperones that contribute to the protection of plants from desiccation^30^,^31^. TargetP and PProwler were used to predict the subcellular location of 108 BnLEA proteins; most of the BnLEA proteins were predicted to participate in the secretory pathway (Table S1).

### Sequence alignment and phylogenetic analysis of *BnLEA* genes

To determine the similarity and homology of the *BnLEA* genes, sequence alignments were performed, and an unrooted phylogenetic tree of the 108 *BnLEA* genes was constructed (Fig. 1). Gene pairs frequently appeared in the whole genome of *B. napus* (Fig. 1). Little similarity was observed among the eight families. The sequences of the *BnLEA* genes of the LEA_6 family are most highly conserved (Table S2, Figure S3). By contrast, the *BnLEA* genes of the LEA_4 family feature only 17.5% consensus positions, and nearly no identical positions were observed (Table S2, Figure S3). The other families exhibit moderate homology (Table S1, Figure S3). Every family contains the conserved regions. The dehydrin, LEA_4, LEA_6 and LEA_1 families all contains three regions of homology. Two such regions were detected in the LEA_2 and LEA_5 families, and four are present in the LEA_3 family (Figure S2). Interestingly, a large number of alanine residues is present in all LEA families. Lysine and glutamic acid are the second- and third-most abundant residues, respectively, in all BnLEA proteins. A large number of glycine residues are widely present in the LEA_2, LEA_5, LEA_6, SMP and dehydrin families (Figure S2). These amino acids are also abundant in other LEA proteins and may contribute to the function of LEAs in the protection of many enzymes on the membrane^5^,^19^,^20^.

Although the different families exhibit low similarity, they cluster into eight major clades (Fig. 1). As expected, the *BnLEA* genes of LEA_3, LEA_2, SMP, LEA_6, and LEA_1 cluster into a separate branch. However, *BnLEA14*, which contains an LEA_4 domain, clusters into another clade, closer to the LEA_5 family (Fig. 1). The genetic relationship between BnLEA14 proteins and LEA_5 family proteins may have become closer during many years of evolution (Fig. 1). The analysis demonstrated that although the LEA_1 and LEA_ 6 families contain different conserved domains, they might have evolved to a closer relationship during evolution. Forty sister pairs of genes were identified in the phylogenetic trees with very strong bootstrap support (100%). Another three pairs of genes also had relatively high bootstrap support (90–99%) (Fig. 1). Most of the gene pairs had short branch lengths, suggesting recent divergence (Fig. 1). These findings indicate that gene pairs are relatively common among the 108 *LEA* genes of *B. napus*. During evolution, the conserved areas have been preserved, but several variations have also occurred, enabling the division of some genes into subfamilies.

### Structural analysis of *BnLEA* genes

**Figure 2:**
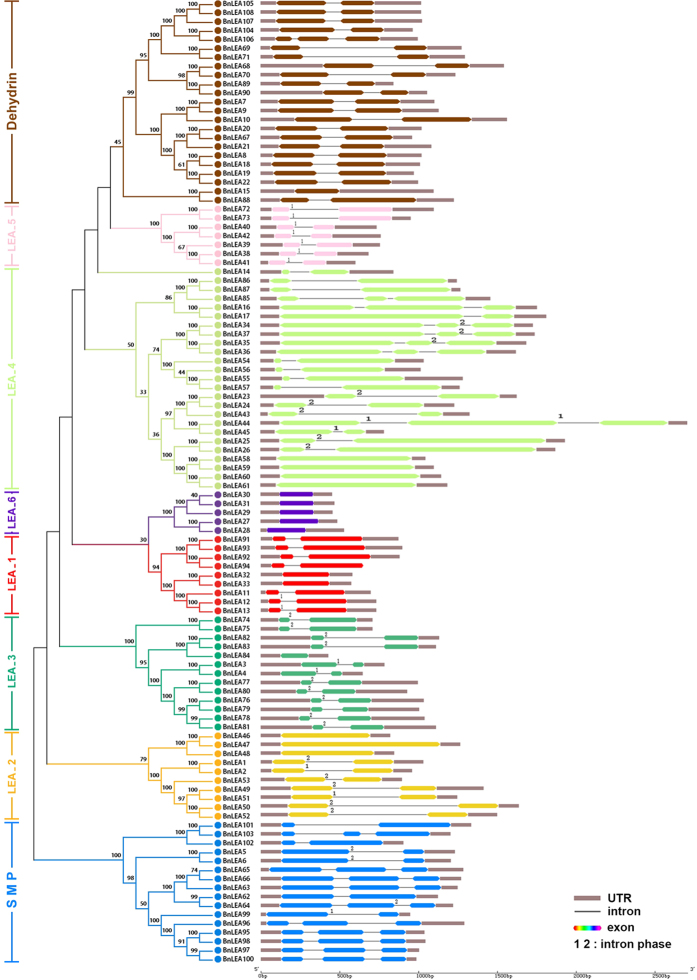
Exon–intron organization of the *BnLEA* genes. Double-sided wedge boxes represent exons, and different colors indicate different *LEA* gene families. Black lines represent introns, and untranslated regions (UTRs) are indicated by light-gray purple boxes. Numbered marks represent the splicing phases. Phase-0 is not marked. The exon and intron sizes can be estimated using the scale at the bottom.

To characterize the structural diversity of the *BnLEA* genes, exon-intron organization analysis of the individual *BnLEA* genes was performed, and some genes from each family used in the conserved domain analysis or motifs model structure were selected for further research. The majority of the LEA genes contain two or three exons, whereas members of the LEA_6 family have only one intron, and the 16 *BnLEA* genes have no introns (Fig. 2). A high proportion of the introns in the *BnLEA* genes are in phase-0 (interrupted by exactly two triplet codons). All members of the LEA_5 family and some members of other families contain phase-1 introns (separated by the first and second nucleotides of a codon). Twenty-five phase-2 introns (split by the second and third nucleotides of a codon) were observed. The majority of phase-2 introns were observed in the LEA_3 family (Fig. 2). Most of the closely clustered LEA genes in the same families have similar exon numbers and intron lengths. By examining the exon-intron organization and paralogous pairs of LEA genes that clustered together at the terminal branch of the phylogenetic tree, various exon-intron changes were identified. Six pairs of *BnLEA* genes exhibit exon-intron gain/loss variations (*BnLEA15*-*BnLEA88*, *BnLEA16-BnLEA17*, *BnLEA44*-*BnLEA45*, *BnLEA104-BnLEA106*, *BnLEA103*-*BnLEA101*, *BnLEA62*-*BnLEA64*), possibly due to a single intron loss or gain event during the long evolution process^32^.

**Figure 3:**
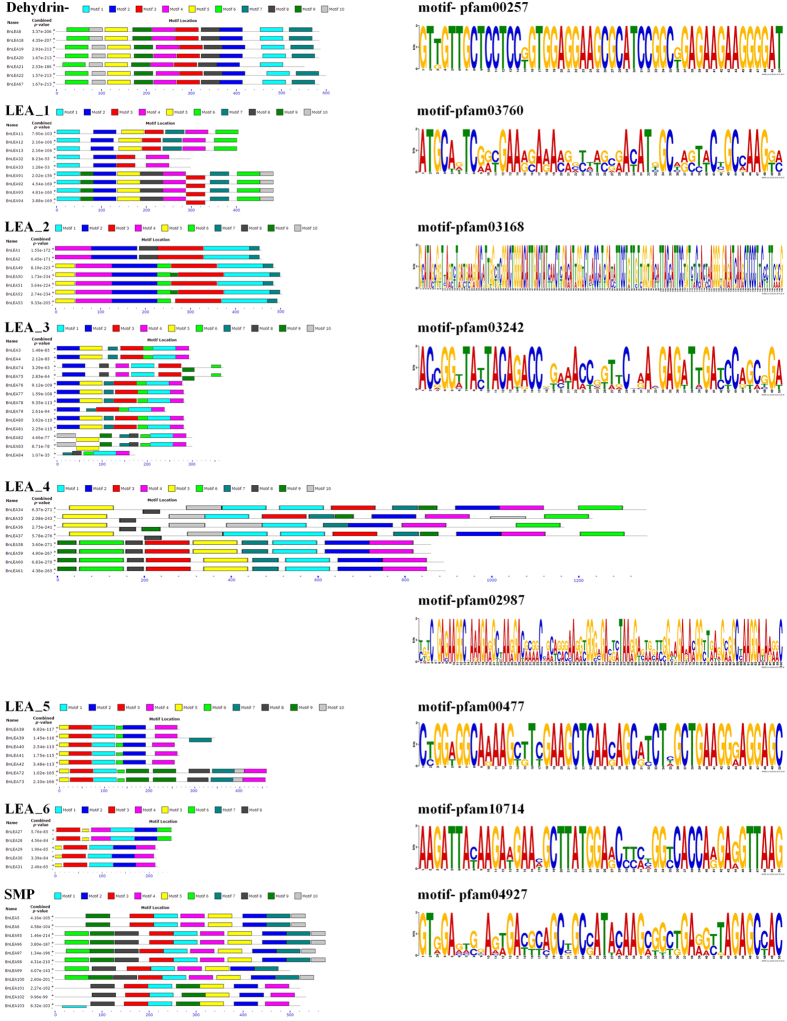
Motif patterns of different *BnLEA* families and WebLogo plot of consensus motifs in each *BnLEA* gene family. Representative *B. napus* LEA proteins were selected for alignment, and LEA motifs are shown as motif 1 (light blue box). The lengths of the proteins and motifs can be estimated using the scale at the bottom. The Pfam codes of the LEA motifs of each family are shown.

Because the 108 *BnLEA* genes did not share high similarity, several typical genes of each family were submitted to MEME for domain or motif structure analysis. Ten motifs were identified as conserved motifs. Motif 1, which was present in every family, encodes a conserved LEA domain, as indicated by the Pfam codes and WebLogo (Fig. 3). Most of the closely related genes in each family exhibit similar motif compositions, suggesting functional similarities in the LEA family. Motif 1s of the LEA_2 family is the biggest motif. The LEA_6 family has the lowest number of motifs, only five or six(Fig. 3). These results imply that the composition of the structural motifs varies among different LEA families but is similar within families and that the motifs encoding the LEA domains are conserved.

### Chromosomal location and duplication pattern analysis of *BnLEA* genes

**Figure 4:**
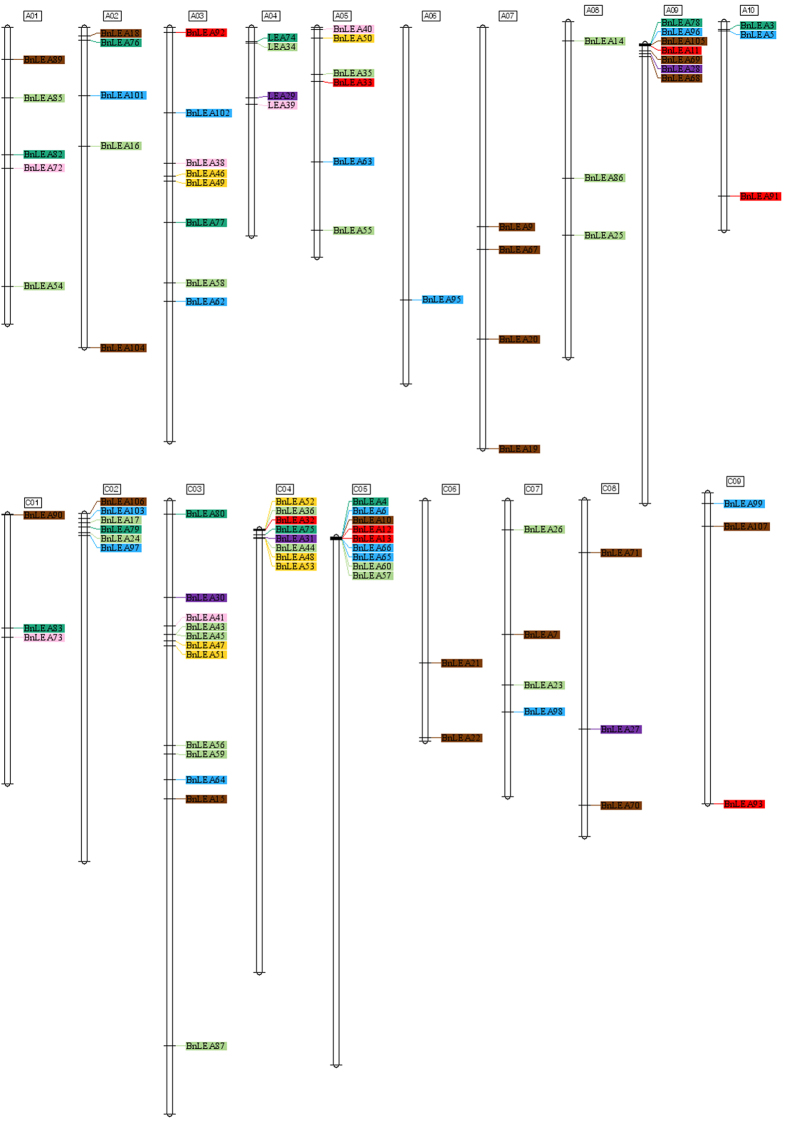
Distribution of *BnLEA* gene family members on *B. napus* chromosomes. The 96 *BnLEA* genes for which exact chromosomal information was available in the database were mapped to the 19 *B. napus* chromosomes. The color of each gene indicates the corresponding family.

The chromosomal location of the LEA genes was analyzed, and the positions and chromosome locations of 96 *BnLEA* genes were clearly identified on the 19 chromosomes of *B. napus* (Table 1, Fig. 4). The number of *BnLEA* genes varies considerably among the different chromosomes, and chromosomes C3 and A6 contain the greatest (n = 12) and lowest (n = 1) numbers, respectively (Fig. 4). In general, genes belonging to the same family are distributed in different chromosomes to realize full functionality. Interestingly, genes of the dehydrin and LEA_4 families are only located on chromosomes A7, C6 and A8, suggesting that these genes have a tendency to duplicate and evolve more conservatively within one chromosome. High-density *LEA* gene clusters were identified in certain chromosomal regions, e.g., at the top of chromosomes A9, C2, C4, and C5 and in the middle of chromosome C3 (Fig. 4). Thus, the final chromosomal locations of the *LEA* genes may be the result of *LEA* gene duplication patterns.

Gene family expansion occurs via three mechanisms: tandem duplication, segmental duplication and whole-genome duplication (WGD)^33^. The progenitor diploid genomes of *B. napus* are ancient polyploids, and large-scale chromosome rearrangements have occurred since their evolution from a lower chromosome number progenitor^34^. Duplicated regions of the *Arabidopsis* genome occur 10 to 14 times within the *B. napus* genome^35^. Moreover, chromosomal gene location and homology synteny analyses have revealed that *BnLEA* genes are closely phylogenetically related to other *Brassicaceae* species (*B. rapa*, *B. oleracea*, *Arabidopsis*) and that *Brassicaceae* experienced an extra whole-genome triplication (WGT) event^32^. Tandem duplications and segmental duplications also played an important role in the model plant *Arabidopsis*^36^. We investigated gene duplication events to understand the genome expansion mechanism of the *BnLEA* gene superfamily in *B. napus*. Six tandemly duplicated genes (*BnLEA43*/*BnLEA45*, *BnLEA12*/*BnLEA13*, and *BnLEA66*/*BnLEA65*) located on chromosomes C3 and C5 were identified (Fig. 4, Table 1). All 108 *BnLEA* genes in the *Brassica* database were reviewed, and the results revealed that nearly two thirds of the *BnLEA* genes are associated with segmental duplications. Two loci (At1g32560 and At3g22490) have three copies involved in segmental duplications (Table 2). Comparing the distributions of genes around the LEA genes in the genomes of *A. thaliana*, *B. oleracea*, *B. rapa* and *B. napus* revealed that the synteny of the LEA_1, LEA_2, LEA_3, LEA_4, SMP and dehydrin families is preserved, along with some genes that were lost or duplicated (Figure S4), whereas the synteny of the LEA_5 and LEA_6 families is poor.

**Figure 5:**
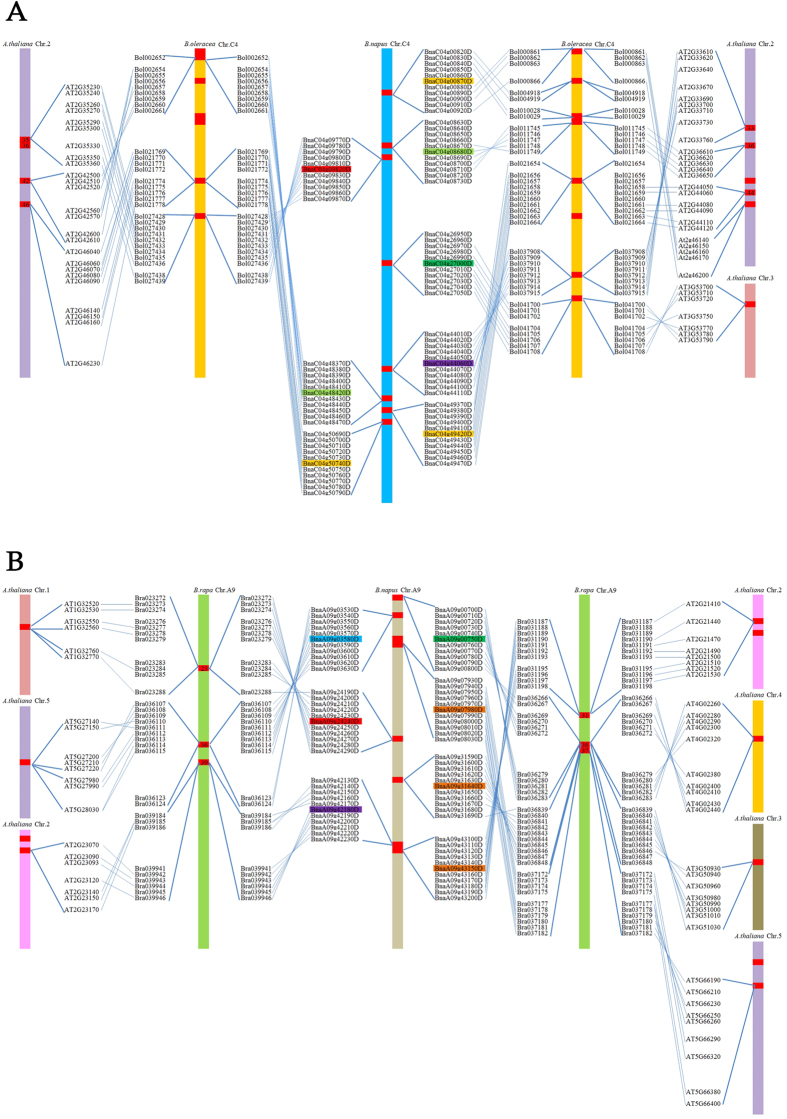
Synteny analysis map of gene clusters in *B. napus* chromosomes. **(A**) Genes located on *B. napus* chromosome C4 are syntenic with genes of *B. oleracea* and *A. thaliana*. (**B**) Genes located on *B. napus* chromosome A9 are syntenic with genes of *B. rapa* and *A. thaliana*. The different colors of the gene IDs indicate their individual LEA families (brown: dehydrin; light green: LEA_4;bottle green: LEA_3; red: LEA_1; purple: LEA_6; blue: SMP).

**Figure 6:**
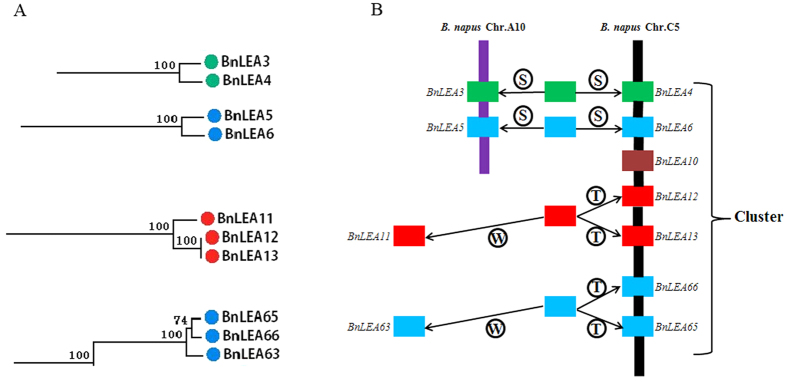
Phylogenetic relationships and hypothetical evolutionary progress of the clustering of *BnLEA* genes in *B. napus* chromosome C5. **(A**) Phylogenetic relationships of selected *BnLEA* genes in the cluster. (**B**) Hypothetical mechanism of *BnLEA* gene cluster formation. The letters T, S, and W in the schematic diagram of the hypothetical origins of *BnLEA* genes indicate putative tandem duplication, segmental duplication and whole-genome duplication, respectively.

Furthermore, the synteny maps of the genes in the clusters located in chromosomes A9 and C4 revealed the process of gene expansion and clustering (Fig. 5). In chromosome C4, the genes in two *A. thaliana* chromosomes (chromosomes 2 and 3) were linked to *B. oleracea* genes and were accompanied by gene expansion in *B. napus* (Fig. 5A). Among the analyzed genes, nearly half of them contained crossovers. In chromosome A9, the homologous *A. thaliana* LEA genes are distributed in all five *Arabidopsis* chromosomes. The clustering progress from *A. thaliana* to *B. rapa* is more obvious (Fig. 5B), and all groups of genes linked to *B. napus* contain crossovers, suggesting that the crossover events occurred during the allopolyploidy progress. The *BnLEA* gene clusters likely formed via the duplication of an ancestral gene during the WGD event, followed by tandem duplication and segmental duplication in the clusters. In the cluster of chromosome C5, *BnLEA66*/*BnLEA65* and *BnLEA12*/*BnLEA*13 are tandem duplication genes, although phylogenetic analysis regrouped *BnLEA63* and *BnLEA11* together with these genes, respectively, indicating that these genes might have descended from a common ancestor (Fig. 6A). Moreover, in the gene cluster, *BnLEA4* and *BnLEA6* are associated with segmental duplication because they exhibitsynteny relationships with *BnLEA3* and *BnLEA5*, respectively. Phylogenetic analysis also demonstrated that *BnLEA3*/*BnLEA4* and *BnLEA5*/*BnLEA6* are pairs of homologous genes, suggesting that the four genes might have descended from two ancestors. Interestingly, *BnLEA3* and *BnLEA5* are located in close proximity on chromosome A10, which implies that segmental duplication also played a role in LEA gene cluster formation (Fig. 6B).

Synonymous (Ks) and nonsynonymous (Ka) values were used to explore the selective pressure on duplicated *BnLEA* genes. In general, a Ka/Ks ratio greater than 1 indicates positive selection, a ratio less than 1 indicates functional constraint, and a Ka/Ks ratio equal to 1 indicates neutral selection^37^. The orthologous *LEA* gene pairs between the *B. napus* and *A. thaliana* genomes were used to estimate Ka, Ks, and Ka/Ks (Table 2). The results revealed that most of the *BnLEA* genes have Ka/Ks ratios greater than 0.1. However, the lowest Ka/Ks ratio is only 0.0179 (*BnLEA48*), and the highest Ka/Ks ratio is 0.6434 (*BnLEA3*). The genes of the LEA_3 and LEA_6 families exhibit relatively high Ka/Ks ratios, whereas the LEA_2 and LEA_5 gene families have lower Ka/Ks ratios. The Ka/Ks ratios of the other families are 0.2–0.3. These findings indicate that LEA_2 and LEA_5 genes might preferentially conserve function and structure under selective pressure.

### Expression profiles analysis of *BnLEA* genes in different tissues

**Figure 7:**
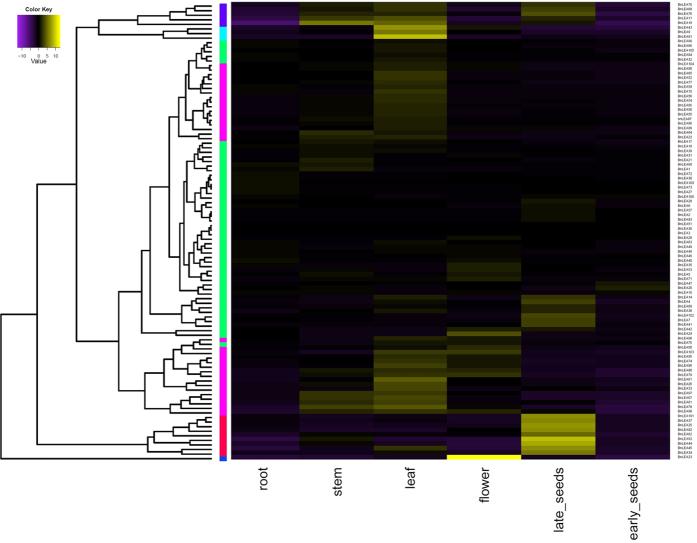
Hierarchical clustering of the expression profiles of *BnLEA* genes in different tissues. The log-transformed values of the relative expression levels of *BnLEA* genes were used for hierarchical cluster analysis (original data shown in Table S4). The color scale represents relative expression levels with increased transcript (yellow) or decreased transcript (purple). Early_stage seeds were got 19 weeks after seeding, late_stage seeds were 40 weeks after seeding.

To investigate the expression pattern of *LEA* genes in *B. napus*, the qRT-PCR of *BnLEA*s genes were performed. The present results indicated that the accumulation of *BnLEA* genes was associated with different tissues, and the expression pattern also differed among each *LEA* gene family (Fig. 7). Pair-wise genes showed similar expression pattern, further analysis revealed that the expression of more than two thirds of *BnLEA*s were increased in leaf, especially *BnLEA91* and *BnLEA43*. Compared with the early developmental stage seeds (19 weeks after seeding), late stage developmental seeds (40 weeks after seeding) showed much higher expression level of *BnLEA*s, for example, *BnLEA93* and *BnLEA34*. Leaves are sensitive tissues in stress environment, they become wilt or died in stress condition and affect the photosynthesis in plants^1^. Late developmental stage seeds frequently suffered from dehydration, the present reported high expression of *BnLEA* genes in the late developmental stage seeds was consistent with reported LEA protein function^7^. Interestingly, some phylogenetic gene pairs have different expression pattern (*BnLEA7*/*BnLEA9*, *BnLEA25*/*BnLEA26*, *BnLEA60*/*BnLEA61*, *BnLEA91*/*BnLEA93*). The result suggests even if these genes contain close phylogenetic relationship they may develop different biological function.

## Discussion

LEA gene family has been reported in many crops, such as rice and maize^7^. However, the genome-wide identification and annotation of LEA genes has not been reported in *B. napus*. In this study, 108 LEA family genes were identified in *B. napus*. The *BnLEA* gene family is larger than LEA families in homologous crucifer plants, such as *B. oleracea* (40 *LEA* genes), *B. rapa* (66 *LEA* genes) and *A. thaliana* (51 *LEA* genes). *B. napus* originated from the hybridization of *B. oleracea* and *B. rapa*, and its assembled genome size is larger than that of *B. oleracea* (540 Mb) and *B. rapa* (312 Mb)^26^. The preservation of LEA genes during a polyploidy event suggests that these genes play important roles in plant development^7^,^24^,^27^,^28^.

In general, genes that respond to stress contain fewer introns^28^. Confirming this assumption, 92 of the 108 *BnLEA* genes have no more than two introns. Low intron numbers have also been observed in other stress-response gene families, such as the trehalose-6-phosphate synthase gene family^38^. Introns can have a deleterious effect on gene expression by delaying transcript production. Moreover, introns can extend the length of the nascent transcript, resulting in an additional expense for transcription^39^.

The motif numbers and composition of each family vary, although some amino acid-rich regions were detected, similar to the Gly-rich region in *Arabidopsis* LEA_2 proteins, and the most-conserved motif is rich in lysine (K) residues^8^. The amino acid composition of the LEA proteins suggests disordered structure along their sequences^5^,^40^. Although LEA proteins are relatively small and intrinsically unstructured, they play important roles in cells^30^, likely by forming flexible, residual structural elements^30^, such as α-helical structures and polyproline II (PII) helices^41^. These elements contribute to structural flexibility and thus enable proteins to bind DNA, RNA, and proteins as interaction partners^31^. Conformational changes may facilitate interactions between LEA proteins and other macromolecules, such as membrane proteins, to maintain cell stability^42^. These results demonstrate that LEA proteins feature unique conserved amino acid-rich regions and an unstructured form that allow LEA proteins to function as flexible interactors to protect other molecules under stress conditions^8^,^30^,^31^.

Gene duplication not only expands genome content but also diversifies gene function to ensure optimal adaptability and evolution of plants^33^. *Brassica* species have undergone WGD events during their evolution^32^, and *B. napus* was formed by allopolyploidy^26^. Several independent lineage-specific WGD events have been identified in *Brassicaceae*^35^,^43^. In this study, only 6 tandemly duplicated genes were identified, by contrast to LEA genes in *Prunus mume* (tandem duplication = 40%), perhaps because WGD did not occur in this species^29^. Most *BnLEA* genes showed a close relationship with respect to the block locations of *Arabidopsis LEA* genes. Phylogenetic and homology analyses suggested that WGD contributed to *BnLEA* gene family expansion. WGD has also been observed in the LEA family of another *Brassicaceae* species (*Arabidopsis*)^21^. The *Arabidopsis* genome contains 51 *LEA* gene family members^8^; therefore, a WGT event would be expected to produce more than 150 *LEA* genes in the *B. rapa* or *B. oleracea* genome, ultimately leading to even more *LEA* genes in *B. napus*. However, only 108 genes remain in the *B. napus* genome. This new finding implies that more than 50% of duplicated *LEA* genes were lost after WGT, likely due to extensive chromosome reshuffling during rediploidization after WGT. In fact, natural selection drove the rediploidization process via chromosomal rearrangement, thus removing extra homologous chromosomes, and further rounds of genomic reshuffling of the rediploid ancestor occurred at different evolutionary time points to create the different species of *Brassica*^32^. The number of *LEA* genes was possibly sufficient for *Brassica* during the long natural selection process, and thus some duplicated *LEA* genes did not remain in the *B. napus* genome. Similar deletions or losses of genes after WGT have been observed in the NBS-encoding genes of *Brassica* species^44^. Segmental duplication also plays a role in *BnLEA* superfamily expansion, and 72 *BnLEA* genes were determined to have one or two close relatives in the corresponding duplicated regions. Therefore, 66% (72/108) of the *BnLEA* genes can be accounted for by segmental duplication. This finding is similar to observations of the *LEA* gene family of *Arabidopsis* (12 pairs of 51 genes)^8^. Synteny analysis demonstrated that most LEA gene family members are located in well-conserved synteny regions, and some genes were deleted or gained. These findings indicate that some genes might have been translocated into a non-syntenic region. Similarly collinear genomic regions with some deleted genes have been identified in other gene families^44^. These present and previous findings suggest that segmental duplications and WGD likely played an important role in the expansion of the LEA family in *B. napus*, even though some genes were lost after WGT. Tandem duplication was also identified but played only a minor role.

As discussed above, WGD and segmental duplication may be the main mechanisms underlying the expansion of the *B. napus* LEA gene family. During duplication, mutational targets may increase, and some genes are convergently restored to single-copy status^45^. In this study, genes of the A genomes from *B. rapa* and C genomes from *B. oleracea* exhibited greater homology to *B. napus* than to *A. thaliana*. A clustering phenomenon was also observed, accompanied by the loss or gain of some genes. Gene clustering has also been observed in the *LEA* gene families of other species^28^. Synteny analysis of *LEA* gene clustering in C4 and A9 revealed that some translocation and inversion events occurred during the evolution of *A. thaliana*, *B. rapa*, *B. oleracea* and *B. napus*. These events may have been the result of chromosomal rearrangement during the evolution of *Brassica*^32^. The formation of *LEA* gene clusters might have been affected by the subgenome dominance effect, resulting in one subgenome that retained more genes via gene fractionation after WGT^32^,^46^. This hypothetical *BnLEA* gene clustering mechanism is similar to that identified in the LEA family of *Populus*^28^. First, the WGD event promoted genomic reshuffling accompanied by chromosome reduction, which contributed to the speciation of diploid *Brassica* plants. Genomic differentiation of the three basic genomes then generated the stable allotetraploid species *B. napus*. Second, after WGD, biased gene retention via gene fractionation and multicopy gene appearance promoted the gene-level evolution of *Brassica* species^26^,^32^,^46^. This proposed mechanism of *BnLEA* gene cluster formation reflects both the WGD effect during the evolution of *Brassica* species and other duplications after WGD that resulted in the abundant morphotypes and genotypes of *Brassic*a species.

Genomic comparison is a rapid means of obtaining knowledge about less-studied taxon^35^. Many studies have revealed that *LEA* genes contribute to abiotic stress tolerance, particularly to drought stress^7^,^16^. According to the expression pattern of *BnLEA* genes in different tissues, it would be interesting to functionally characterize these genes in *B. napus*. As many *BnLEA* genes showed higher expression level in same tissue (leaf and late developmental stage seeds), which indicated the functional conservation of this gene family. Some of the *BnLEA* genes were more abundant in different tissues, which point toward their functional differences. Similar expression pattern were also observed in other gene families of *Brassica* species^44^. Therefore, existing knowledge on the function of *LEA* genes may explain why the *LEA* gene family expanded in terrestrial plants but not algae because algae are rarely exposed to drought stress^27^. LEA families with close taxonomic relationships generally exhibit similar scales and distributions. However, the scales of the *LEA* gene family differ in maize and rice. Due to variations in the evolutionary rates of the whole genomes of grasses, which are subject to broad changes in environmental conditions, maize exhibits divergence signals that are associated with directionally selected traits and are functionally related to stress responses. These results suggest that stress adaptation in maize might have involved the evolution of protein-coding sequences^47^. Additionally, these evolutionary changes probably led to the observed differences in the *LEA* gene families of maize and rice. In *Brassicaceae*, *Br*LEAs, *Bo*LEAs and *Bn*LEAs are homologous to *At*LEAs. In the *A. thaliana* genome, chromosomes are divided into 24 blocks^48^. The chromosomal locations of the *BnLEA* genes exhibit a genomic block distribution similar to that of the *LEA* genes in *A. thaliana*^21^. *B. napus* inherited most of its genes in the homologous genomic blocks of *B. oleracea* and *B. rapa*. The chromosome evolution of *Brassica* plants involved these genomic blocks^32^. The WGT events promoted gene-level and genomic evolution, thus contributing to the diversification of *Brassica* plants^32^,^48^. The evolution of *LEA* gene families in *Brassicaceae* is part of the long history of evolution of *Brassicaceae* species.

In conclusion, a total of 108 LEA genes were identified in *B. napus* and classified into eight groups. Chromosomal mapping and synteny analysis revealed that 108 *BnLEA* genes were distributed in all *B. napus* chromosomes with some gene clustering. Segmental duplication and WGD were identified as the main patterns of LEA gene expansion in *B. napus*. The *BnLEA* genes all contain the LEA motif and have few introns. Genes belonging to the same family exhibit similar gene structures, consistent with their Ka/Ks ratios. This current increases our understanding of LEA genes in *B. napus* and lays the foundation for further investigations of the functions of these LEA proteins in oilseed rape.

## Methods

### Identification of LEA family genes in *B. napus* and other species

LEA genes were identified in *B. napus* based on homology with the 51 LEA protein sequences from Arabidopsis^8^ using the BLAT search program in the CNS-Genoscope database (http://www.genoscope.cns.fr/brassicanapus/)^26^. Redundant sequences were removed manually. All *BnLEA* gene candidates were analyzed using the Hidden Markov Model of the Pfam database (http://pfam.sanger.ac.uk/search)^18^, SMART database (http://smart.embl-heidelberg.de/)^49^, and NCBI Conserved Domain Search database (http://www.ncbi.nlm.nih.gov/Structure/cdd/wrpsb.cgi)^50^ to confirm that each gene was a member of LEA family. Using the Pfam nomenclature, the LEA gene family of *B. napus* was divided into eight groups: LEA_1 to LEA_6, SMP and dehydrin. A univocal name consisting of two italic letters denoting the source organism, the family name, and subfamily numeral for each gene was assigned to each LEA gene (e.g., *BnLEA1*).

To trace the evolutionary origin of the LEA gene family in plants, LEAs were identified in other plant species using Phytozome (http://phytozome.jgi.doe.gov/pz/portal.html)^8^,^28^, including *Oryza sativa*, *Zea mays*, *Gossypium hirsutum*, *Glycine max*, *Arabidopsis thaliana*, *Brassica rapa*, *Brassica oleracea*, *Selaginella moellendorffii*, *Physcomitrella patens*, *Thalassiosira pseudonana*, *Vitis vinifera*, *Populus trichocarpa* and *Setaria italica*. Finally, sixteen species were chosen, including three green algae, a moss, two lycophytes, three gramineae, four cruciferae, grape, populus, cotton and soybean.

The number of amino acids, CDS lengths and chromosome locations of the *BnLEA* genes were obtained from the *B. napus* database. The physicochemical parameters, including molecular weight (kDa) and pI, of each BnLEA protein were calculated using the compute pI/Mw tool of ExPASy (http://www.expasy.org/tools/). GRAVY (grand average of hydropathy) values were calculated using the PROTPARAM tool (http://web.expasy.org/protparam/)^51^. Subcellular location prediction was conducted using the TargetP1.1 (http://www.cbs.dtu.dk/services/TargetP/) server^52^ and Protein Prowler Subcellular Localisation Predictor version 1.2 (http://bioinf.scmb.uq.edu.au/pprowler_webapp_1-2/)^53^.

### Multiple alignment and phylogenetic analysis of *BnLEA* family genes

Multiple sequence alignment of all predicted BnLEA protein sequences was performed using ClustalW software. An unrooted phylogenetic tree of the 108 full-length LEA protein sequences was constructed using MEGA 6 with the Neighbor Joining (NJ) method, and bootstrap analysis was conducted using 1,000 replicates^54^,^55^.

### Gene structure analysis of *BnLEA* family genes

The exon-intron structures of the *BnLEA* family genes were determined based on alignments of their coding sequences with the corresponding genomic sequences, and a diagram was obtained using GSDS (Gene structure display server: GSDS: http://gsds.cbi.pku.edu.cn/)^56^. MEME (Multiple Expectation Maximization for Motif Elicitation) (http://alternate.meme-suite.org/) was used to identify the conserved motif structures encoded by the *BnLEA* family genes^57^. In addition, each structural motif was annotated using Pfam (http://pfam.sanger.ac.uk/search)^18^ and SMART (http://smart.embl-heidelberg.de/) tools^49^. To confirm the gene structures, all 108 *BnLEA* gene sequences were queried against published transcriptome RNA-seq data from *B. napus* in the NCBI database using BLAST (all genes sequence were consistent with No. ERX515977, ERX515976, ERX515975, ERX515974, or ERX397800 transcriptome data)^26^,^58^.

### Chromosomal location and gene duplication of *BnLEA* family genes

The chromosomal locations of the *BnLEA* genes were determined based on the positional information obtained from the *B. napus* database. Tandemly duplicated *LEA* genes were defined adjacent to homologous *LEA* genes on *B. napus* chromosomes or within a sequence distance of 50 kb^44^. The synteny relationships between the *BnLEA*s and *A. thaliana* LEAs, *B. rapa* LEAs, and *B. oleracea* LEAs were evaluated using the search syntenic genes tool in BRAD (http://brassicadb.org/brad/)^46^ and synteny tools of the *B. napus* Genome Browser (http://www.genoscope.cns.fr/brassicanapus/cgi-bin/gbrowse_syn/colza/)^26^.

### Calculation of the Ka/Ks values of *BnLEA* family genes

The LEA gene sequences of each paralogous pair were first aligned using ClustalW. The files containing the multiple sequence alignments of the LEA gene sequences were then converted to a PHYLIP alignment using MEGA 6. Finally, the converted sequence alignments were imported into the YN00 program of PAML to calculate synonymous and non-synonymous substitution rates^59^.

### RNA extraction and qRT-PCR analysis

An RNAprep Pure Plant Kit (Tiangen) was used to isolate total RNA from each frozen sample and first-strand cDNA was synthesized from the RNA by using a PrimeScriptTM RT Master Mix Kit (TaKaRa) according to the manufacturer’s instructions. Gene-specific primers were designed by using Primer5.0 (Table S3). Each reaction was carried out in triplicate with a reaction volume of 20 μl containing 1.6 μl of gene-specific primers (1.0 μM), 1.0 μl of cDNA, 10 μl of SYBR green(TaKaRa), and 7.4 μl sterile distilled water. The PCR conditions were as follows: Stage 1: 95 °C for 3 min; stage 2: 40 cycles of 15 s at 95 °C and 45 s at 60 °C; stage 3: 95 °C for 15 s, 60 °C for 1 min, 95 °C for 15 s. At stage 3, a melting curve was generated to estimate the specificity of the reactions. A housekeeping gene (*actin*) constitutively expressed in *B. napus* was used as a reference for normalization and analzsed by using an ABI3100 DNA sequencer (Applied Biosystems; Quantitation-Comparative: ΔΔCT)^60^.

## Additional Information

**How to cite this article**: Liang, Y. *et al.* Genome-wide identification, structural analysis and new insights into late embryogenesis abundant (*LEA*) gene family formation pattern in *Brassica napus*. *Sci. Rep.* **6**, 24265; doi: 10.1038/srep24265 (2016).

## Supplementary Material

Supplementary Information

## Figures and Tables

**Table 1:** *LEA* genes in *B. napus* genome and their sequence characteristics and subcellular location prediction.

Name	Gene ID	Family	Chr.	Gene position		Gene Length(bp)	Protein Length(aa)	Mol.Wt.(KD)	pI	GRAVY	Intron number	Subcellular location	
				Start	End							PProwler	TargetP
BnLEA1	BnaAnng17910D	LEA_2	Un-R	18849866	18850848	983	151	16.40492	4.72	0.0748344	1	other	O
BnLEA2	BnaCnng23520D	LEA_2	Un-R	21942981	21943895	915	151	16.40891	4.72	0.086755	1	other	O
BnLEA3	BnaA10g01410D	LEA_3	A10	749378	750171	794	98	10.29859	8.03	−0.228571	1	SP	S
BnLEA4	BnaC05g01450D	LEA_3	C5	756845	757576	732	98	10.25355	9.16	−0.210204	1	SP	S
BnLEA5	BnaA10g01720D	SMP	A10	859776	860870	1094	177	18.34329	4.55	−0.380791	1	SP	S
BnLEA6	BnaC05g01750D	SMP	C5	889027	890157	1010	177	18.31227	4.62	−0.356497	1	SP	S
BnLEA7	BnaC07g15380D	Dehydrin	C7	21301154	21302253	1100	216	4.45179	4.95	−1.388889	1	SP	O
BnLEA8	BnaAnng29030D	Dehydrin	Un-R	33214619	33215637	1019	194	21.8735	5.62	−1.321134	1	other	O
BnLEA9	BnaA07g11450D	Dehydrin	A7	10640882	10642008	1127	220	24.87639	5.07	−1.415455	1	SP	O
BnLEA10	BnaC05g15780D	Dehydrin	C5	9625056	9626614	1559	271	31.0062	5.09	−1.494096	1		O
BnLEA11	BnaA09g24240D	LEA_1	A9	17006117	17006806	690	133	14.49749	9.24	−0.87218	1	other	S
BnLEA12	BnaC05g24660D	LEA_1	C5	19159326	19160050	725	132	14.41236	9.24	−0.873485	1	other	S
BnLEA13	BnaC05g24760D	LEA_1	C5	19207889	19208613	725	132	14.41236	9.24	−0.873485	1	other	S
BnLEA14	BnaA08g01460D	LEA_4	A8	1181931	1182535	605	103	12.08013	11.75	−1.482524	1	SP	S
BnLEA15	BnaC03g44340D	Dehydrin	C3	29465468	29466562	1095	95	10.51938	6.7	−1.892632	0		S
BnLEA16	BnaA02g15750D	LEA_4	A2	9189066	9190802	1736	442	48.81765	9.13	−0.399774	2	other	C
BnLEA17	BnaC02g21020D	LEA_4	C2	17646375	17648170	1795	460	50.69263	8.95	−0.407826	1	other	C
BnLEA18	BnaA02g36030D	Dehydrin	A2-R	666514	667522	1009	194	21.72529	5.61	−1.351546	1	other	S
BnLEA19	BnaA07g32420D	Dehydrin	A7	22475699	22476668	970	195	22.02149	5.51	−1.470769	1	SP	S
BnLEA20	BnaA07g21490D	Dehydrin	A7	16630156	16631174	1019	194	21.75535	5.47	−1.308247	1	other	O
BnLEA21	BnaC06g21970D	Dehydrin	C6	24121429	24122509	1081	183	20.58607	5.41	−1.327869	1	other	O
BnLEA22	BnaC06g36880D	Dehydrin	C6	35172305	35173301	997	199	22.43891	5.44	−1.470352	1	SP	S
BnLEA23	BnaC07g22530D	LEA_4	C7	28947408	28949034	1627	201	21.88081	5.5	−0.417413	1	other	O
BnLEA24	BnaC02g35130D	LEA_4	C2	37914683	37915913	1231	192	20.38898	8.57	−0.694792	1	other	O
BnLEA25	BnaA08g15290D	LEA_4	A8	12745496	12747188	1693	487	52.4039	5.75	−0.931417	1	other	
BnLEA26	BnaC07g03410D	LEA_4	C7	4585045	4586676	1632	480	52.5935	6.28	−0.920417	1	other	
BnLEA27	BnaC08g34610D	LEA_6	C8	32717932	32718420	489	82	8.50826	4.58	−0.969512	0	other	O
BnLEA28	BnaA09g42180D	LEA_6	A9	29346244	29346775	532	82	8.43311	4.72	−1.054878	0	other	S
BnLEA29	BnaA04g19670D	LEA_6	A4	15962219	15962678	459	72	7.6273	5.21	−1.173611	0	SP	S
BnLEA30	BnaC03g18750D	LEA_6	C3	9610417	9610873	456	71	7.52619	5.21	−1.180282	0	SP	S
BnLEA31	BnaC04g44060D	LEA_6	C4	44255276	44255629	354	72	7.62927	5.21	−1.211111	0	SP	S
BnLEA32	BnaC04g09820D	LEA_1	C4	7426352	7426686	335	98	10.64785	8.9	−1.102041	0	other	O
BnLEA33	BnaA05g08680D	LEA_1	A5	4813153	4813480	328	98	10.64785	8.9	−1.102041	0	other	O
BnLEA34	BnaA04g05010D	LEA_4	A4	3676235	3677724	1490	451	48.5404	5.2	−1.152106	2	other	O
BnLEA35	BnaA05g07720D	LEA_4	A5	4193502	4194949	1448	410	45.0119	5.55	−1.033902	2	SP	S
BnLEA36	BnaC04g08680D	LEA_4	C4	6503952	6505333	1382	388	42.95568	5.42	−1.039691	2	SP	S
BnLEA37	BnaCnng32920D	LEA_4	Un-R	31247747	31249402	1656	452	48.75274	5.28	−1.163274	2	other	
BnLEA38	BnaA03g18930D	LEA_5	A3	8931994	8932678	685	88	9.63038	5.88	−1.659091	1	other	S
BnLEA39	BnaA04g22520D	LEA_5	A4	17516524	17517040	517	114	12.5603	9.58	−1.004386	1	SP	S
BnLEA40	BnaA05g34530D	LEA_5	A5-R	192195	192930	736	84	9.21889	6.74	−1.677381	1	other	S
BnLEA41	BnaC03g22490D	LEA_5	C3	12409877	12410478	602	88	9.58735	5.88	−1.598864	1	other	S
BnLEA42	BnaCnng27950D	LEA_5	Un-R	26525490	26526251	762	84	9.17983	5.69	−1.597619	1	other	S
BnLEA43	BnaC03g23770D	LEA_4	C3	13254482	13255809	1328	142	14.83843	5.29	−0.544366	1	other	
BnLEA44	BnaC04g48420D	LEA_4	C4	47021142	47023669	2528	638	68.65427	6	−1.03605	2	other	O
BnLEA45	BnaC03g23780D	LEA_4	C3	13259116	13259900	785	174	18.67633	8.02	−1.181609	1	other	O
BnLEA46	BnaA03g20620D	LEA_2	A3	9781842	9782601	760	180	20.01815	4.71	−0.12	0		C
BnLEA47	BnaC03g24650D	LEA_2	C3	13839768	13841416	1649	321	35.38161	4.72	−0.195016	0	other	O
BnLEA48	BnaC04g49420D	LEA_2	C4	47518499	47520741	2243	188	21.01336	4.81	−0.154787	0	SP	S
BnLEA49	BnaA03g21280D	LEA_2	A3	10108303	10109397	1095	161	17.45194	4.71	−0.015528	1	other	C
BnLEA50	BnaA05g01660D	LEA_2	A5	949251	950675	1425	166	17.72534	4.81	0.0927711	1	other	
BnLEA51	BnaC03g25640D	LEA_2	C3	14384786	14385909	1124	161	17.45194	4.71	−0.015528	1	other	C
BnLEA52	BnaC04g00870D	LEA_2	C4	759845	760933	1089	166	17.75236	4.81	0.076506	1	other	C
BnLEA53	BnaC04g50740D	LEA_2	C4	48258585	48259654	1070	164	17.91156	4.59	−0.041463	1	other	C
BnLEA54	BnaA01g28600D	LEA_4	A1	19885286	19886322	1037	226	24.59855	9.02	−1.475221	1	SP	S
BnLEA55	BnaA05g23860D	LEA_4	A5	17980857	17981901	1045	229	24.82188	8.81	−1.421397	1	SP	S
BnLEA56	BnaC03g39230D	LEA_4	C3	24204689	24205706	1018	196	21.04774	8.58	−1.345918	1	SP	S
BnLEA57	BnaC05g37670D	LEA_4	C5	36601942	36603206	1265	229	24.78186	8.83	−1.398253	1	SP	S
BnLEA58	BnaA03g34560D	LEA_4	A3	16813606	16814466	861	286	31.4549	6.11	−1.104545	0	other	O
BnLEA59	BnaC03g40050D	LEA_4	C3	25026958	25027818	861	286	31.56984	5.79	−1.122028	0	other	O
BnLEA60	BnaC05g35990D	LEA_4	C5	35179665	35180572	908	296	32.2595	5.56	−1.106757	0	other	O
BnLEA61	BnaAnng35040D	LEA_4	Un-R	39836438	39837624	1187	297	32.33053	5.54	−1.118855	0	other	O
BnLEA62	BnaA03g36660D	SMP	A3	11747905	11748957	1052	239	25.44855	5.97	−0.375314	2	SP	S
BnLEA63	BnaA05g17150D	SMP	A5	11902363	11903533	1050	262	26.68053	4.71	−0.21374	2		S
BnLEA64	BnaC03g42830D	SMP	C3	27572917	27573846	930	254	26.29029	5.01	−0.314961	2	SP	S
BnLEA65	BnaC05g29980D	SMP	C5	28994596	28995799	1204	262	26.68247	4.76	−0.260687	2	SP	S
BnLEA66	BnaC05g29930D	SMP	C5	28841503	28842692	1189	262	26.8617	4.71	−0.275191	2	SP	S
BnLEA67	BnaA07g12750D	Dehydrin	A7	12584077	12583156	921	75	8.79009	9.4	−0.618667	1	other	O
BnLEA68	BnaA09g43150D	Dehydrin	A9	29958956	29960700	1745	183	19.17891	6.67	−1.020765	1	SP	O
BnLEA69	BnaA09g31640D	Dehydrin	A9	23599068	23600339	1272	136	14.04947	9.36	−0.908824	1	SP	S
BnLEA70	BnaC08g35660D	Dehydrin	C8	33390683	33391896	1213	180	19.09571	6.38	−1.095556	1	SP	O
BnLEA71	BnaC08g22390D	Dehydrin	C8	25080735	25082027	1293	134	13.87329	9.19	−0.823134	1	SP	S
BnLEA72	BnaA01g19290D	LEA_5	A1	10830022	10831119	1098	153	16.8394	6.2	−1.547712	1	other	S
BnLEA73	BnaC01g23250D	LEA_5	C1	16904412	16905363	952	152	16.65512	6.02	−1.559868	1	other	S
BnLEA74	BnaA04g04540D	LEA_3	A4	3347814	3348294	481	122	14.08403	8.58	−0.584426	1	SP	C
BnLEA75	BnaC04g27000D	LEA_3	C4	28240158	28240638	481	122	14.07704	6.73	−0.512295	1	SP	C
BnLEA76	BnaA02g36510D	LEA_3	A2-R	996220	997101	882	93	10.09248	9.99	−0.464516	1	SP	C
BnLEA77	BnaA03g26220D	LEA_3	A3	12840782	12841536	755	94	10.09553	9.85	−0.348936	1	SP	C
BnLEA78	BnaA09g00750D	LEA_3	A9	476938	477869	932	94	10.0154	9.82	−0.323404	1	SP	C
BnLEA79	BnaC02g28140D	LEA_3	C2	26444383	26445280	898	80	8.88522	9.75	−0.465	1	SP	
BnLEA80	BnaC03g73200D	LEA_3	C3-R	1368770	1369504	735	94	10.06855	9.99	−0.330851	1	SP	C
BnLEA81	BnaCnng19220D	LEA_3	Un-R	17903980	17904866	887	94	9.98334	9.82	−0.298936	1	SP	
BnLEA82	BnaA01g18270D	LEA_3	A1	9793769	9794619	851	99	10.58201	9.72	−0.4	1	SP	O
BnLEA83	BnaC01g22200D	LEA_3	C1	15640472	15641445	974	99	10.71217	9.81	−0.365657	1		O
BnLEA84	BnaAnng29120D	LEA_3	Un-R	38806950	38807364	414	57	14.04968	5.38	0.8298851	1	SP	O
BnLEA85	BnaA01g10880D	LEA_4	A1	5434921	5436380	1460	253	27.8239	8.55	−1.208696	2	SP	S
BnLEA86	BnaA08g09930D	LEA_4	A8	9347111	9348357	1247	241	26.40417	5.86	−1.130705	1	SP	C
BnLEA87	BnaC03g64470D	LEA_4	C3	53827988	53829257	1270	241	26.46122	5.56	−1.134855	1	SP	C
BnLEA88	BnaCnng20790D	Dehydrin	Un-R	31264938	31265920	983	245	27.81099	6.75	−1.601633	1		O
BnLEA89	BnaA01g05360D	Dehydrin	A1	2510784	2511384	601	149	15.98646	5.5	−0.9	1	SP	O
BnLEA90	BnaC01g00220D	Dehydrin	C1	66017	66890	874	149	15.97744	5.89	−0.90604	1	other	O
BnLEA91	BnaA10g24180D	LEA_1	A10	15823426	15824289	864	159	16.18083	8.93	−0.81761	1	SP	C
BnLEA92	BnaA03g55670D	LEA_1	A3-R	368052	368922	871	159	16.22993	9.22	−0.772327	1	other	C
BnLEA93	BnaC09g48810D	LEA_1	C9	47495664	47496551	888	159	16.26397	9.3	−0.81761	1	other	
BnLEA94	BnaCnng44320D	LEA_1	Un-R	43384275	43384918	644	159	16.20089	9.43	−0.784906	1	other	C
BnLEA95	BnaA06g29020D	SMP	A6	19835134	19835986	853	191	19.39032	4.78	−0.508901	2		
BnLEA96	BnaA09g03580D	SMP	A9	1813221	1814143	923	191	19.58674	4.99	−0.424607	2	SP	S
BnLEA97	BnaC02g39520D	SMP	C2	42399243	42400063	821	184	18.88701	5.08	−0.496739	2		S
BnLEA98	BnaC07g27650D	SMP	C7	33089319	33090177	859	191	19.40434	4.76	−0.496335	2		
BnLEA99	BnaC09g02960D	SMP	C9	1691274	1691921	648	166	17.89085	5.09	−0.630723	1	other	O
BnLEA100	BnaAnng11440D	SMP	Un-R	12468275	12469079	805	183	18.63766	4.94	−0.478689	2	SP	S
BnLEA101	BnaA02g10350D	SMP	A2	5299569	5300820	1251	173	18.40747	5.7	−0.372832	1		
BnLEA102	BnaA03g12360D	SMP	A3	5635572	5636420	848	177	18.69181	5.43	−0.350847	1		C
BnLEA103	BnaC02g14440D	SMP	C2	9880876	9882004	1128	173	18.48357	5.76	−0.368786	2		
BnLEA104	BnaA02g34800D	Dehydrin	A2	24758147	24759109	963	190	18.62394	7.14	−1.022632	1	SP	S
BnLEA105	BnaA09g07980D	Dehydrin	A9	3870715	3871730	1016	179	17.93829	7.14	−1.111173	1	SP	S
BnLEA106	BnaC02g45160D	Dehydrin	C2-R	902115	903109	995	178	17.65097	7.97	−1.074157	2	SP	S
BnLEA107	BnaC09g08130D	Dehydrin	C9	5162376	5163397	1022	179	17.89423	7.14	−1.109497	1	SP	S
BnLEA108	BnaCnng27850D	Dehydrin	Un-R	26402274	26403289	1016	179	17.93829	7.14	−1.111173	1	SP	S

**Table 2:** Synonymous (Ks) and nonsynonymous (Ka) nucleotide substitution rates for *Arabidopsis thaliana* and *B. napus* LEA protein coding loci.

*A. thaliana*ID	*B. napus* gene	*B. napus* ID	LEA family	Ka	Ks	Ka/Ks
one copy loci						
At1g52690	BnLEA14	BnaA08g01460D	LEA_4	0.7067	1.2346	0.5724
At1g54410	BnLEA15	BnaC03g44340D	Dehydrin	0.0548	0.3803	0.1442
At2g03740	BnLEA23	BnaC07g22530D	LEA_4	0.1925	0.3826	0.503
At2g03850	BnLEA24	BnaC02g35130D	LEA_4	0.1862	0.4153	0.4484
At2g42540	BnLEA43	BnaC03g23770D	LEA_4	0.1632	0.475	0.3437
At3g22500	BnLEA66	BnaC05g29930D	SMP	0.0709	0.6455	0.1098
At4g38410	BnLEA88	BnaCnng20790D	Dehydrin	0.2608	0.7055	0.3697
At5g53270	BnLEA103	BnaC02g14440D	SMP	0.2038	0.688	0.2962
two-copy loci						
At1g01470	BnLEA1	BnaAnng17910D	LEA_2	0.0588	0.5582	0.1053
	BnLEA2	BnaCnng23520D	LEA_2	0.0683	0.5566	0.1227
At1g02820	BnLEA3	BnaA10g01410D	LEA_3	0.1498	0.2328	0.6434
	BnLEA4	BnaC05g01450D	LEA_3	0.1588	0.3306	0.4802
At1g03120	BnLEA5	BnaA10g01720D	SMP	0.147	0.4696	0.3131
	BnLEA6	BnaC05g01750D	SMP	0.1408	0.4427	0.318
At1g20440	BnLEA7	BnaC07g15380D	Dehydrin	0.2334	0.6126	0.381
	BnLEA8	BnaAnng29030D	Dehydrin	0.3024	0.7686	0.3935
At1g20450	BnLEA9	BnaA07g11450D	Dehydrin	0.1357	0.6826	0.1988
	BnLEA10	BnaC05g15780D	Dehydrin	0.1605	0.7191	0.2232
At1g72100	BnLEA16	BnaA02g15750D	LEA_4	0.1119	0.6431	0.1739
	BnLEA17	BnaC02g21020D	LEA_4	0.11	0.6099	0.1803
At2g18340	BnLEA25	BnaA08g15290D	LEA_4	0.3728	3.4912	0.1068
	BnLEA26	BnaC07g03410D	LEA_4	0.2374	1.2281	0.1933
At2g23120	BnLEA27	BnaC08g34610D	LEA_6	0.2586	0.6854	0.3772
	BnLEA28	BnaA09g42180D	LEA_6	0.2666	0.7245	0.368
At2g35300	BnLEA32	BnaC04g09820D	LEA_1	0.0817	0.4368	0.1871
	BnLEA33	BnaA05g08680D	LEA_1	0.0795	0.4442	0.179
At2g42560	BnLEA44	BnaC04g48420D	LEA_4	0.369	1.0387	0.3553
	BnLEA45	BnaC03g23780D	LEA_4	0.4797	0.9653	0.4969
At3g51810	BnLEA72	BnaA01g19290D	LEA_5	0.0435	0.3313	0.1312
	BnLEA73	BnaC01g23250D	LEA_5	0.0316	0.3791	0.0834
At3g53770	BnLEA74	BnaA04g04540D	LEA_3	0.1791	0.617	0.2903
	BnLEA75	BnaC04g27000D	LEA_3	0.1745	0.5878	0.297
At4g39130	BnLEA89	BnaA01g05360D	Dehydrin	0.174	0.5601	0.3107
	BnLEA90	BnaC01g00220D	Dehydrin	0.177	0.4855	0.3647
At5g53260	BnLEA101	BnaA02g10350D	SMP	0.1625	0.6361	0.2555
	BnLEA102	BnaA03g12360D	SMP	0.1581	0.6627	0.2386
three-copy loci						
At1g32560	BnLEA11	BnaA09g24240D	LEA_1	0.1466	0.6935	0.2114
	BnLEA12	BnaC05g24660D	LEA_1	0.1548	0.6626	0.2337
	BnLEA13	BnaC05g24760D	LEA_1	0.1548	0.6626	0.2337
At2g33690	BnLEA29	BnaA04g19670D	LEA_6	0.1035	0.4756	0.2177
	BnLEA30	BnaC03g18750D	LEA_6	0.1012	0.3467	0.292
	BnLEA31	BnaC04g44060D	LEA_6	0.1083	0.2579	0.4199
At2g44060	BnLEA46	BnaA03g20620D	LEA_2	0.0282	0.744	0.0379
	BnLEA47	BnaC03g24650D	LEA_2	0.0308	0.7655	0.0403
	BnLEA48	BnaC04g49420D	LEA_2	0.0347	1.9341	0.0179
At4g15910	BnLEA82	BnaA01g18270D	LEA_3	0.0722	0.4218	0.1712
	BnLEA83	BnaC01g22200D	LEA_3	0.0981	0.4633	0.2117
	BnLEA84	BnaAnng29120D	LEA_3	0.2112	0.5351	0.3947
At4g21020	BnLEA85	BnaA01g10880D	LEA_4	0.1763	0.853	0.2067
	BnLEA86	BnaA08g09930D	LEA_4	0.1087	0.684	0.1576
	BnLEA87	BnaC03g64470D	LEA_4	0.1155	0.6272	0.1841
four-copy loci						
At2g36640	BnLEA34	BnaA04g05010D	LEA_4	0.2487	2.1063	0.1181
	BnLEA35	BnaA05g07720D	LEA_4	0.1621	0.7767	0.2088
	BnLEA36	BnaC04g08680D	LEA_4	0.1667	0.813	0.205
	BnLEA37	BnaCnng32920D	LEA_4	0.2532	2.0584	0.123
At3g15670	BnLEA54	BnaA01g28600D	LEA_4	0.069	0.5519	0.1251
	BnLEA55	BnaA05g23860D	LEA_4	0.057	0.4961	0.1148
	BnLEA56	BnaC03g39230D	LEA_4	0.0911	0.6166	0.1477
	BnLEA57	BnaC05g37670D	LEA_4	0.0691	0.4481	0.1542
At3g17520	BnLEA58	BnaA03g34560D	LEA_4	0.1528	0.6935	0.2203
	BnLEA59	BnaC03g40050D	LEA_4	0.1613	0.7164	0.2252
	BnLEA60	BnaC05g35990D	LEA_4	0.1158	0.6424	0.1802
	BnLEA61	BnaAnng35040D	LEA_4	0.1353	0.6593	0.2052
At3g22490	BnLEA62	BnaA03g36660D	SMP	0.2646	0.8118	0.3259
	BnLEA63	BnaA05g17150D	SMP	0.0539	0.5809	0.0928
	BnLEA64	BnaC03g42830D	SMP	0.1226	0.5713	0.2146
	BnLEA65	BnaC05g29980D	SMP	0.0518	0.6093	0.085
At5g06760	BnLEA91	BnaA10g24180D	LEA_1	0.0781	0.4787	0.1632
	BnLEA92	BnaA03g55670D	LEA_1	0.0814	0.3857	0.2111
	BnLEA93	BnaC09g48810D	LEA_1	0.086	0.4548	0.1891
	BnLEA94	BnaCnng44320D	LEA_1	0.0735	0.3518	0.2089
five-copy loci						
At1g76180	BnLEA18	BnaA02g36030D	Dehydrin	0.1486	0.8539	0.1741
	BnLEA19	BnaA07g32420D	Dehydrin	0.1058	0.6962	0.152
	BnLEA20	BnaA07g21490D	Dehydrin	0.1351	0.5254	0.2571
	BnLEA21	BnaC06g21970D	Dehydrin	0.2061	0.5737	0.3592
	BnLEA22	BnaC06g36880D	Dehydrin	0.1059	0.7429	0.1425
At2g40170	BnLEA38	BnaA03g18930D	LEA_5	0.1214	0.5316	0.2283
	BnLEA39	BnaA04g22520D	LEA_5	0.1706	0.5374	0.3174
	BnLEA40	BnaA05g34530D	LEA_5	0.0701	0.4639	0.1512
	BnLEA41	BnaC03g22490D	LEA_5	0.1237	0.5491	0.2253
	BnLEA42	BnaCnng27950D	LEA_5	0.0699	0.4718	0.1481
At2g46140	BnLEA49	BnaA03g21280D	LEA_2	0.0615	0.2822	0.218
	BnLEA50	BnaA05g01660D	LEA_2	0.051	0.2381	0.214
	BnLEA51	BnaC03g25640D	LEA_2	0.0615	0.3077	0.2
	BnLEA52	BnaC04g00870D	LEA_2	0.0539	0.2264	0.2381
	BnLEA53	BnaC04g50740D	LEA_2	0.071	0.3512	0.2022
At3g50980	BnLEA67	BnaA07g12750D	Dehydrin	0.8386	1.8538	0.4524
	BnLEA68	BnaA09g43150D	Dehydrin	0.6922	2.0064	0.345
	BnLEA69	BnaA09g31640D	Dehydrin	0.1638	0.5798	0.2825
	BnLEA70	BnaC08g35660D	Dehydrin	0.7028	1.8344	0.3831
	BnLEA71	BnaC08g22390D	Dehydrin	0.1687	0.5994	0.2814
At5g66400	BnLEA104	BnaA02g34800D	Dehydrin	0.1177	0.7369	0.1597
	BnLEA105	BnaA09g07980D	Dehydrin	0.1106	0.6754	0.1638
	BnLEA106	BnaC02g45160D	Dehydrin	0.106	0.7618	0.1391
	BnLEA107	BnaC09g08130D	Dehydrin	0.1088	0.685	0.1588
	BnLEA108	BnaCnng27850D	Dehydrin	0.1106	0.6754	0.1638
six-copy loci						
At4g02380	BnLEA76	BnaA02g36510D	LEA_3	0.1184	0.3385	0.3497
	BnLEA77	BnaA03g26220D	LEA_3	0.0637	0.1885	0.3382
	BnLEA78	BnaA09g00750D	LEA_3	0.1519	0.3104	0.4894
	BnLEA79	BnaC02g28140D	LEA_3	0.1249	0.3385	0.369
	BnLEA80	BnaC03g73200D	LEA_3	0.0519	0.1865	0.2784
	BnLEA81	BnaCnng19220D	LEA_3	0.1592	0.3073	0.518
At5g27980	BnLEA95	BnaA06g29020D	SMP	0.1238	0.5246	0.236
	BnLEA96	BnaA09g03580D	SMP	0.1225	0.6594	0.1858
	BnLEA97	BnaC02g39520D	SMP	0.1027	0.5201	0.1974
	BnLEA98	BnaC07g27650D	SMP	0.1172	0.5252	0.2231
	BnLEA99	BnaC09g02960D	SMP	0.4517	0.8193	0.5513
	BnLEA100	BnaAnng11440D	SMP	0.1217	0.6119	0.1988
