# Supplementary material for: Genome-wide identification, structural analysis and new insights into late embryogenesis abundant (LEA) gene family formation pattern in Brassica napus
Source: Sci Rep. 2016 Apr 13;6:24265. doi: 10.1038/srep24265 (PMC4829847; doi:10.1038/srep24265)
Supplement: Supplementary Information [file srep24265-s1.pdf]

Genome-wide identification, structural analysis and new insights into late embryogenesis abundant (*LEA*) gene family formation pattern in *Brassica napus*

Yu Liang[1, 2], Ziyi Xiong[1], Jianxiao Zheng[1], Dongyang Xu[1], Zeyang Zhu[1], Jun Xiang[2], Jianping Gan[2], Nadia Raboanatahiry [1], Yongtai Yin[1], Maoteng Li[1, 2]*

[1] Department of Biotechnology, College of Life Science and Technology, Huazhong University of Science and Technology, Wuhan, China, 430074.

[2] Hubei Collaborative Innovation Center for the Characteristic Resources Exploitation of Dabie Mountains, Huanggang Normal University, Huanggang 438000, China

*correspondence author: E-mail: limaoteng426@mail.hust.edu.cn

Supplementary Information

Figure S1

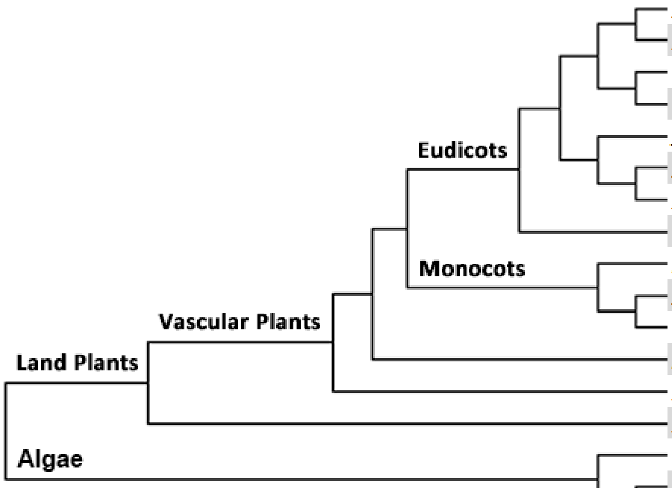

	LEA_1	LEA_2	LEA_3	LEA_4	LEA_5	LEA_6	Dehydrin	SMP	SUM
Brassica napus	9	10	13	25	7	5	23	16	108
Populus trichocarpa	3	4	5	26	1	2	10	2	53
Glycine max	1	7	1	6	5	1	9	4	34
Vitis vinifera	2	2	3	18	1	2	3	2	33
Arabidopsis thaliana	3	3	4	18	2	3	10	6	49
Brassica oleracea	6	5	4	14	2	3	10	5	49
Brassica rapa	5	5	8	22	3	3	13	7	66
Gossypium hirsutum	2	3	1	2	3	1	7	6	25
Setaria italica	0	5	0	2	1	0	8	1	17
Zea mays	4	2	8	2	0	1	7	4	28
Oryza sativa	4	5	6	9	2	2	8	9	45
Selaginella moellendorffii	2	10	5	16	6	0	0	8	47
Pinus taeda	3	1	6	16	3	1	10	3	43
Physcomitrella patens	1	4	1	27	4	0	2	3	42
Thalassiosira pseudonana	0	1	0	0	0	0	0	1	2
Volvox carteri	0	0	0	3	2	0	0	0	5
Chlamydomonas reinhardtii	0	0	0	2	1	0	0	0	3
**Total**	45	67	65	208	43	24	120	77	649

Supplementary Information

Figure S2

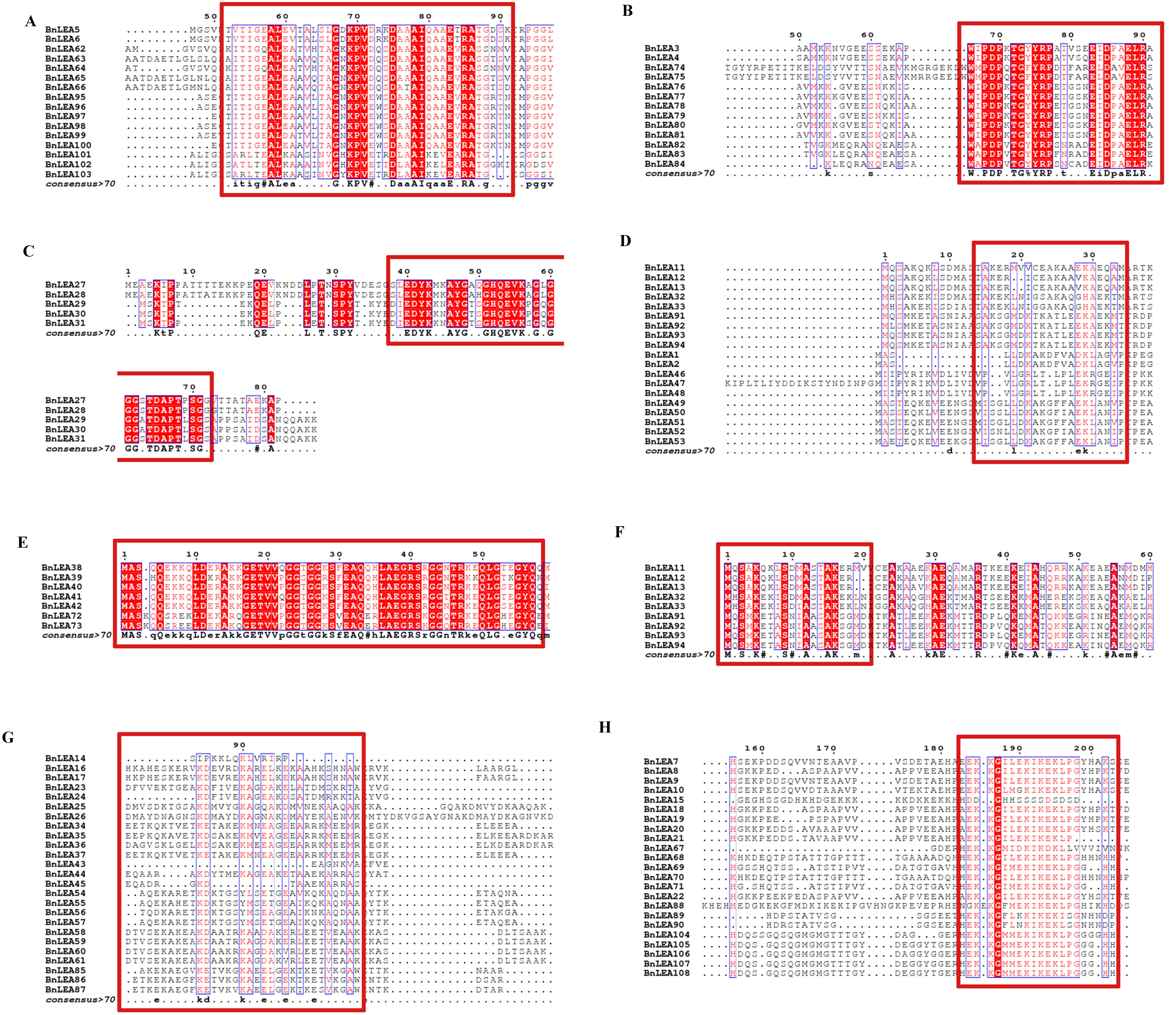
A
BnLEA5 BnLEA6 BnLEA62 BnLEA63 BnLEA64 BnLEA65 BnLEA66 BnLEA95 BnLEA96 BnLEA97 BnLEA98 BnLEA99 BnLEA100 BnLEA101 BnLEA102 BnLEA103
consensus>70
B
BnLEA3 BnLEA4 BnLEA74 BnLEA75 BnLEA76 BnLEA77 BnLEA78 BnLEA79 BnLEA80 BnLEA81 BnLEA82 BnLEA83 BnLEA84
consensus>70
C
BnLEA27 BnLEA28 BnLEA29 BnLEA30 BnLEA31
consensus>70
D
BnLEA11 BnLEA12 BnLEA13 BnLEA32 BnLEA33 BnLEA91 BnLEA92 BnLEA93 BnLEA94 BnLEA1 BnLEA2 BnLEA46 BnLEA47 BnLEA48 BnLEA49 BnLEA50 BnLEA51 BnLEA52 BnLEA53
consensus>70
E
BnLEA38 BnLEA39 BnLEA40 BnLEA41 BnLEA42 BnLEA72 BnLEA73
consensus>70
F
BnLEA11 BnLEA12 BnLEA13 BnLEA32 BnLEA33 BnLEA91 BnLEA92 BnLEA93 BnLEA94
consensus>70
G
BnLEA14 BnLEA16 BnLEA17 BnLEA23 BnLEA24 BnLEA25 BnLEA26 BnLEA34 BnLEA35 BnLEA36 BnLEA37 BnLEA43 BnLEA44 BnLEA45 BnLEA54 BnLEA55 BnLEA56 BnLEA57 BnLEA58 BnLEA59 BnLEA60 BnLEA61 BnLEA85 BnLEA86 BnLEA87
consensus>70
H
BnLEA7 BnLEA8 BnLEA9 BnLEA10 BnLEA15 BnLEA18 BnLEA19 BnLEA20 BnLEA21 BnLEA67 BnLEA68 BnLEA69 BnLEA70 BnLEA71 BnLEA22 BnLEA88 BnLEA89 BnLEA90 BnLEA104 BnLEA105 BnLEA106 BnLEA107 BnLEA108

Supplementary Information

Figure S3

```
               1        10        20        30        40        50        60
BnLEA27        MEAEKIPPATTTTEKKPEQEVKNDDLPTNSPYVDESGSLEDYKMKAYGAQGHQEVKAGLG
BnLEA28        MEAEKTPPATTATEKKPEQEVKNDDLPTNSPYVDESGSLEDYKMKAYGAHGHQEVKAGLG
BnLEA29        ..MSKTPT........EKQELP...LET.SPYT.KYEDIEDYKKNAYGTSGHQEVKPGQG
BnLEA30        ..MSKTP.........EKQELP...LET.SPYT.KYEDIEDYKKNAYGTSGHQEVKPGQG
BnLEA31        ..MSKTPP........EKQELP...LET.SPYT.KYEDIEDYKKNAYGTSGHQEVKSGQG
consensus>70   ....KtP...........QE.....L.T.SPY.......EDYK..AYG..GHQEVK.G.G

                        70        80
BnLEA27        GGSTDAPTPSGGVTTATAEKAP.....
BnLEA28        GGSTDAPTPSGGGTTATAEKAP.....
BnLEA29        GGATDAPTLSGSAPPSAIDSANQQAKK
BnLEA30        GGATDAPTLSGSAPPSAIDSANQQAKK
BnLEA31        GGSTDAPTLSGSAPPSAIDSANQQAKK
consensus>70   GG.TDAPT.SG.......#.A......
```

BnLEA proteins of LEA_6 family

```
BnLEA14       ............................................................
BnLEA16       .........MTNLLALCLVFSTLFAAEVWSPSPAVTTQQTVVSEDDVIVKDGHRVVVVEY
BnLEA17       .........MTNLLALCLVFSTLFAAEVWSPSPSVTTQQTVVSEDDVIVKDGHRVVVVEY
BnLEA23       ............MATLISG.AVLSGLGSTFLIG...........................
BnLEA24       ............MSMSISG.AMLSGVGSSLLING.........................S
BnLEA25       .........MAIIMEKRSLMMMVFMLMVILAWQNECHGWEAAEDIVRNESEHSKNAAGTV
BnLEA26       ............MMERRRTALALLLVVVVLTWQKGTT............AKDARSAAEMA
BnLEA34       MASGQREAEKEAKAERAEAAARLAADDLRDVNEGG....VTYKVTEKTTT.EHPSAVVEE
BnLEA35       MAS.....EKQQKTERAEVAARLAAEDLHDINKHHRDNVTMYKVTERTVE..HPP....E
BnLEA36       MTS.....EKQLKAERAEVAARLAAEDLHDINKHHRDNVTMYKVTERTVE..HPR....E
BnLEA37       MASGQREAEKAAKAERAEAAARLAADDLRDVNEGG....VTYKVTEKTTTTEHPSAVVEE
BnLEA43       ............MAMSLSGSAVLSGIGSSFSSGA.........................A
BnLEA44       ............MASEQARRENVVKEREVQVEKDRVP..........KMTSHFESIAEKG
BnLEA45       ............MASEQARRENVVKQREVKVEKD........................KG
BnLEA54       ............................................................
BnLEA55       ............................................................
BnLEA56       ............................................................
BnLEA57       ............................................................
BnLEA58       ............MGLGRRI....IVSLVLMAIVTMCC.................VKATIA
BnLEA59       ............MGLGKRV....IVSLVLMAIVTNCC.................VKATIA
BnLEA60       ............MGLGKRVYGLCIVSLVLMAVVT.....................MATIE
BnLEA61       ............MGLGKRVYGLCIVSLVLMAVVT.....................MATIE
BnLEA85       ..........MKMAPMQLTRATLFG..LSKALPIARSPATLTAS.TRKVSRVCFASSVSH
BnLEA86       ............MASMQLARATLFS..LSKAFPIVRSPLTLAASSTRKVSRVCFASSVSH
BnLEA87       ............MASMQLTRATLFS..LSKAFPIVRSPLTLAASSTRKVSRVCFASSVSR
consensus>70  ............................................................
```

```
                                                 1       10
BnLEA14       ....................................MTSHQEKSYKAGETRGKT......
BnLEA16       DRDGKTN..............TRVSISPPSADEGEQKQEVEKETTLFRHAKEKAKETASY
BnLEA17       DRDGKTN..............TRVSISPPSADEGEQKQEVEKETTLFRHAKEKAKETASY
BnLEA23       RRSG...................................AGRGEVRFGWKNVIIAP....
BnLEA24       KRSGG..................................VGGGSMSVGRKNATITP....
BnLEA25       TKMAAKATR..........DANDKTASWTGWVSDKISTGLGSKKEEAKEAAESAKNYAYD
BnLEA26       KKMAT.................ETVSWAGWVSDRITTGLGIKKKEPESAAQRTKNYAYK
BnLEA34       TERPGIIGSVMKAVQG....TKDAVIGKSHDAAESTKEGAEVASGKAGEVKDATGEKAGE
BnLEA35       QERPGVIGSVFRAVQGTYEHARDAVVGKSHDVAESTREGAQIASEKAAGAKDATLEKA..
BnLEA36       QERPGVIGSVFRAVTENYEHARDAVVGKSHDVAESTREGAQIASEKAAGAKDATLEKA..
BnLEA37       TERPGIIGSVMKAVQG....TKDAVIGKSHDAAESTKEGSEVASGKAGEVKDATAEKAGE
BnLEA43       KQSG.....................................VGAVGFGRKTEFVVV....
BnLEA44       KDSDTQ...................RQQETTTHFVSLSDKGNEGEGETKMKMTK....
BnLEA45       RDSGVH...................VTHAAT.......ERGSHGGGEAAAVFGQE...
BnLEA54       .....................................MASNQQSYKAGETRGKTQEKTG.
BnLEA55       .....................................MASNQQSYKAGETRGKTQEKTG.
BnLEA56       .....................................MASNQQSYKAGETRGKTQEKTG.
BnLEA57       .....................................MASNQQSYKAGETRGKTQEKTG.
BnLEA58       EEAAK..................GKSWTDWAKEKI....GLKHEEK.IPTTHTTT....
BnLEA59       EDVAK..................SKSWTDWAKEKI....GLKHEEN.IPTTHTTS....
BnLEA60       EEAAK..................DESWTDWAKEKI....GLKHEEHNVPTTHTTT....
BnLEA61       EEAAK..................DESWTDWAKEKI....GLKHEEHNVPTTHTTT....
BnLEA85       S.EGRD.....................PVGNARDSKADLDYGSKKWREDTGEN......
BnLEA86       S.EGRD.....................PVENARDSRADVPYGSKKWRENTEEN......
BnLEA87       S.EGRD.....................PVENARDSRADVPYGSKKWRENTEEN......
consensus>70  ............................................................
```

```
                                 20        30                  40        50
BnLEA14       .................QAAKDKTQGAGRSTQP.........MKQLNQLKTRHLKAHKQL
BnLEA16       FPNV...GQGISQPVVTEEARDHHATAGEVICDAFGKCRQKIASVVGRAKDRASDRVDDV
BnLEA17       FPNV...GQGISQPVVTEEARDHHATAGEVICDAFGKCRQKISSVVGRAKDRASDTVDDV
BnLEA23       ........QRKKSWVMAAVKGDD...GNSKLDPK..........WLDDASEKASEYVKEK
BnLEA24       ........QRNKSWALAAVKGD....GKSKNDPK..........WLDDASQKAGEFVKDM
BnLEA25       NAG....SAYDNAGYAKDFVSDKAGSAYDSAQNAKGY....AYEKATDAKDMVYDKAGQA
BnLEA26       AAQYIKDSAYGMAGDAKDMAYEKASNAKEMASEKTGYVKDMAYEQAGHAKDYAYDKAGNA
BnLEA34       VTDR...TAN.KTKETADYTAEKAKEAKDKTAEQVGEYKDYTAEKAKEAKDRTAEKTKES
BnLEA35       .............KDTADYTAEKAREVKDKTAEKMGEYKDYTVDKAKEAKDKTVDKMGEY
BnLEA36       .............KDTADYTAEKAREAKDKTAEKIGEYKNYTVDKAVEAKDKTAEKAKET
BnLEA37       VKDR...TAN.KTKETADYTAEKAKEAKDKTAEKVGEYKDYTAEKAKEAKDRTAEKTKET
BnLEA43       ........AQRKKSLIYADKGD....GNILDD.............LNEATKRASDYATEK
BnLEA44       ........MPHTVGKFVVHSGDKEGTGKKEEEEQERATLEDIQGFRANAQQKSMDTVRAA
BnLEA45       ........GPGRVG..VELTGE....GREELESG.......AHGFHGEKARHAQLLAAGG
BnLEA54       ..............QTMGAMRDKAEEGKNKTSQTAQTAQQKAHETTQAAKDKTSQAAQTT
BnLEA55       ..............QAMGAMRDKAEEGRDKTSQTAQTAQQKAQETAQAAKEKTSQAAQTT
BnLEA56       ..............QAMGAMRDKAEEGKNKTSQAAQTAQQKAHETAQSAKDKTSQTAQTT
BnLEA57       ..............QAMGAMRDKAEEGKDKTSQTAQIAQQKAQETAQAAKEKTSQASQTT
BnLEA58       .......TVEDNAWGTTDKAKDAKDAAKRKAEEAVGAT.......YETAKSKAGETIGSL
BnLEA59       .......TVEDNAWEATDKAKDAKDAAKRKAEEAVGAT.......YETAKSKAGETIGSL
BnLEA60       .......TVQDNVYGATDKAKDAKEAARRKAEEAVGTTKEKAEGAYETAKSKAGETIGSV
BnLEA61       .......TVQDNVYGGTDKAKDAKEAARRKAEEAVGTTKEKAEGAYETAKSKAGETIGSV
BnLEA85       ...............YAQAAKDKANEGASKAADKAYETKEKAKDTAYEAKEKAKDTAYEA
BnLEA86       ...............YAQGAKDKANEGASKAADKAYETKEQAKGTAYEAKEKAKDYAELT
BnLEA87       ...............YAQGAKDKANEGASKAADKAYETKEQAKGTAYEAKEKAKDNAELT
consensus>70  .....................#.........e...............akd...d.....
```

```
BnLEA14        NRKLKTP.....................................................
BnLEA16        GEKISD........................RDIEETVTDKA...................
BnLEA17        GEKISDTGDAAAGKAYDVK....ETVTRVARDLEETVADKA...................
BnLEA23        GSEVGHLTA...........HEGQEVLDHIQRAKHYFMEKA...................
BnLEA24        GSEVGHVTA...........QKGQEVKDHIESARNYIVEKA...................
BnLEA25        KDMVYDKSGRAKDMASDKTG....SAYDKAGQAKDLAYDKA...................
BnLEA26        KDMAYEKAGHTKDYAYDKTDNAKQGAYDKAGIAKDIAYEQA...................
BnLEA34        ADYTADK......................AKEAKDKTAEKVGEYKDYTADKAREAAEKVG
BnLEA35        KDYTVDK......................AVEARDKTAEKA...................
BnLEA36        ANYTADK......................AKEAKDKTAEK....................
BnLEA37        ADYTADK......................AKEAKDKTAEKVGEYKDYTADKAREAAEKVG
BnLEA43        TN......................EALKHGEEAKDYVVDKN...................
BnLEA44        EERYNKAKEGLSRGGQGGQ.QVEGRGRECGVHVTHGGTEKG...................
BnLEA45        EE....IRE...RKG.....QVSVRG...GRSATETVTEKG...................
BnLEA54        QQKSQET......................AQAAKDKTSQAA...................
BnLEA55        QQKAHET......................TQATKDKTSQAA...................
BnLEA56        ............................................................
BnLEA57        QQKAQET......................AQATKDKTSQAA...................
BnLEA58        KDEASQS...................YDSVGQVKDDLPHKS...................
BnLEA59        KDKAWQS...................YDSVGQVKDDLPRKS...................
BnLEA60        KDKASQS...................YDSAGQVKDDLTHKS...................
BnLEA61        KDKASQS...................YDSAGQVKDDLTHKS...................
BnLEA85        KEKTKDT..........................AYDAKEKA...................
BnLEA86        KDKVNEG..........................AYKAADKA...................
BnLEA87        KDEVNEG..........................AYKAAEKA...................
consensus>70   .d...d............................d...ek....................
```

```
                                                         60        70
BnLEA14        ...........................................RTKREATCRRLEK....
BnLEA16        ...........................................GYAKEKVGETAHDV...
BnLEA17        ...........................................GYAKEKVGETAHDA...
BnLEA23        ...........................................GVAMDMLTENAHI....
BnLEA24        ...........................................GEAMDTVAENAKK....
BnLEA25        ...........................................SQAKDMIYDTAG....S
BnLEA26        ...........................................GHAKDFAYDKAGNAKNM
BnLEA34        EYKDYTAEKAKEAKDKTAEKTKETAGYTADKAKEAKDKTAEKLGEYKDYTAEKATEG...
BnLEA35        .....................KETANYTAD...........KAGEYKDYTVEKAAEG...
BnLEA36        ..........................................MGEYKDYTVEKAIEA...
BnLEA37        EYKDYTAEKAKEAKDKTAEKTKETAGYTADKAKEAKDKTPEKLGEYKDYTAEKATEG...
BnLEA43        ...........................................VEDKDTAVDEAQK....
BnLEA44        ...........................................GSVQDTASEKTQR....
BnLEA45        ...........................................RQDKESVGKDAQR....
BnLEA54        .........................................QTTQQKAHETTQSAKE...
BnLEA55        .........................................QTTQQKAHETTQAAKD...
BnLEA56        ....................................................QSAKD...
BnLEA57        .........................................QTTQQKAHETTQAAKD...
BnLEA58        ...........................................KQVKDSFSGDNS.....
BnLEA59        ...........................................KQVKDSFSGDNS.....
BnLEA60        ...........................................KQVKDSLSGDNS.....
BnLEA61        ...........................................KKVKDSLSGDNS.....
BnLEA85        ...........................................KEYAERTKEKVNEG...
BnLEA86        ...........................................EDTKERAKEKAEDT...
BnLEA87        ...........................................EDTKERAKEKAEDT...
consensus>70   ..............................................kd............
```

```
                            80
BnLEA14        ........QSRTRHKKQQ..........................................
BnLEA16        ........KEGMAHKAHD......................VRDKVTKKAHNVKETMA...
BnLEA17        ........KEGMAHKAQD......................VKDKVTRKANNVKETMA...
BnLEA23        .......ASDFVAEKANV...................MEEEAVSITEKAR..........
BnLEA24        .......ASEFVTDKGKE...................TKEETVLMTEKA...........
BnLEA25        AYDKAGQAKDTAYDKAGQAKDMVYDTAG....SAYDKAGQAKDMAYDKAGSAYHKADQAK
BnLEA26        AYEKAGHAKDYAYEKAGDVKEVAYDKASNAKDMAYEKVDNVIDMTYDKVGSAYS...SAK
BnLEA34        ........KDAGVSKIGE......................LKDSAVDTAKRAMGFLSGKT
BnLEA35        ........KDAGVSKLGE......................FKDSAVDTAKRAMGFLSGKT
BnLEA36        ........KDKTAEKAGE......................YKDYTVEKAAEGK.......
BnLEA37        ........KDAGVSKIGE......................LKDSAVDTAKRAMGFLSGKT
BnLEA43        .......ALDYVKAKGN...........................................
BnLEA44        .......ASDYAREKARE...................AGHVGAQKEQEAK..........
BnLEA45        .......ASDYVTEKARE...................TGHVAAQKRQEAK..........
BnLEA54        ........KTSQTAQT............................................
BnLEA55        ........KTSQAAKT............................................
BnLEA56        ........KTSQAAQT............................................
BnLEA57        ........KTSQAAQT............................................
BnLEA58        ..DKS..WTDWAKEKIG....................IKP..KENYPNMG..........
BnLEA59        ..DKS..WTDWAKEKIG....................IKH..NENYPNMG..........
BnLEA60        ..DES..WTDWAKEKVG....................IKHGDDESYPNVG..........
BnLEA61        ..DES..WTDWAKEKVG....................IKHGDDESYPNVG..........
BnLEA85        ........AYKAADKAED......................TKER................
BnLEA86        ........MDSAKAKARD......................AKEKVKEYGE..........
BnLEA87        ........MDSAKAKARD......................AKEKVKEYGE..........
consensus>70   .........d....k.............................................
```

```
                                90
                                 .
BnLEA14        .........SIPKKLQKLVRIRP.....................................
BnLEA16        HKAHESKERVKDEVRDKARELKEKAAHKSHNAWERVK............LAARGL.....
BnLEA17        HKPHESKERVKDEVREKAHELKEKAAHKSHNAWERVK............FAARGL.....
BnLEA23        DFVVEKTGEAKDFIVEKAGDAKELATDMSKRTAIYVG.......................
BnLEA24        ..........KDFIVEKAGEAKDSATDMRKKTAKYVG.......................
BnLEA25        DMVSDKTGSAKDMVYGKAGQAKDMVNEKAAQAKEKA........GQAKDMVYDKAAQAK.
BnLEA26        DMAYDNAGNSKDMAYDKAGNAKDMAYEKAENVKDMTYDKVGSAYGNAKDMAYDKAGNVKD
BnLEA34        EETKQKTVETKETAKEKMNEAGEEARRKMEEMRLEGK............ELEEEA.....
BnLEA35        EEPKQKAVETKDSAKEKMVEAGEEARRKMEEMRLEGK............ELKEEARDKAR
BnLEA36        DAGVSKLGELKDSAKEKMEEAGEEARRKMEEMRLEGK............ELKDEARDKAR
BnLEA37        EETKQKTVETKETAKEKMNEAGEEARRKMEEMRLEGK............ELEEEA.....
BnLEA43        ..........................EAGNKVAEFVE.......................
BnLEA44        EQAAR....AKDYTMEKAGEAKETAAEKARRASQYAT.......................
BnLEA45        EQADR....GKD...........TAAEKARRASE..........................
BnLEA54        ..AQEKARETKDKTGSYLSETGEAVKQKAQDAAQYTK............ETAQNA.....
BnLEA55        ..AQEKAHETKDKTGSYMSETGEAIKQKAQNAAQYTK............ETAQEA.....
BnLEA56        ..TQDKARETKDKTGSYMSEAGEAIKNKAQDAAQYTK............ETAKGA.....
BnLEA57        ..AQEKARETKDKTGSYMSETGEAIKQKAQNAAQYTK............ETAQEA.....
BnLEA58        DTVSEKAKEAKDAATRKAADAKERLEETVEAAKEKAS...............DLTSAAK.
BnLEA59        DTVSEKAKEAKDAATRKAGDAKERLEETVEAAKEKAS...............DLTSAAK.
BnLEA60        DTVSEKAKEAKDAAKRKAGDAKVRLEETVEAAKEKAS...............DLTSAAK.
BnLEA61        DTVSEKAKEAKDAAKRKAGDAKVRLEETVEAAKEKAS...............DLTSAAK.
BnLEA85        ..AKEKAEGVKETVKGKAEELGEKTKETVKGAWETTK............NAAR.......
BnLEA86        .ETKEKAEGFKETVKGKAEELGEKTKETVKGAWENTK............DSAR.......
BnLEA87        .ETKEKAEGFKETVKVKAEELGEKTKESVKGAWENTK............DTAR.......
consensus>70   ....e.....kd....k..e..e...e......e..........................
```

```
                        100
                          .
BnLEA14        .....VGLWATW................................................
BnLEA16        ..GSATAKALSPTKVASVVGLTAIAAAFGTSVWVTFVSSYVLASVLGRQQFGVVQSKLYP
BnLEA17        ..GSATAKALSPTKVASVVGLTAIAAAFGTSVWVTFVSSYVLASVLGRQQFGVVQSKLYP
BnLEA23        ...EKAAEAKEAILPPKTEE........................................
BnLEA24        ...DKAAEAKEAIFPPKTEE........................................
BnLEA25        ...EKAGQAKDMAYNNAGQAKDKAGQSKDMAYDKAGQAKDMAFDKAG.......KAKDTV
BnLEA26        MAYEKAGNVKDMTYEKVGSAY...GSAKDMAYEKAGDAKDMVYDKVGAAYGSAEKAKDYG
BnLEA34        ..SKKTQERTESAADKARETKDSVSQR.................................
BnLEA35        EGSQKTKETADSAAERAHETKDSDAVR.................................
BnLEA36        EGSQKTKESAELAAERAHETKDSAVV..................................
BnLEA37        ..SKKTQERTESAADKARETKDSVSQR.................................
BnLEA43        ...GKAGEAKDATKA.............................................
BnLEA44        ...EKAKETANMTAEQAARAKDMALQKAAEAKDTAAEKAKYATEKGRETGITAAEQAARA
BnLEA45        ............................................................
BnLEA54        ..AQYTKETAEAGRDKTG..........................................
BnLEA55        ..AQYTKETAEAGRDKTG..........................................
BnLEA56        ..AQYTKETAEADRDKTG..........................................
BnLEA57        ..AQYTKETAEAGRDKTG..........................................
BnLEA58        ...EKAEKLKEEAERES...........................................
BnLEA59        ...EKAEKLKEEAESES...........................................
BnLEA60        ...EKAERLKEEAERER...........................................
BnLEA61        ...EKAERLKEEAERES...........................................
BnLEA85        ...TATEAVVGPEEDA............................................
BnLEA86        ...TVTEAVVGPEEDA............................................
BnLEA87        ...TVTEAVVGPEEDA............................................
consensus>70   ..........e.................................................
```

```
BnLEA14        ............................................................
BnLEA16        VYFKATSVGILVGLLGHVLSRRRKLLTDATEMWQGVNLLSAFFMIEANKSFVEPRATKAM
BnLEA17        VYFKATSVGILVGLLGHVLSRRRKLLTDATEMWQGVNLLSAFFMIEANKSFVEPRATKAM
BnLEA23        ............................................................
BnLEA24        ............................................................
BnLEA25        YDKADDVIRMATDKSDEAKE.IGYGTYKRAKEGSKNAKDVSFEKARDVRETGGQAMDYGK
BnLEA26        YEKTGDVIRMATDKSSEA........YEGAKERSNSAKDM.......VADKGEGAVKYGR
BnLEA34        ...EEGRGTIMGALGNMTGAIKSKLTGTTPSGDDV..................GSGKTTV
BnLEA35        ...GNEAKGTIFGAIGNVTEAIKSKLTMPSDIVEET..............RDRGSTGRTV
BnLEA36        .........TIFGTLENVTEAIKNKLTMPSDIVEETRV...........ARERGSTGRTV
BnLEA37        ...EEGRGTIMGALGNMTGAIKSKLTGTTPSGDDD..................LSGKTTV
BnLEA43        ............................................................
BnLEA44        KDLALQKAVEAKDIAAEKAARAKDYTLQKAVEAKDIAAEKAQRASQYVTEKGKETGNLTA
BnLEA45        ............................................................
BnLEA54        ...........GFLSQTGEHVKQMAMGAADAVKHT.....................FGMA
BnLEA55        ...........GFLSQTGEQVKQMAMGAADAVKHT.....................FGMA
BnLEA56        ...........GFLSQTGEHVKQIAMGAADAVKHT.....................FGMA
BnLEA57        ...........GFLSQTGEQVKQMAMGAADAVKHT.....................VGMA
BnLEA58        ........KNAKEKSKKH........YENAKS..................KAEETLESAK
BnLEA59        ........KNAKEKTKEH........YETARS..................KAEETLESAK
BnLEA60        ........KSAKDKSKES........YETAKS..................KADETLESAK
BnLEA61        ........KSAKDKSKES........YETAKS..................KADETLESAK
BnLEA85        ............................................................
BnLEA86        ............................................................
BnLEA87        ............................................................
consensus>70   ............................................................
```

```
BnLEA14        ............................................................
BnLEA16        FERMKAEKEEGRGGGGGERTSEQEVRRKLEKLSERLSKLNTYSSWLNIMMLMSLTWHFVY
BnLEA17        FERMKAEKEEGRGGG..ERTSEQEVRRKLERLSERLSKLNTYSSWLNIMMLMSLTWHFVY
BnLEA23        ............................................................
BnLEA24        ............................................................
BnLEA25        DKATDAYGLGNEAAG.KLEEAMYKVGERYGAAKDSTSEKAKEAYESAKEKASEATGEYGA
BnLEA26        DKATEAMDESVEYIKEKSHKAKDGAAKGFGETMDKVKETSKHAYETAKEKASHVAEE...
BnLEA34        TVDVVEDTRPGQVAT.....KLKAADQMTGQTFNDVGEMDEEDRKVNVTVGDKGKL....
BnLEA35        VEVTVEDTKPGKVAA.....TLKASDRMTSPTFNEIEVEDTKPGKVAATLKASDQMTGQT
BnLEA36        VEVTVEDTKPSKVAT.....TLKASDRMTDPTFNEIEVEDTKPGKVAATLKASDQMTGQT
BnLEA37        TVDVVEDTRPGQVAT.....KLKAADQMTGQTFNDVGEMDEEDRKVNVTVGDKGKL....
BnLEA43        ............................................................
BnLEA44        QKGQEAKEQTVSVTAKAKDYTVQKAGEAVEMSKEAAEYAKETVVEGGKGAAHYTGVAAEK
BnLEA45        ............................................................
BnLEA54        TEEEDREHYPG.........TTTGTTRSTDQTRHTYERK.....................
BnLEA55        TEEEDKEHYPGTTT......TTTGTTRTTDPTHHTYQRK.....................
BnLEA56        TEEDDRENFPG.........TTTGTTRTTDTTHQTYQGK.....................
BnLEA57        TEEEDREHYPGTTT......TTTGTTRTTDPTHHTYQRK.....................
BnLEA58        DKASQSYDS...............AAKESEEARDTLSHKSKRVKDTSFNEDD.EL.....
BnLEA59        DKASQSYDS...............AAKESEQARDNLSHKSKRVKDTSFNEED.EL.....
BnLEA60        DKASQSYDS...............AAKKTEQAKDSVSQKSKKVK.DTLNDDDAEL.....
BnLEA61        DKSSQSYDS...............AAKKTEQAKDSVSHKSKKVKEDTLNDDDAEL.....
BnLEA85        .DKARADIDKG..........VEDLTKKAEKKSEKDRKEDEFITFN..............
BnLEA86        .DEARADIDKG..........VEDLTKK................................
BnLEA87        .DEARADIDKG..........VEDLTKK................................
consensus>70   ............................................................
```

```
BnLEA14        ............................................................
BnLEA16        LGQRLGAAC...................................................
BnLEA17        LGQRLGAAC...................................................
BnLEA23        ............................................................
BnLEA24        ............................................................
BnLEA25        YLRDHSVEL...................................................
BnLEA26        .IERYVVEL...................................................
BnLEA34        ............................................................
BnLEA35        FNDVGRMDY...................................................
BnLEA36        FNDVGRMDY...................................................
BnLEA37        ............................................................
BnLEA43        ............................................................
BnLEA44        AGTVGWTAAHFTTEKVVQGTKAVAGTVEGAVGYAGHKAAEVGSKAVDLTKEKAAVAADTV
BnLEA45        ............................................................
BnLEA54        ............................................................
BnLEA55        ............................................................
BnLEA56        ............................................................
BnLEA57        ............................................................
BnLEA58        ............................................................
BnLEA59        ............................................................
BnLEA60        ............................................................
BnLEA61        ............................................................
BnLEA85        ............................................................
BnLEA86        ............................................................
BnLEA87        ............................................................
consensus>70   ............................................................
```

```
BnLEA14        ............................................................
BnLEA16        ............................................................
BnLEA17        ............................................................
BnLEA23        ............................................................
BnLEA24        ............................................................
BnLEA25        ............................................................
BnLEA26        ............................................................
BnLEA34        ............................................................
BnLEA35        ............................................................
BnLEA36        ............................................................
BnLEA37        ............................................................
BnLEA43        ............................................................
BnLEA44        VGYTARKKEEAQHRDQEMHQGGEEEKGRGYVTEPRGGFQEEYKGERGSTEEDVFGYGPKG
BnLEA45        ............................................................
BnLEA54        ............................................................
BnLEA55        ............................................................
BnLEA56        ............................................................
BnLEA57        ............................................................
BnLEA58        ............................................................
BnLEA59        ............................................................
BnLEA60        ............................................................
BnLEA61        ............................................................
BnLEA85        ............................................................
BnLEA86        ............................................................
BnLEA87        ............................................................
consensus>70   ............................................................
```

```
BnLEA14        ............................................................
BnLEA16        ............................................................
BnLEA17        ............................................................
BnLEA23        ............................................................
BnLEA24        ............................................................
BnLEA25        ............................................................
BnLEA26        ............................................................
BnLEA34        ............................................................
BnLEA35        ............................................................
BnLEA36        ............................................................
BnLEA37        ............................................................
BnLEA43        ............................................................
BnLEA44        FSGAERRDVREEYGRGRESEEDVFGYGAQGGVSRDVGEEEFYGGGGRRNERYAQEQGAGA
BnLEA45        ............................................................
BnLEA54        ............................................................
BnLEA55        ............................................................
BnLEA56        ............................................................
BnLEA57        ............................................................
BnLEA58        ............................................................
BnLEA59        ............................................................
BnLEA60        ............................................................
BnLEA61        ............................................................
BnLEA85        ............................................................
BnLEA86        ............................................................
BnLEA87        ............................................................
consensus>70   ............................................................
```

```
BnLEA14        ..............................................
BnLEA16        ..............................................
BnLEA17        ..............................................
BnLEA23        ..............................................
BnLEA24        ..............................................
BnLEA25        ..............................................
BnLEA26        ..............................................
BnLEA34        ..............................................
BnLEA35        ..............................................
BnLEA36        ..............................................
BnLEA37        ..............................................
BnLEA43        ..............................................
BnLEA44        GGVLGAIGETIAEIAKTTTNIVIGDPPERTHEHGTAGYMGQEHGRR
BnLEA45        ..............................................
BnLEA54        ..............................................
BnLEA55        ..............................................
BnLEA56        ..............................................
BnLEA57        ..............................................
BnLEA58        ..............................................
BnLEA59        ..............................................
BnLEA60        ..............................................
BnLEA61        ..............................................
BnLEA85        ..............................................
BnLEA86        ..............................................
BnLEA87        ..............................................
consensus>70   ..............................................
```

BnLEA proteins of LEA_4 family

```
             1        10        20        30        40
BnLEA3       ..MARSLST.AKTLSVIVAEGFS.NAIYRRGFAVAADTALHGSVASGGTTAST.......
BnLEA4       ..MARSLST.AKTLSVIVAGEFS.NAIFRRGFAVAADTALHGSVGSGGTTASA.......
BnLEA74      ..MMGAMSQSLFNLKSFS.CSLN.NIIVMRGYILIK.KATQRAYTIGNSREKPSWTADCE
BnLEA75      ..MMGAMSQSLFNLKSLS.CSLN.NIIVMRGYILIK.KATQRAYAIGNSREKPSWTADCE
BnLEA76      ..MARSLSN.VKFVSAFVSQELS.NAIFRRGYAA...TAAQSSSGKGRAVVS........
BnLEA77      ..MSRSLSN.VKIVSSFVSHELS.NAIFRRGYAA...TAAQASVGKGGAVVS........
BnLEA78      ..MARSLSS.VKFVSAFVSQELS.NAIFRRGFAA...TTAQPSGGKGGAVVS........
BnLEA79      ..MARSLSN.VKFVSAFVSQELS.NAIFR............KGRAVVS........
BnLEA80      ..MARSLSN.VKIVSAFVSRELS.NAIFRRGYAA...TAAQASVGKGGAVVS........
BnLEA81      ..MARSLSS.VKFVSAFVSQELS.NAIFRRGFAA...TTAQPSGGKGGAVVS........
BnLEA82      MAAARSLSGAVKSLCSAASHNISGSIVLRRSYVAT.....VPGFGKGGSTRV........
BnLEA83      MAAARSLSAGVKSLYSAVSHNFSGSIVLRRSYVAT.....VPGFGKGGSTRV........
BnLEA84      ..........................MFHQSYVE..........................
consensus>70 ..m.rsls...k..........s.n.i.rr.y.............g............

                       50        60          70        80        90
BnLEA3       ..........AAMKKNVGEESSEKAP........WIPDPKTGYYRPATVSEEIDPAELRA
BnLEA4       ..........SAMKKNVGEESSEKAP........WIPDPKTGYYRPATVSQEIDPAELRA
BnLEA74      TGYYRPETITKELDSYVVTTSNAEVKMGRGEKLWWMPDPQTGFYRPDTFARELDAVELRS
BnLEA75      TGYYIPETITKELDSYVVTTSNAEVKMRRGEELWWMPDPQTGFYRPDTFARELDAVELRS
BnLEA76      ..........AVMKK.GVEESNKKIS........WIPDPKTGYYRPETGSNEIDPAELRA
BnLEA77      ..........AVMKKKGVEESTQKIA........WIPDPKTGYYRPETGSKEIDPAELRA
BnLEA78      ..........AVMKK.GVEESNQKIAA.......WIPDPKTGYYRPETGSNEIDPAELRA
BnLEA79      ..........AVMKK.GVEESNKKIS........WIPDPKTGYYRPETGSNEIDPAELRA
BnLEA80      ..........GVMKKKGVEESTQKIA........WIPDPKTGYYRPETGSKEIDPAELRA
BnLEA81      ..........AVVKK.GVEESNQKIAA.......WIPDPKTGYYRPETGSNEIDPAELRA
BnLEA82      ...........TVGKMEQRANQEAESA.......WAPDPVTGYYRPSNRADEIDPAELRE
BnLEA83      ...........TVGKLEQRANQEAESA.......WAPDPVTGYYRPFNRADEIDPAELRE
BnLEA84      .............KLEQRANQEAESA.......WAPDPVTGYYRPSNCADEIDPAELRK
consensus>70 .............k.....s............W.PDP.TG%YRP.t...EiDpaELR.

BnLEA3       VLLNNKQ...
BnLEA4       VLLNNKQ...
BnLEA74      MHSNHRE...
BnLEA75      MHSNHRE...
BnLEA76      ALLNKKQ...
BnLEA77      ALLNNKQ...
BnLEA78      ALLNKKQ...
BnLEA79      ALLDKKQ...
BnLEA80      ALLNNKQ...
BnLEA81      ALLNKKQ...
BnLEA82      MLLKNKAKPF
BnLEA83      MLLKNKAKPL
BnLEA84      MLLENKAKPF
consensus>70 .lln.kq...
```

BnLEA proteins of LEA_3 family

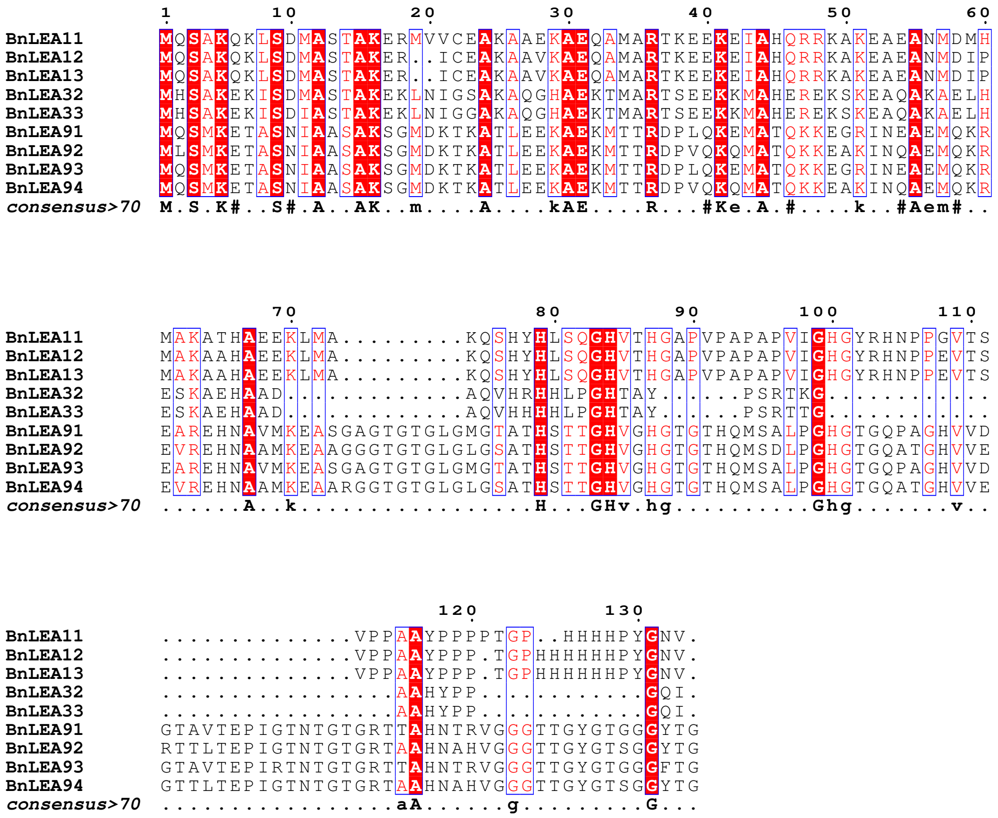

BnLEA proteins of LEA_1 family

```
                 1        10        20        30        40        50
BnLEA38          MAS.QQEKKQLDERAKKGETVVQGGTGGKSFEAQQHLAEGRSRGGNTRKEQLGTEGYQQM
BnLEA39          MAS.HQEKKQLDKRAKKGETVVPGGSGGRSFEAQQHLAEGRSRGGNTRKKQLGTKGYQKM
BnLEA40          MAS.QQEKKQLDERAKKGETVVPGGTGGKSFEAQQHLAEGRSRGGNTRKEQLGSEGYQQI
BnLEA41          MAS.QQEKKQLDERAKKGETVVQGGTGGKSFEAQQHLAEGRSRGGTTRKEQLGTEGYQQM
BnLEA42          MAS.QQEKKQLDERAKKGETVVPGGTGGKSFEAQQHLAEGRSRGGNTRKEQLGSEGYQQM
BnLEA72          MASKQQSREKLDEKARQGETVVPGGTGGKSVEAQERLAEGRSKGGQTRKEQLGHEGYQEM
BnLEA73          MASKQQSREELDEKAKQGETVVPGGTGGKSVEAQERLAEGRSKGGQTRREQLGHEGYQEI
consensus>70     MAS.qQekkqLDerAkkGETVVpGGtGGkSfEAQ#hLAEGRSrGGnTRkeQLG.eGYQqm

                60        70        80
BnLEA38          GRKGGTRTGKADDED........EGEEMDEPTSRTRT.......................
BnLEA39          GRKGGMNTKDKPGED........QGEEMDEPNPGPIPNLKLSDWMCKSFVLIFPYT....
BnLEA40          GRKGGHSTRDKTDEE........DAE..DESRTRT.........................
BnLEA41          GRKGGARTGKADDED........EGEEMDEPTSRTRT.......................
BnLEA42          GRQGGHSTRDKTDEE........DAE..DESSIRT.........................
BnLEA72          GHKGGETRKEQLGHEGYQEIGHKGGETRKEQLLGHEGYQEMGHKGGETRKEQLGHEGYKE
BnLEA73          GHKGGETRKEQLGHEGYQEMGHKGGETRKEQ.LGHGGYQEMGHKGGETRKEQLGHEGYKE
consensus>70     GrkGG..t.d...e#........egE..dE..............................

BnLEA38          .................................
BnLEA39          .CVFGFLS.........................
BnLEA40          .................................
BnLEA41          .................................
BnLEA42          .................................
BnLEA72          MGRKGGLSTMDKSGGERAEEEGIEIDESKFTNK
BnLEA73          MGRKGGLSTMDKSGGERAEEEGIEIDESKFTNK
consensus>70     .................................
```

BnLEA proteins of LEA_5 family

```
BnLEA11         ............................................................
BnLEA12         ............................................................
BnLEA13         ............................................................
BnLEA32         ............................................................
BnLEA33         ............................................................
BnLEA91         ............................................................
BnLEA92         ............................................................
BnLEA93         ............................................................
BnLEA94         ............................................................
BnLEA1          ............................................................
BnLEA2          ............................................................
BnLEA46         ............................................................
BnLEA47         MSTSENKVEIVDRAHKEEEKEEDGKGGFLDKVKDFIHDIGEKIEGAIGFGKPTADVSAIH
BnLEA48         ............................................................
BnLEA49         ............................................................
BnLEA50         ............................................................
BnLEA51         ............................................................
BnLEA52         ............................................................
BnLEA53         ............................................................
consensus>70    ............................................................

BnLEA11         ............................................................
BnLEA12         ............................................................
BnLEA13         ............................................................
BnLEA32         ............................................................
BnLEA33         ............................................................
BnLEA91         ............................................................
BnLEA92         ............................................................
BnLEA93         ............................................................
BnLEA94         ............................................................
BnLEA1          ............................................................
BnLEA2          ............................................................
BnLEA46         ............................................................
BnLEA47         IPKINLERADIVVDVLVKNPNPVPIPLIDIDYLIESDGRKLVSGLIPDAGTIKAHGEETV
BnLEA48         ............................................................
BnLEA49         ............................................................
BnLEA50         ............................................................
BnLEA51         ............................................................
BnLEA52         ............................................................
BnLEA53         ............................................................
consensus>70    ............................................................

                                      1       10       20       30
BnLEA11         ......................MQSAKQKLSDMASTAKERMVVCEAKAAEKAEQAMARTK
BnLEA12         ......................MQSAKQKLSDMASTAKER..ICEAKAAVKAEQAMARTK
BnLEA13         ......................MQSAKQKLSDMASTAKER..ICEAKAAVKAEQAMARTK
BnLEA32         ......................MHSAKEKISDMASTAKEKLNIGSAKAQGHAEKTMARTS
BnLEA33         ......................MHSAKEKISDIASTAKEKLNIGGAKAQGHAEKTMARTS
BnLEA91         ......................MQSMKETASNIAASAKSGMDKTKATLEEKAEKMTTRDP
BnLEA92         ......................MLSMKETASNIAASAKSGMDKTKATLEEKAEKMTTRDP
BnLEA93         ......................MQSMKETASNIAASAKSGMDKTKATLEEKAEKMTTRDP
BnLEA94         ......................MQSMKETASNIAASAKSGMDKTKATLEEKAEKMTTRDP
BnLEA1          .....................MAS...............LLDKAKDFVADKLAGVPKPEG
BnLEA2          .....................MAS...............LLDKAKDFVADKLAGVPKPEG
BnLEA46         .....................MIIPYRIKVDLIVDVP..VLGRLT.LPLEKRGEIPIPKK
BnLEA47         KIPLTLIYDDIKSTYNDINPGMIIPYRIKVDLIVDVP..VLGRLT.LPLEKRGEIPIPKK
BnLEA48         .....................MIIPYRIKVDLIVDVP..VLGRLT.LPLEKRGEIPIPKK
BnLEA49         .....................MASTEQKEVEENGSMISGLLDKAKGFFAEKLANVPTPEA
BnLEA50         .....................MASSEQKLVEENGSVISSLLDKAKGFIAEKLANIPTPEA
BnLEA51         .....................MASTEQKEVEENGSMISGLLDKAKGFFAEKLANVPTPEA
BnLEA52         .....................MASSEQKLVEENGSVISNLLDKAKGFIAEKLANIPTPEA
BnLEA53         .....................MAETEQKEVEEKGSLISGLLDKAKGFFAEKLANIPTPEA
consensus>70    ...............................d........l........ek.........

                     40        50        60        70
BnLEA11         E.....EKEIAHQRRKAKEAEANMDMHMAKATHAEEKLMA...............KQSHY
BnLEA12         E.....EKEIAHQRRKAKEAEANMDIPMAKAAHAEEKLMA...............KQSHY
BnLEA13         E.....EKEIAHQRRKAKEAEANMDIPMAKAAHAEEKLMA...............KQSHY
BnLEA32         E.....EKKMAHEREKSKEAQAKAELHESKAEHAAD...................AQVHR
BnLEA33         E.....EKKMAHEREKSKEAQAKAELHESKAEHAAD...................AQVHH
BnLEA91         L.....QKEMATQKKEGRINEAEMQKREAREHNAVMKEASGAGT......GTGLGMGTAT
BnLEA92         V.....QKQMATQKKEAKINQAEMQKREVREHNAAMKEAAGGGT......GTGLGLGSAT
BnLEA93         L.....QKEMATQKKEGRINEAEMQKREAREHNAVMKEASGAGT......GTGLGMGTAT
BnLEA94         V.....QKQMATQKKEAKINQAEMQKREVREHNAAMKEAARGGT......GTGLGLGSAT
BnLEA1          S...VTDVDLKDVNRDSVEYLAKVSVTNPYGHAIPICEINFTIHSGGREIGKGKIPDPGS
BnLEA2          S...VTDVDLKDVNRDSVEYLAKVSVTNPYGHAIPICELNFTIHSAGREIGKGKIPDPGS
BnLEA46         PDVDIEKIKFQKFSLEETVAILHVRLENLNDFDLGVNDLDCEVWLSDVSIGKAEISDSIK
BnLEA47         PDVDIEKIKFQKFSLEETVAILHVRLENLNDFDLGVNDLDCEVWLSDVSIGKAEISDSVK
BnLEA48         PNVDVEKIKFQKFSLEETVAILHVRLENMNDFDLGLNDLDCEVWLCDVSIGKAEISDSIK
BnLEA49         A...VDNVDFKGVTRQGVDYHAKVSVKNPYSQTIPICQISYVLKSATR.....TIPDPGS
BnLEA50         T...VDDVDFKGVSRQGVDYHAKVSVKNPYSQSIPICQISYILKSATRTIASGTIPDPGS
BnLEA51         A...VDNVDFKGVTRQGVDYHAKVSVKNPYSQTIPICQISYVLKSATR.....TIPDPGS
BnLEA52         T...VDDVDFKGVSRQGVDYHAKVSVKNPYSQSIPICQISYILKSATRTIASGTIPDPGS
BnLEA53         T...VDDVDFKGVTRQGVDYHAKVSVKNPYPQHIPICQISYILKSDTR..ASGTIPDPGS
consensus>70    ......e.d......e.....a.....n.........e..................d...
```

```
                80        90        100                 110       120
BnLEA11         HLSQGHVTHGAPVPAPAPVIGHGYRHNP..............PGVTSVPPAAYPPPPTGP
BnLEA12         HLSQGHVTHGAPVPAPAPVIGHGYRHNP..............PEVTSVPPAAYPPP.TGP
BnLEA13         HLSQGHVTHGAPVPAPAPVIGHGYRHNP..............PEVTSVPPAAYPPP.TGP
BnLEA32         HHLPGHTAY......PSRTKG.............................AAHYPP....
BnLEA33         HHLPGHTAY......PSRTTG.............................AAHYPP....
BnLEA91         HSTTGHVGHGTGTHQMSALPGHGTGQPAGHVVDGTAVTEPIGTNTGTGRTTAHNTRVGGG
BnLEA92         HSTTGHVGHGTGTHQMSDLPGHGTGQATGHVVERTTLTEPIGTNTGTGRTAAHNAHVGGG
BnLEA93         HSTTGHVGHGTGTHQMSALPGHGTGQPAGHVVDGTAVTEPIRTNTGTGRTTAHNTRVGGG
BnLEA94         HSTTGHVGHGTGTHQMSALPGHGTGQATGHVVEGTTLTEPIGTNTGTGRTAAHNAHVGGG
BnLEA1          LKAKDMTVLDVPIVVPYSILFNLARDVGADWDIDYLLEIGLTIDLPVVGDFTIPVTSKG.
BnLEA2          LKAKDMTVLDVPIVVSYSILFNLARDVGADWDIDYLLEIGLSIDLPVLGDFTIPVTSKG.
BnLEA46         LDKNGSGLINVPITFRPKDFGSALWDMIRGKGTGYTIKGNVDVDTP.FGGMKLPIIKEGG
BnLEA47         LDKNGSGLINVPITFRPKDFGSALWDMIRGKGTGYTIKGNVDVDTP.FGGMKLPIIKEGG
BnLEA48         LDKNGSGLVNVPMTFKPKDFGSALWDMIRGKGTGYTIKGNVDVDTP.FGAMKLPIIKEGG
BnLEA49         LVGNKTTVLDVPVKVAYSIAVSLMKDIGSDWDIDYQLDIGLTFDIPVVGDITIPVSTQG.
BnLEA50         LVGKGTTVLDVPVKVAYGIAVSLMKDIGSDWDIDYQLDIGLTIDIPVVGDITIPVSTKG.
BnLEA51         LVGNKTTVLDVPVKVAYSIAVSLMKDIGSDWDIDYQLDIGLTFDIPVVGDITIPVSTQG.
BnLEA52         LVGKGTTVLDVPVKVAYGIAVSLMKDIGSDWDIDYQLDIGLTIDIPVVGDITIPVSTKG.
BnLEA53         LIANGSTVLDVPVKVPYSIAVSLMKDMCLDWDIDYQLDIGLTIDIPIVGDITIPVSTQG.
consensus>70    ....g....................d.................d..............g.

                     130
BnLEA11         ..HHHHPYGNV......................
BnLEA12         HHHHHHPYGNV......................
BnLEA13         HHHHHHPYGNV......................
BnLEA32         ........GQI......................
BnLEA33         ........GQI......................
BnLEA91         TTGYGTGGGYTG.....................
BnLEA92         TTGYGTSGGYTG.....................
BnLEA93         TTGYGTGGGFTG.....................
BnLEA94         TTGYGTSGGYTG.....................
BnLEA1          EIKLPTFKDYF......................
BnLEA2          EIKLPTFKDYF......................
BnLEA46         ETRLKKEDDDDDD.EVIISSSLLLVT.......
BnLEA47         ETRLKKEDDDDDD.EVIISSSLLLVA.......
BnLEA48         ETRLKKEDDDDDDDEVGICSSCLFTYKIEHCVV
BnLEA49         EIKLPSLRDFF......................
BnLEA50         EIKLPSLRDFF......................
BnLEA51         EIKLPSLRDFF......................
BnLEA52         EIKLPSLRDFF......................
BnLEA53         EMKLPSLRDFF......................
consensus>70    .................................
```

BnLEA proteins of LEA_2 family

```
             1        10                  20                  30
BnLEA5       MAQQQNSP....RDQRD..............SRPQGDVFSVSG.........DDDVARKQ
BnLEA6       MAQQQNSP....RDQRD..............SRQHGDVFSVSG.........DDDVARKQ
BnLEA62      MSQQEQPKRPQEPVKYGDVFEVSGELADRRIAPEDARMMQAKETSALGHTQKGGIAATMQ
BnLEA63      MSQQEQPRRPQEPVKYGDVFEVSGELADKTVAPEDANMMQAAETRVFGHTQKGGTAAVMQ
BnLEA64      MSQQEQPKRPQEPVKYGDVFEVSGELADRPIAPEDARMMQAKETSVLGHTQKGGIAATMQ
BnLEA65      MSQQEQPRRPQEPVKYGDVFEVSGELADKTIAPEDANMMQAAETRVFGHTQKGGTAAVMQ
BnLEA66      MSQQEQPRRPQEPVKYGDVFEVSGELADKTIAPEDANMMQAAETRVFGHTQKGGTAAVMQ
BnLEA95      MSEDQVEK....PTTND..............VKEEAKKIPATQGGVDAADDKDKGVVTEA
BnLEA96      MNVEQLEK....PITYD..............VKHEAEKIPATEKSSEAAEDKEKGVVADA
BnLEA97      MSQEKLEKK...PITYD..............VKEEAKKTPATEGGI.ATDDKEKGVVAES
BnLEA98      MSEDQVEK....PTTND..............VKEEAKKIPATQGGVDAADDKDKGVVTEA
BnLEA99      MCVEQLEK....PITYD..............VKQEAEKIPAT................EK
BnLEA100     MSQEQLEK....PIAND..............VKDEAKKTPATEGGI.AAYDKEKGVVAEP
BnLEA101     MTASKDGAD...FTNIS..............VEEHFRVSQSNHG........GQFVGPTE
BnLEA102     MTASKDGANS..FTNIS..............VEEHFSVSQSTSG........GQFVGPTE
BnLEA103     MTASKDGAS...FTNIS..............VEEHFRVSQSNHG........GQFVGPTE
consensus>70 M..qq............................ee.........................

                   40
BnLEA5       GAGSSNP.....................GPKIVT..........................
BnLEA6       GAGSSKP.....................GPAIVT..........................
BnLEA62      SAATANRRGGFVEPGVATYLDPDRGVSVDQTDVAGARVTKESIGVQDVGQYVEPRPVSTA
BnLEA63      SAATANKRGGFVQQGDATDVAAEHGVTVAQTDVPGARVTTEFVGGQVVGQYVEPMPVGTT
BnLEA64      SAATANRRAGFVEPGVATYLDPDRGVSVEQTDVAGARVTKETIGVQVVGQYVEPRPVATA
BnLEA65      SAATANKRGGFVQQGDATDVAAEHGVTVAQTDVPGARVTTEFVGGQVVGQHVEPMPVGTT
BnLEA66      SAATANKRGGFVQQGDATDVAAEHGVTVAQTDVPGARVTTEFVGGQVVGQYVEPMPVGTT
BnLEA95      SGGQAEG...................EVNQKNVV.................ANPP.....
BnLEA96      SGGQAEG...................EVNEKKIV.................ANPP.....
BnLEA97      TGVQVEG...................EVNQK.....................KP......
BnLEA98      SGGQAEG...................EVNQKNVV.................ANPP.....
BnLEA99      SSEAAEG...................EVRKEKVV.................ANPP.....
BnLEA100     SGGQVEG...................EVNQK.....................KP......
BnLEA101     EISTAAN.....................................................
BnLEA102     EISTAAD.....................................................
BnLEA103     EFSTAAN.....................................................
consensus>70 ....an......................................................

                             50        60        70        80        90
BnLEA5       .........MGSVDTVTIGEALEVTALSLGDKPVDRKDAAAIQAAETRATGDSKTRPGGL
BnLEA6       .........MGSVDTVTIGEALEVTALSLGDKPVDRKDAAAIQAAETRATGDSKTRPGGL
BnLEA62      AM......GVSVQSKITIGQALEATVHTAGKKPVDQSDAAAIQAAEVRASSNNVIAPGGV
BnLEA63      AATDAETLGLSLQSAITIGEALEAAVQTAGNKPVDQSDAAAIQAAEVRASGTSVIAPGGI
BnLEA64      AT......GVSVQSKITIGQALEAAVHTAGKKPVDQSDAAAIQAAEVRASSNNVIAPGGV
BnLEA65      AATDAETLGLNLQSAITIGEALEAAVQTAGNKPVDQSDAAAIQAAEVRASGTSVIAPGGI
BnLEA66      AATDAETLGMNLQSAITIGEALEAAVQTAGNKPVDQSDATAIQAAEVRASGTSDIAPGGI
BnLEA95      ..........ASEGTITIGEALEAAVLTAGNKPVEWSDAAAIQAAEVRATGRTNIMPGGV
BnLEA96      ..........ASEGTITIGEALEAAVLTAGNKPVEWSDAAAIQAAEVRATGRTNIMPGGV
BnLEA97      ............EGTITIGEALEAAVLTAGNKPVEWSDAAAIQAAEVRATGKTNIMPGGV
BnLEA98      ..........ASEGTITIGEALEAAVLTAGNKPVEWSDAAAIQAAEVRATGRTNIMPGGV
BnLEA99      ..........ASEVTITIGEALDATVLTAGNKPVEWSDAAAIQAAEVRATGRTNIMPGGV
BnLEA100     ............EGTITIGEALEAAVLTAGNKPVEWSDAAAIQAAEVRATGKTNIMPGGV
BnLEA101     .........ALIGRSARLTEALKAASINVGHKPVETRDLAAIKEVEARATG...ESGGSI
BnLEA102     .........ALIGRSATLTEALKAAAINVGHKPVETTDLAAIKELEARATGGKIERGDSV
BnLEA103     .........ALIGRSARLTEALKAASINVGYKPVETRDLAAIKEVEARATG...ESGGSI
consensus>70 ...............itig#ALea.....G.KPV#..DaaAIqaaE.RA.g.....pggv

             100       110        120       130       140       150
BnLEA5       AEAAQEAAATNERTALEEA.KVTIADILTVITFFITFDKVVTSEDAEAVVGAELRNSPEM
BnLEA6       AEAAQEAAATNERTALEEA.KVTIADILTVITFFITFDKVVTSEDAEAVVGAELKNSPEM
BnLEA62      AASAQSAADYNAPIEFDEN.KIKLADVLTV...KPQSQTHFQRLTLCFLINVFE...PDS
BnLEA63      AASAQSAAIHNATVDRDEE.KIKLVDVLAGATGKLQADKAVTRQDAEGVVSAELRNNPNL
BnLEA64      AASAQSAADYNAPIEFDEN.KIKLADVLAAG..KLQEDKAVTKQDAEGVVSAELRNNPNL
BnLEA65      AASAQSAANHNATVDRDED.KIKLVDVLAGATGKLQADKAVTRQDAEGVVSAELRNNPNL
BnLEA66      AASARSAANHNATVDRDED.KIKLVDVLAGATGKLQADKAVTRQDAEGVVSAELRNNPYL
BnLEA95      AASAQSAATLNARANSDEE.KTTLADVLTGARGKLPSDKPATRKDAEGVTGAEMRNDPHL
BnLEA96      AASAQSAATLNARANSEDD.KTTLAVVLTGARSKLPSDKPATRKDAEGVTGAEMRNDPHL
BnLEA97      AASAQSAATLNARANSEDD.KTTLADVLTGASSKLPSDKPATRKDAEGVTGAEMRNDPHL
BnLEA98      AASAQSAATLNARVNSDDE.KTTLADVLTGARGKLPSDKPATRKDAEGVTGAEMRNDPHL
BnLEA99      AASAQSAATLNARANSEDD.KTTLAVVLTVRQTRHEEG....RRGSD.RCGDEERSSPHY
BnLEA100     AASAQSAATLNARANSEDD.KTTLADVLTGASSKLPSDKPATRKDAEGVTGAEMRNDPHL
BnLEA101     TAVANEAVARNKKIGKEDENKIHLRDIIAEIDVKITRDRSVTSEDAEAVVQAELTHPPYN
BnLEA102     TSMANEAVARNKKIGKEDDKKIRLRDIVAEIDVRVTRDRSVTSEDAEAVVQAELNHSPYN
BnLEA103     TAVANEAVTRNKKIGKEDENKIHLRDIIAEIDVKITRDRSVTSEDAEAVVQAELTHPPYN
consensus>70 aa.Aq.Aa..N.....##d.K..l.d!l.....k...d...t..dae.v..ael...P..
```

```
                160       170
BnLEA5          KTTPGGVADSMSAGARLNQPL..
BnLEA6          KTTPGGVADSMSAGARLNQPL..
BnLEA62         RVLRG.......SYKRINP....
BnLEA63         TTHPGGVAASVTAAARLNEKADI
BnLEA64         STYPGGVADSVTAAARLNAKGDI
BnLEA65         TTHPGGVAASVTAAARLNEKADI
BnLEA66         TIHPGGVAASVTAAARLNEKADI
BnLEA95         TTYPTGVAASVAAAARINQSK..
BnLEA96         TTYPTGVAASVAAAARINQAK..
BnLEA97         TTYPTGVAASVAAAARINQAK..
BnLEA98         TTYPTGVAASVAAAARINQSK..
BnLEA99         ..LPCWVPPRWREPSGHET....
BnLEA100        TTYPTGVAASVAAAARINQAK..
BnLEA101        HVIPGGVAESVTAAYRLNRSPSL
BnLEA102        HVIPGGVAESVAAAYKLNRSPSM
BnLEA103        HIIPGGVAESVTAAYRLNRSPSL
consensus>70    ...p.gva.sv.aa.r.#.....
```

BnLEA proteins of SMP family

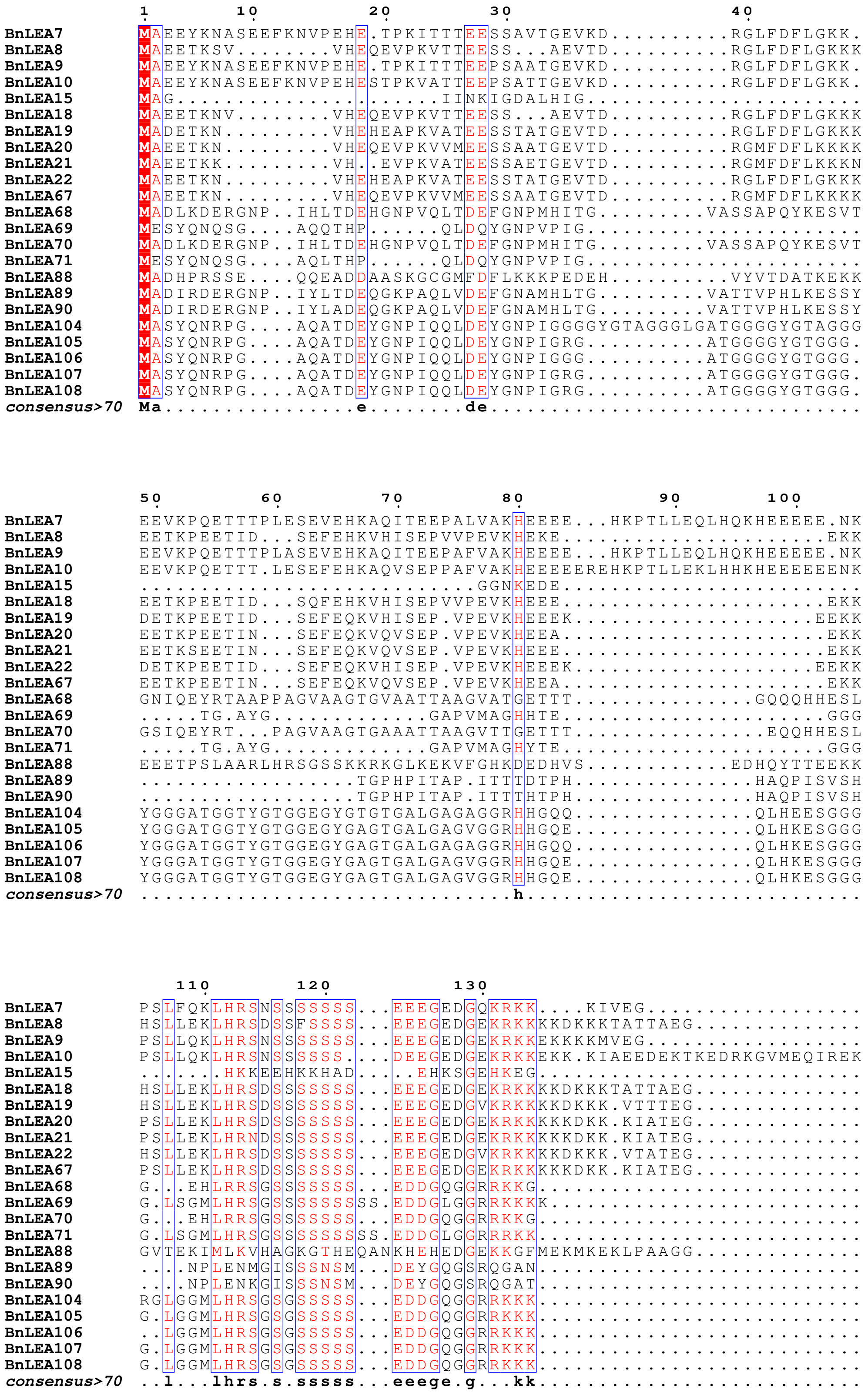
1 10 20 30 40
BnLEA7 MAEEYKNASEEFKNVPEHE.TPKITTTEESSAVTGEVKD..........RGLFDFLGKK.
BnLEA8 MAEETKSV........VHEQEVPKVTTEESS...AEVTD..........RGLFDFLGKKK
BnLEA9 MAEEYKNASEEFKNVPEHE.TPKITTTEEPSAATGEVKD..........RGLFDFLGKK.
BnLEA10 MAEEYKNASEEFKNVPEHESTPKVATTEEPSATTGEVKD..........RGLFDFLGKK.
BnLEA15 MAG......................IINKIGDALHIG.......................
BnLEA18 MAEETKNV........VHEQEVPKVTTEESS...AEVTD..........RGLFDFLGKKK
BnLEA19 MADETKN.........VHEHEAPKVATEESSTATGEVTD..........RGLFDFLGKKK
BnLEA20 MAEETKN.........VHEQEVPKVVMEESSAATGEVTD..........RGMFDFLKKKK
BnLEA21 MAEETKK.........VH..EVPKVATEESSAETGEVTD..........RGMFDFLKKKN
BnLEA22 MAEETKN.........VHEHEAPKVATEESSTATGEVTD..........RGLFDFLGKKK
BnLEA67 MAEETKN.........VHEQEVPKVVMEESSAATGEVTD..........RGMFDFLKKKK
BnLEA68 MADLKDERGNP..IHLTDEHGNPVQLTDEFGNPMHITG.........VASSAPQYKESVT
BnLEA69 MESYQNQSG....AQQTHP......QLDQYGNPVPIG.......................
BnLEA70 MADLKDERGNP..IHLTDEHGNPVQLTDEFGNPMHITG.........VASSAPQYKESVT
BnLEA71 MESYQNQSG....AQLTHP......QLDQYGNPVPIG.......................
BnLEA88 MADHPRSSE....QQEADDAASKGCGMFDFLKKKPEDEH..........VYVTDATKEKK
BnLEA89 MADIRDERGNP..IYLTDEQGKPAQLVDEFGNAMHLTG.........VATTVPHLKESSY
BnLEA90 MADIRDERGNP..IYLADEQGKPAQLVDEFGNAMHLTG.........VATTVPHLKESSY
BnLEA104 MASYQNRPG....AQATDEYGNPIQQLDEYGNPIGGGGYGTAGGGLGATGGGGYGTAGGG
BnLEA105 MASYQNRPG....AQATDEYGNPIQQLDEYGNPIGRG..........ATGGGGYGTGGG.
BnLEA106 MASYQNRPG....AQATDEYGNPIQQLDEYGNPIGGG..........ATGGGGYGTGGG.
BnLEA107 MASYQNRPG....AQATDEYGNPIQQLDEYGNPIGRG..........ATGGGGYGTGGG.
BnLEA108 MASYQNRPG....AQATDEYGNPIQQLDEYGNPIGRG..........ATGGGGYGTGGG.
consensus>70 Ma................e........de...............................
50 60 70 80 90 100
BnLEA7 EEVKPQETTTPLESEVEHKAQITEEPALVAKHEEEE...HKPTLLEQLHQKHEEEEE.NK
BnLEA8 EETKPEETID...SEFEHKVHISEPVVPEVKHEKE......................EKK
BnLEA9 EEVKPQETTTPLASEVEHKAQITEEPAFVAKHEEEE...HKPTLLEQLHQKHEEEEE.NK
BnLEA10 EEVKPQETTT.LESEFEHKAQVSEPPAFVAKHEEEEEREHKPTLLEKLHHKHEEEEEENK
BnLEA15 ....GGNKEDE.........................
BnLEA18 EETKPEETID...SQFEHKVHISEPVVPEVKHEEE......................EKK
BnLEA19 DETKPEETID...SEFEQKVHISEP.VPEVKHEEEK....................EEKK
BnLEA20 EETKPEETIN...SEFEQKVQVSEP.VPEVKHEEA......................EKK
BnLEA21 EETKSEETIN...SEFEQKVQVSEP.VPEVKHEEE......................EKK
BnLEA22 DETKPEETID...SEFEQKVHISEP.VPEVKHEEEK....................EEKK
BnLEA67 EETKPEETIN...SEFEQKVQVSEP.VPEVKHEEA......................EKK
BnLEA68 GNIQEYRTAAPPAGVAAGTGVAATTAAGVATGETTT...............GQQQHHESL
BnLEA69 .....TG.AYG............GAPVMAGHHTE......................GGG
BnLEA70 GSIQEYRT...PAGVAAGTGAAATTAAGVTTGETTT...............EQQHHESL
BnLEA71 .....TG.AYG............GAPVMAGHYTE......................GGG
BnLEA88 EEETPSLAARLHRSGSSKKRKGLKEKVFGHKDEDHVS...........EDHQYTTEEKK
BnLEA89 ..................TGPHPITAP.ITTTDTPH...............HAQPISVSH
BnLEA90 ..................TGPHPITAP.ITTTHTPH...............HAQPISVSH
BnLEA104 YGGGATGGTYGTGGEGYGTGTGALGAGAGGRHHGQQ...............QLHEESGGG
BnLEA105 YGGGATGGTYGTGGEGYGAGTGALGAGVGGRHHGQE...............QLHKESGGG
BnLEA106 YGGGATGGTYGTGGEGYGAGTGALGAGAGGRHHGQQ...............QLHKESGGG
BnLEA107 YGGGATGGTYGTGGEGYGAGTGALGAGVGGRHHGQE...............QLHKESGGG
BnLEA108 YGGGATGGTYGTGGEGYGAGTGALGAGVGGRHHGQE...............QLHKESGGG
consensus>70 ....h............................
110 120 130
BnLEA7 PSLFQKLHRSNSSSSSS...EEEGEDGQKRKK....KIVEG...................
BnLEA8 HSLLEKLHRSDSSFSSSS...EEEGEDGEKRKKKKDKKKTATTAEG..............
BnLEA9 PSLLQKLHRSNSSSSSS...EEEGEDGEKRKKEKKKKMVEG...................
BnLEA10 PSLLQKLHRSNSSSSS....DEEGEDGEKRKKEKK.KIAEEDEKTKEDRKGVMEQIREK
BnLEA15 ......HKKEEHKKHAD.....EHKSGEHKEG............................
BnLEA18 HSLLEKLHRSDSSSSSSS...EEEGEDGEKRKKKKDKKKTATTAEG..............
BnLEA19 HSLLEKLHRSDSSSSSSS...EEEGEDGVKRKKKKDKKK.VTTTEG..............
BnLEA20 PSLLEKLHRSDSSSSSSS...EEEGEDGEKRKKKKDKK.KIATEG...............
BnLEA21 PSLLEKLHRNDSSSSSSS...EEEGEDGEKRKKKKDKK.KIATEG...............
BnLEA22 HSLLEKLHRSDSSSSSSS...EEEGEDGVKRKKKKDKKK.VTATEG..............
BnLEA67 PSLLEKLHRSDSSSSSSS...EEEGEDGEKRKKKKDKK.KIATEG...............
BnLEA68 G...EHLRRSGSSSSSSS...EDDGQGGRRKKG...........................
BnLEA69 G.LSGMLHRSGSSSSSSSSS.EDDGLGGRRKKK...........................
BnLEA70 G...EHLRRSGSSSSSSS...EDDGQGGRRKKG...........................
BnLEA71 G.LSGMLHRSGSSSSSSSSS.EDDGLGGRRKK............................
BnLEA88 GVTEKIMLKVHAGKGTHEQANKHEHEDGEKKGFMEKMKEKLPAAGG..............
BnLEA89 ....NPLENMGISSSNSM...DEYGQGSRQGAN...........................
BnLEA90 ....NPLENKGISSSNSM...DEYGQGSRQGAT...........................
BnLEA104 RGLGGMLHRSGSGSSSSS...EDDGQGGRRKKK...........................
BnLEA105 G.LGGMLHRSGSGSSSSS...EDDGQGGRRKKK...........................
BnLEA106 ..LGGMLHRSGSGSSSSS...EDDGQGGRRKKK...........................
BnLEA107 G.LGGMLHRSGSGSSSSS...EDDGQGGRRKKK...........................
BnLEA108 G.LGGMLHRSGSGSSSSS...EDDGQGGRRKKK...........................
consensus>70 ..l...lhrs.s.sssss...eeege.g...kk...........................

```
                                                 140       150       160       170
                                                   .         .         .         .
BnLEA7         ...........................EEKKGVMEKIKEKLPGHSEK..PDDSQVVNTEA
BnLEA8         ......................EVKTEEEKKGFMDKLKEKLPGHGKK..PED...ASPAA
BnLEA9         ...........................DEKKGVMEKIKEKLPGHSEK..PDDSQVVNTEA
BnLEA10        FPHGTKTEDDTPVIATLPVKEETVEHPEEKKRLMEKIKEKLPGHSEK..PEDSQVVDTAA
BnLEA15        ................................IVDKIKDKIHGGEGH..SSGDHKHDGEK
BnLEA18        ......................EVKTEEEKKGFMDKLKEKLPGHGKK..PED...ASPAA
BnLEA19        ......................EVKTEEEKKGFMDKLKEKLPGHGKK..PEE....PSPA
BnLEA20        ......................EVQTEEAKKGFMDKLKEKLPGHGKK..PEDDS.AVAAA
BnLEA21        ......................EVQTEEEKKGFMDKLKEKLPGHGKK..PEDDS.TAVAA
BnLEA22        ......................EVKTEEEKKGFMDKLKEKLPGHGKK..PEEKPEDASPA
BnLEA67        ......................EVQTEEAKKGFMDKLKEKLPGHGKK..PEDDS.AVAAA
BnLEA68        ................................MKDKIKEKLSGGKHKDEQTPSTATTTGP
BnLEA69        ...............................GITAKIKEKLPGHHGSSHQTSS..ATSTI
BnLEA70        ................................IKDKIKEKLSSDKHKDEQTPSTATTTGP
BnLEA71        ...............................GITAKIKEKLPGHHG.SHQTSS..ATSTI
BnLEA88        ..............HHDQANKPEHQEDGKEKGFMEKIKGKLPAPGGHHDQANKHEHHEDG
BnLEA89        ................................VTDETKSKVEG......HDPSTATVSG.
BnLEA90        ................................VTDETKSKVEG......HDRSTATVSG.
BnLEA104       ...............................GITDKIKEKLPGHHDQSSGQSQGMGMGTT
BnLEA105       ...............................GITDKIKEKLPGHHDQS.GQSQGMGMGTT
BnLEA106       ...............................GITDKIKEKLPGHHDQS.GQSQGMGMGTT
BnLEA107       ...............................GITDKIKEKLPGHHDQS.GQSQGMGMGTT
BnLEA108       ...............................GITDKIKEKLPGHHDQS.GQSQGMGMGTT
consensus>70   ...............................g..dk.KeKlpgh......e.........

                                             180       190       200       210
                                               .         .         .         .
BnLEA7         A..............VPVSDETAEHAE...KKGILEKIKEKLPGYHAKSSEEEEKK.EK
BnLEA8         P..............VVAPPVEEAHPAE...KKGILEKIKEKLPGYHPKTVDEVKKEKET
BnLEA9         A..............VPVSDETAEHPEE...KKGILEKIKEKLPGYHAKSSEEDEKK.EK
BnLEA10        A..............VPVTEKTAEHPEE...KKGLMGKIKEKLPGYHAKSTEEEEKKKEK
BnLEA15        K................KKKDKKEKKHH...DDGHHSSSSDSDSD...............
BnLEA18        P..............VVAPPVEEAHPAE...KKGILEKIKEKLPGYHPKTVDEVKKEKET
BnLEA19        P..............VVAPPVEEAHPAE...KKGILEKIKEKLPGYHPKTVEEEKKDKDD
BnLEA20        P..............VVAPPVEEAHPAE...KKGILEKIKEKLPGYHSKTVEEEKKDDH.
BnLEA21        P..............VVAPPVEEAHPAE...KKGILEKIKEKLP.........VPLKDR..
BnLEA22        P..............VVAPPVEEAHPAE...KKGILEKIKEKLPGYHSKTVEEEKKDKDD
BnLEA67        P..............VVAPPVEEAHPAE...KKGILEKIKEKLPGYHSKTVEEEKKDDH.
BnLEA68        T..............TTTGAAAADQHHE...KKGILEKIKEKLPGHHNHHP.........
BnLEA69        P..............VYDATGTGAVHHE...KKGIMEKIKEKLPGG..HH..........
BnLEA70        T..............TTTGAAATDQHHE...KKGILEKIKEKLPGHHNHHHP........
BnLEA71        P..............VYDATGTGAVHHE...KKGIMEKIKEKLPGG..HH..........
BnLEA88        KEKGFMDKIKEKIPGVHNGKPEVEPRHENGKEKGFMEKIKEKLPGHIKHDDSDEKKKET.
BnLEA89        ....................SGSEETHE...KKGFLKKIKEKISGNHNDP..........
BnLEA90        ....................SGSEEAHE...KKGFLNKIKEKLSGNHNDP..........
BnLEA104       T..............GYDAG...GERHE...KKGMMEKIKEKLPGGGGHH..........
BnLEA105       T..............GYDEGGYTGERHE...KKGMMEKIKEKLPGGG.HH..........
BnLEA106       T..............GYDAGGYGGERHE...KKGMMEKIKEKLPGGG.HH..........
BnLEA107       T..............GYDEGGYGGERHE...KKGMMEKIKEKLPGGG.HH..........
BnLEA108       T..............GYDEGGYTGERHE...KKGMMEKIKEKLPGGG.HH..........
consensus>70   ...........................e...kkG.lekik#klpg...............

BnLEA7         ESDA...
BnLEA8         D......
BnLEA9         VSDA...
BnLEA10        ESDDLEG
BnLEA15        .......
BnLEA18        D......
BnLEA19        H......
BnLEA20        .......
BnLEA21        .......
BnLEA22        H......
BnLEA67        .......
BnLEA68        .......
BnLEA69        .......
BnLEA70        .......
BnLEA71        .......
BnLEA88        .......
BnLEA89        .......
BnLEA90        .......
BnLEA104       .......
BnLEA105       .......
BnLEA106       .......
BnLEA107       .......
BnLEA108       .......
consensus>70   .......
```

BnLEA proteins of Dehydrin family

Supplementary Information

Figure S4

A

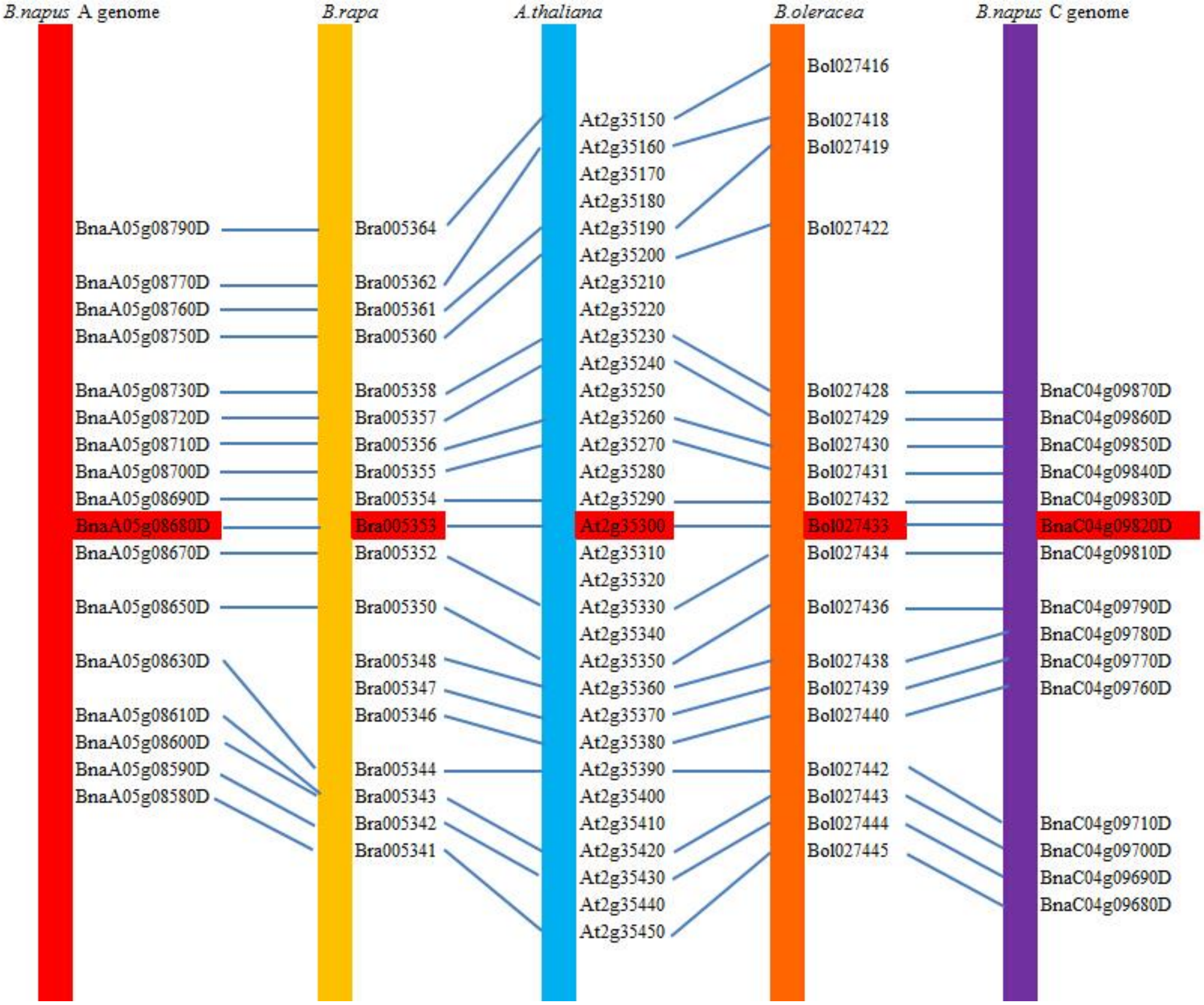

B.napus A genome
B.rapa
A.thaliana
B.oleracea
B.napus C genome
BnaA05g08790D
BnaA05g08770D
BnaA05g08760D
BnaA05g08750D
BnaA05g08730D
BnaA05g08720D
BnaA05g08710D
BnaA05g08700D
BnaA05g08690D
BnaA05g08680D
BnaA05g08670D
BnaA05g08650D
BnaA05g08630D
BnaA05g08610D
BnaA05g08600D
BnaA05g08590D
BnaA05g08580D
Bra005364
Bra005362
Bra005361
Bra005360
Bra005358
Bra005357
Bra005356
Bra005355
Bra005354
Bra005353
Bra005352
Bra005350
Bra005348
Bra005347
Bra005346
Bra005344
Bra005343
Bra005342
Bra005341
At2g35150
At2g35160
At2g35170
At2g35180
At2g35190
At2g35200
At2g35210
At2g35220
At2g35230
At2g35240
At2g35250
At2g35260
At2g35270
At2g35280
At2g35290
At2g35300
At2g35310
At2g35320
At2g35330
At2g35340
At2g35350
At2g35360
At2g35370
At2g35380
At2g35390
At2g35400
At2g35410
At2g35420
At2g35430
At2g35440
At2g35450
Bol027416
Bol027418
Bol027419
Bol027422
Bol027428
Bol027429
Bol027430
Bol027431
Bol027432
Bol027433
Bol027434
Bol027436
Bol027438
Bol027439
Bol027440
Bol027442
Bol027443
Bol027444
Bol027445
BnaC04g09870D
BnaC04g09860D
BnaC04g09850D
BnaC04g09840D
BnaC04g09830D
BnaC04g09820D
BnaC04g09810D
BnaC04g09790D
BnaC04g09780D
BnaC04g09770D
BnaC04g09760D
BnaC04g09710D
BnaC04g09700D
BnaC04g09690D
BnaC04g09680D

B

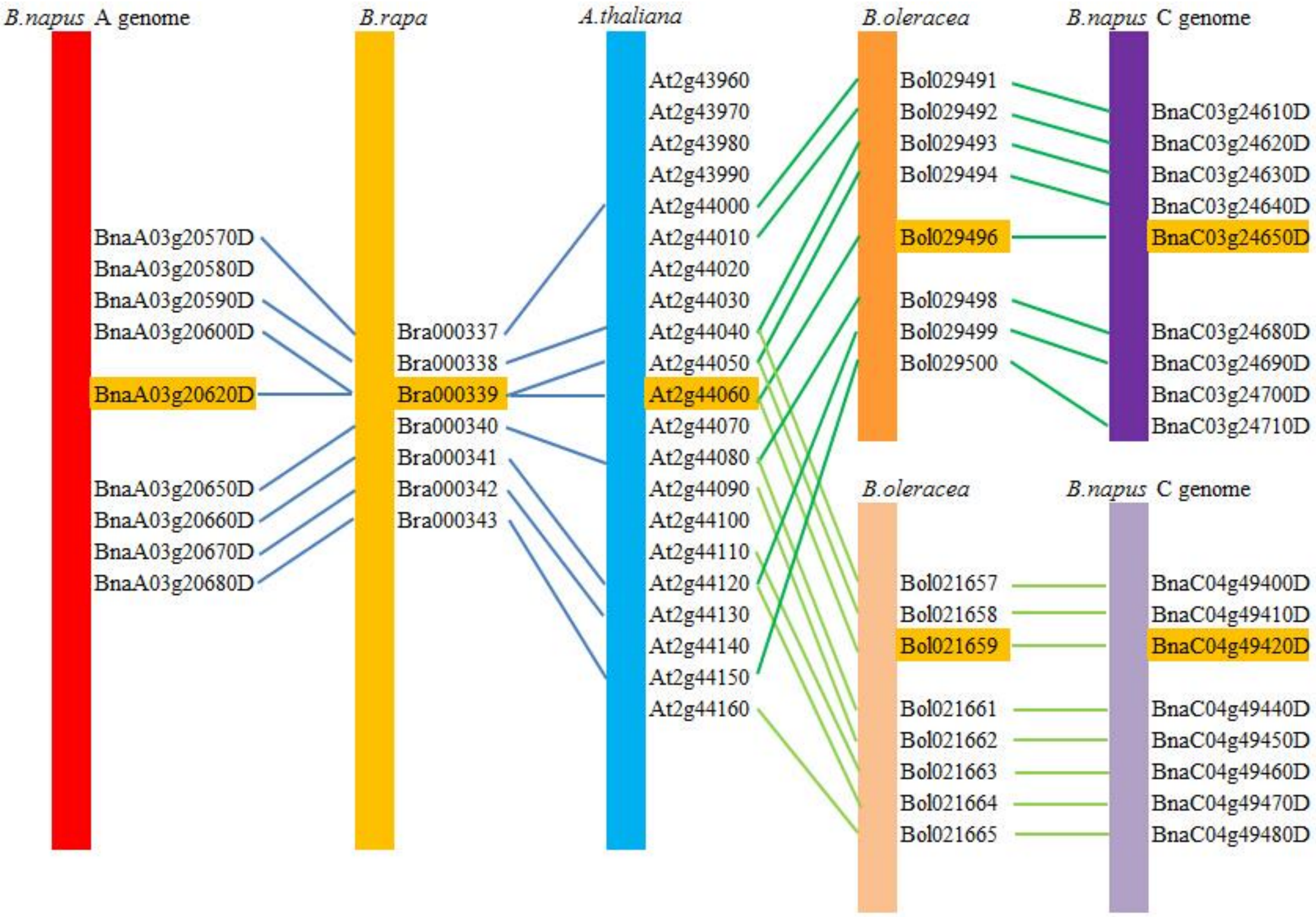

B.napus A genome
B.rapa
A.thaliana
B.oleracea
B.napus C genome
BnaA03g20570D
BnaA03g20580D
BnaA03g20590D
BnaA03g20600D
BnaA03g20620D
BnaA03g20650D
BnaA03g20660D
BnaA03g20670D
BnaA03g20680D
Bra000337
Bra000338
Bra000339
Bra000340
Bra000341
Bra000342
Bra000343
At2g43960
At2g43970
At2g43980
At2g43990
At2g44000
At2g44010
At2g44020
At2g44030
At2g44040
At2g44050
At2g44060
At2g44070
At2g44080
At2g44090
At2g44100
At2g44110
At2g44120
At2g44130
At2g44140
At2g44150
At2g44160
Bol029491
Bol029492
Bol029493
Bol029494
Bol029496
Bol029498
Bol029499
Bol029500
BnaC03g24610D
BnaC03g24620D
BnaC03g24630D
BnaC03g24640D
BnaC03g24650D
BnaC03g24680D
BnaC03g24690D
BnaC03g24700D
BnaC03g24710D
B.oleracea
B.napus C genome
Bol021657
Bol021658
Bol021659
Bol021661
Bol021662
Bol021663
Bol021664
Bol021665
BnaC04g49400D
BnaC04g49410D
BnaC04g49420D
BnaC04g49440D
BnaC04g49450D
BnaC04g49460D
BnaC04g49470D
BnaC04g49480D

C

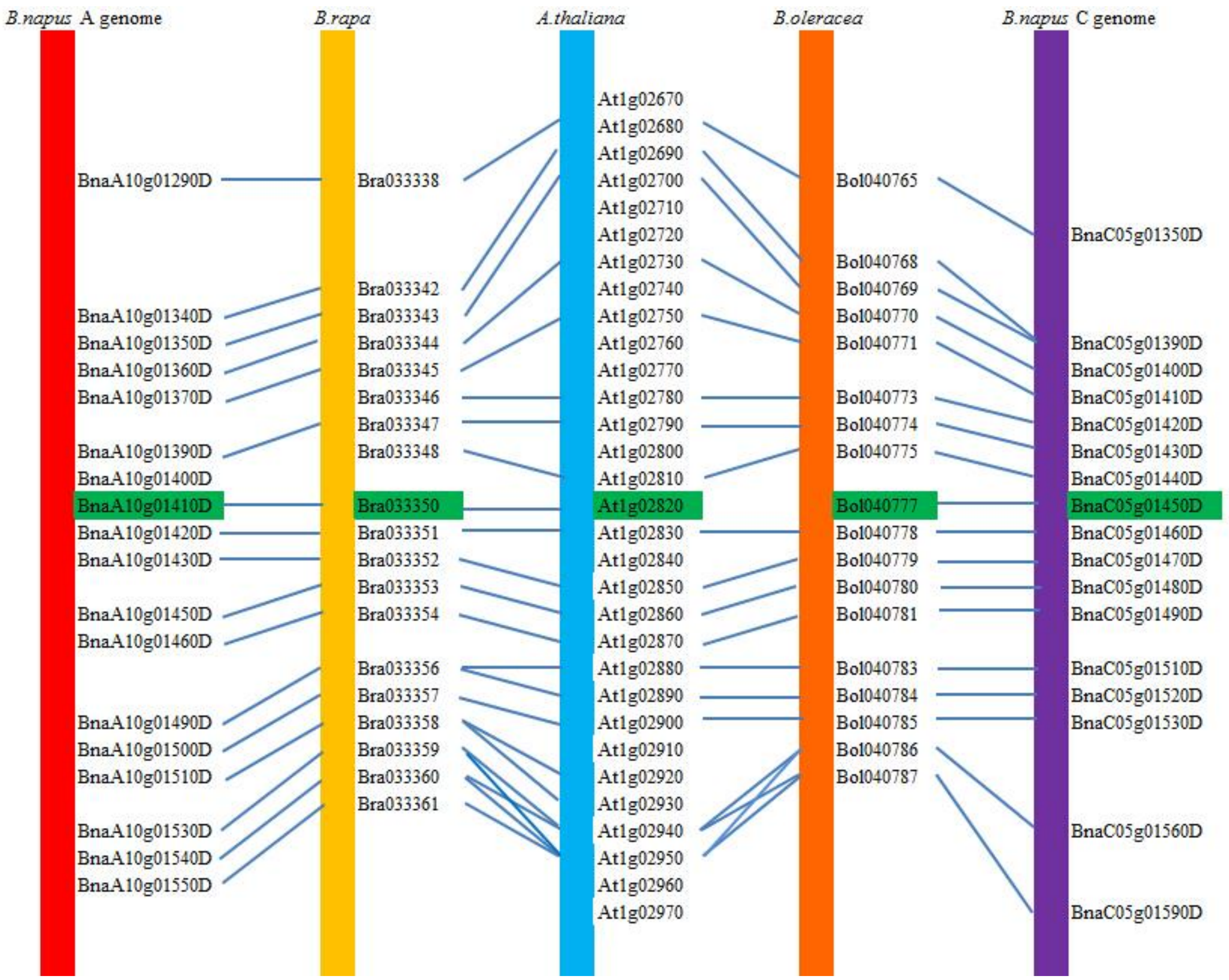
B.napus A genome
B.rapa
A.thaliana
B.oleracea
B.napus C genome
BnaA10g01290D
BnaA10g01340D
BnaA10g01350D
BnaA10g01360D
BnaA10g01370D
BnaA10g01390D
BnaA10g01400D
BnaA10g01410D
BnaA10g01420D
BnaA10g01430D
BnaA10g01450D
BnaA10g01460D
BnaA10g01490D
BnaA10g01500D
BnaA10g01510D
BnaA10g01530D
BnaA10g01540D
BnaA10g01550D
Bra033338
Bra033342
Bra033343
Bra033344
Bra033345
Bra033346
Bra033347
Bra033348
Bra033350
Bra033351
Bra033352
Bra033353
Bra033354
Bra033356
Bra033357
Bra033358
Bra033359
Bra033360
Bra033361
At1g02670
At1g02680
At1g02690
At1g02700
At1g02710
At1g02720
At1g02730
At1g02740
At1g02750
At1g02760
At1g02770
At1g02780
At1g02790
At1g02800
At1g02810
At1g02820
At1g02830
At1g02840
At1g02850
At1g02860
At1g02870
At1g02880
At1g02890
At1g02900
At1g02910
At1g02920
At1g02930
At1g02940
At1g02950
At1g02960
At1g02970
Bol040765
Bol040768
Bol040769
Bol040770
Bol040771
Bol040773
Bol040774
Bol040775
Bol040777
Bol040778
Bol040779
Bol040780
Bol040781
Bol040783
Bol040784
Bol040785
Bol040786
Bol040787
BnaC05g01350D
BnaC05g01390D
BnaC05g01400D
BnaC05g01410D
BnaC05g01420D
BnaC05g01430D
BnaC05g01440D
BnaC05g01450D
BnaC05g01460D
BnaC05g01470D
BnaC05g01480D
BnaC05g01490D
BnaC05g01510D
BnaC05g01520D
BnaC05g01530D
BnaC05g01560D
BnaC05g01590D

D

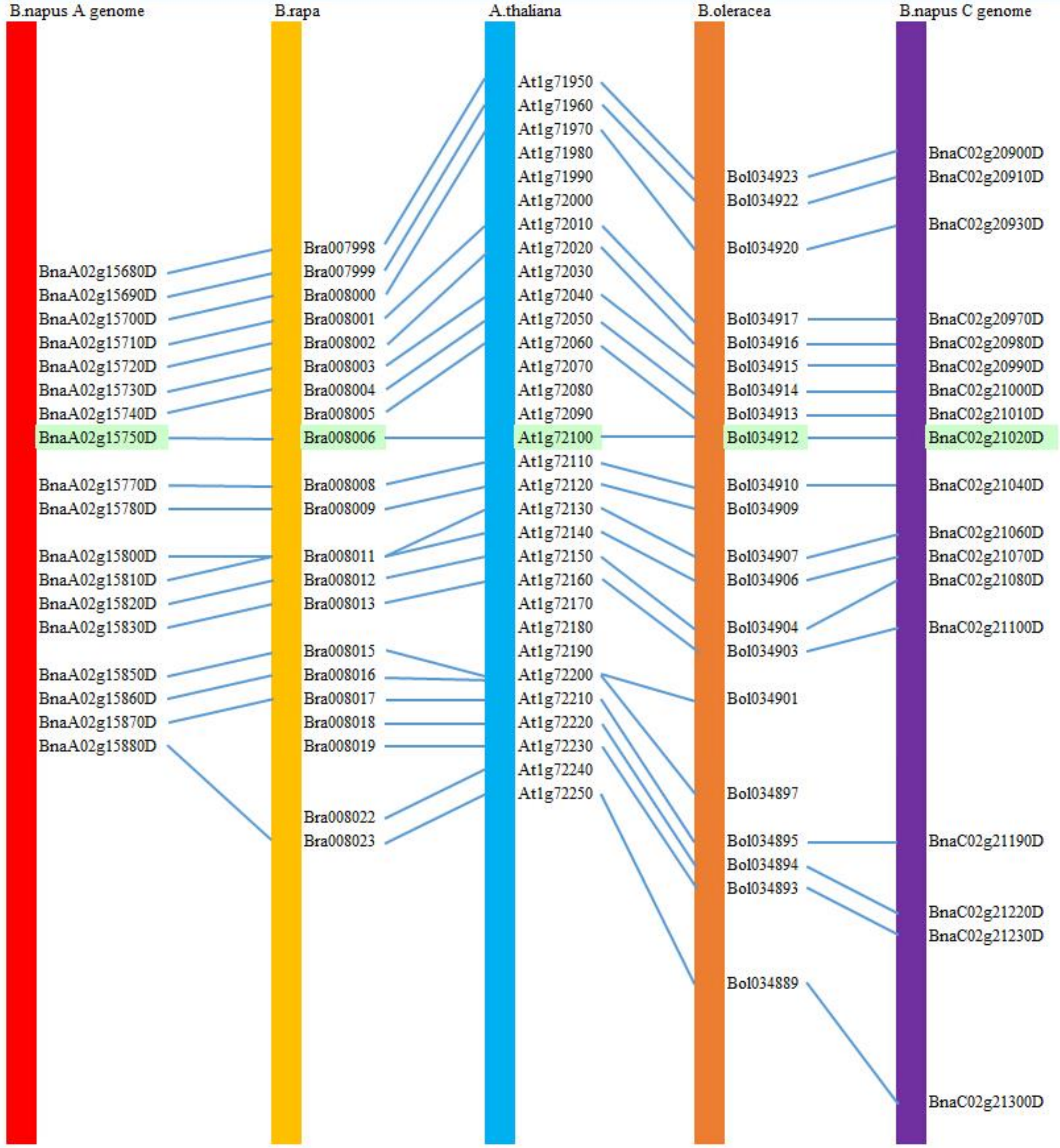
B.napus A genome
B.rapa
A.thaliana
B.oleracea
B.napus C genome
BnaA02g15680D
BnaA02g15690D
BnaA02g15700D
BnaA02g15710D
BnaA02g15720D
BnaA02g15730D
BnaA02g15740D
BnaA02g15750D
BnaA02g15770D
BnaA02g15780D
BnaA02g15800D
BnaA02g15810D
BnaA02g15820D
BnaA02g15830D
BnaA02g15850D
BnaA02g15860D
BnaA02g15870D
BnaA02g15880D
Bra007998
Bra007999
Bra008000
Bra008001
Bra008002
Bra008003
Bra008004
Bra008005
Bra008006
Bra008008
Bra008009
Bra008011
Bra008012
Bra008013
Bra008015
Bra008016
Bra008017
Bra008018
Bra008019
Bra008022
Bra008023
At1g71950
At1g71960
At1g71970
At1g71980
At1g71990
At1g72000
At1g72010
At1g72020
At1g72030
At1g72040
At1g72050
At1g72060
At1g72070
At1g72080
At1g72090
At1g72100
At1g72110
At1g72120
At1g72130
At1g72140
At1g72150
At1g72160
At1g72170
At1g72180
At1g72190
At1g72200
At1g72210
At1g72220
At1g72230
At1g72240
At1g72250
Bol034923
Bol034922
Bol034920
Bol034917
Bol034916
Bol034915
Bol034914
Bol034913
Bol034912
Bol034910
Bol034909
Bol034907
Bol034906
Bol034904
Bol034903
Bol034901
Bol034897
Bol034895
Bol034894
Bol034893
Bol034889
BnaC02g20900D
BnaC02g20910D
BnaC02g20930D
BnaC02g20970D
BnaC02g20980D
BnaC02g20990D
BnaC02g21000D
BnaC02g21010D
BnaC02g21020D
BnaC02g21040D
BnaC02g21060D
BnaC02g21070D
BnaC02g21080D
BnaC02g21100D
BnaC02g21190D
BnaC02g21220D
BnaC02g21230D
BnaC02g21300D

E

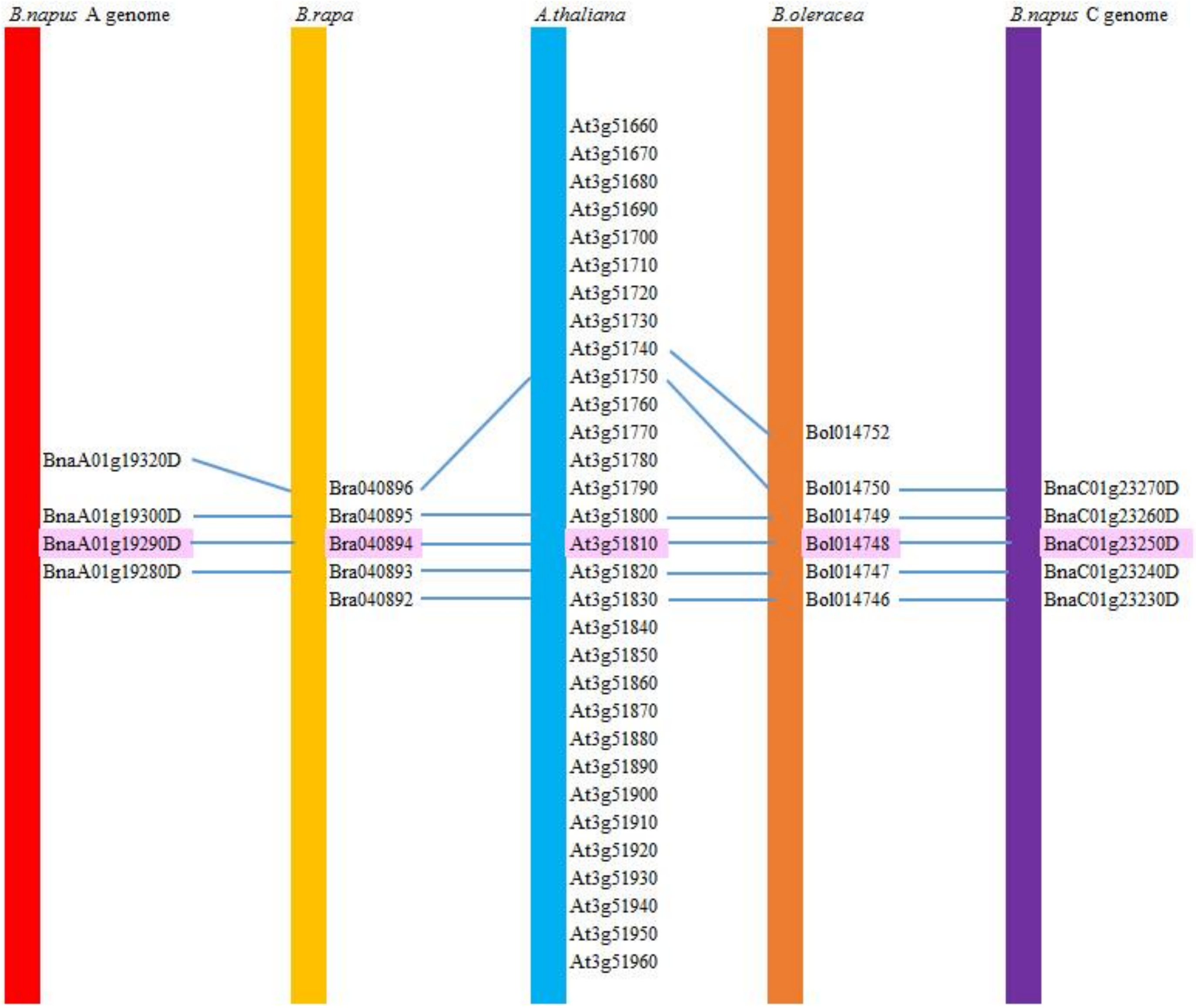

B.napus A genome
B.rapa
A.thaliana
B.oleracea
B.napus C genome
At3g51660
At3g51670
At3g51680
At3g51690
At3g51700
At3g51710
At3g51720
At3g51730
At3g51740
At3g51750
At3g51760
At3g51770
At3g51780
At3g51790
At3g51800
At3g51810
At3g51820
At3g51830
At3g51840
At3g51850
At3g51860
At3g51870
At3g51880
At3g51890
At3g51900
At3g51910
At3g51920
At3g51930
At3g51940
At3g51950
At3g51960
Bol014752
BnaA01g19320D
Bra040896
Bol014750
BnaC01g23270D
BnaA01g19300D
Bra040895
Bol014749
BnaC01g23260D
BnaA01g19290D
Bra040894
Bol014748
BnaC01g23250D
BnaA01g19280D
Bra040893
Bol014747
BnaC01g23240D
Bra040892
Bol014746
BnaC01g23230D

F

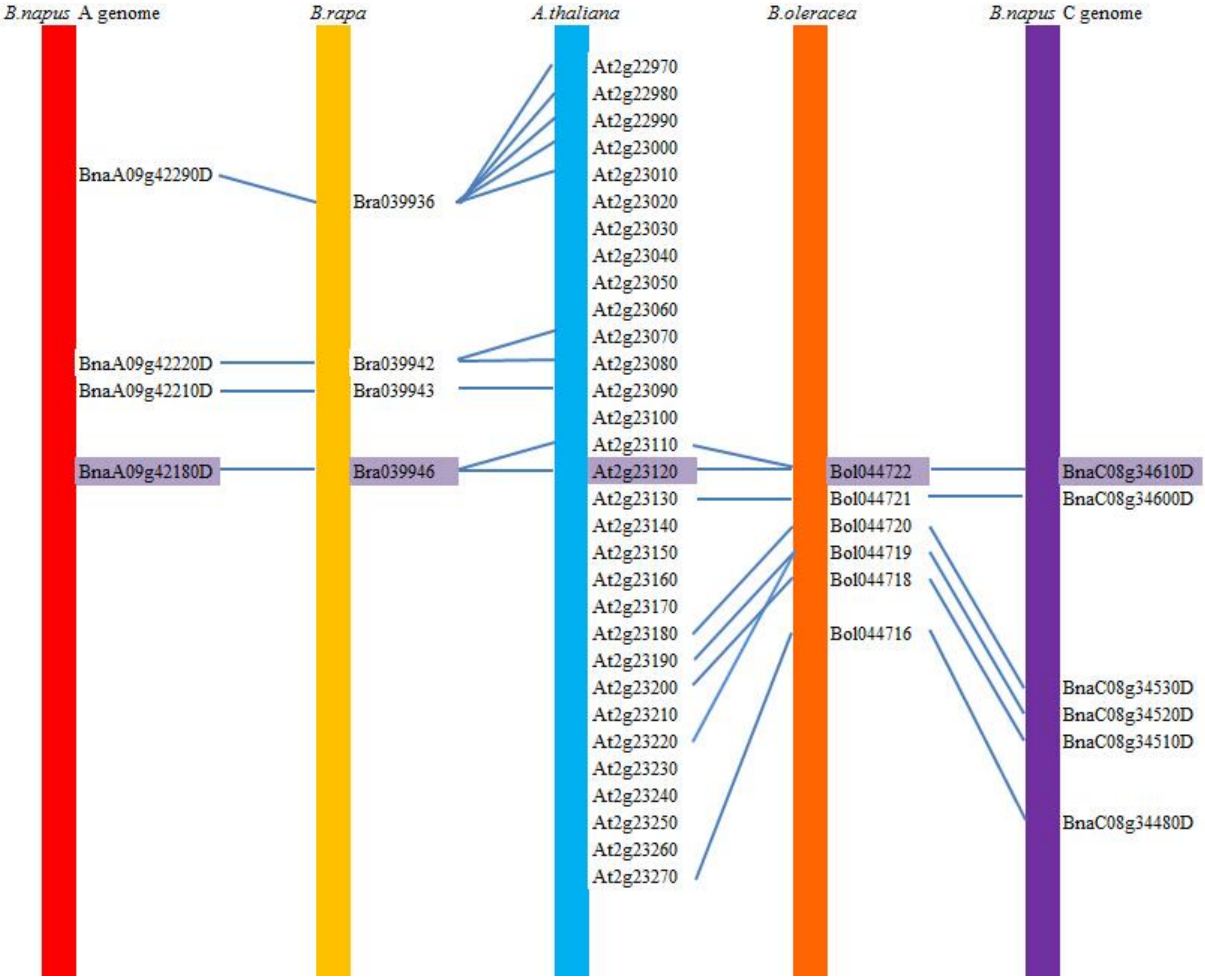
B.napus A genome
B.rapa
A.thaliana
B.oleracea
B.napus C genome
BnaA09g42290D
BnaA09g42220D
BnaA09g42210D
BnaA09g42180D
Bra039936
Bra039942
Bra039943
Bra039946
At2g22970
At2g22980
At2g22990
At2g23000
At2g23010
At2g23020
At2g23030
At2g23040
At2g23050
At2g23060
At2g23070
At2g23080
At2g23090
At2g23100
At2g23110
At2g23120
At2g23130
At2g23140
At2g23150
At2g23160
At2g23170
At2g23180
At2g23190
At2g23200
At2g23210
At2g23220
At2g23230
At2g23240
At2g23250
At2g23260
At2g23270
Bol044722
Bol044721
Bol044720
Bol044719
Bol044718
Bol044716
BnaC08g34610D
BnaC08g34600D
BnaC08g34530D
BnaC08g34520D
BnaC08g34510D
BnaC08g34480D

G

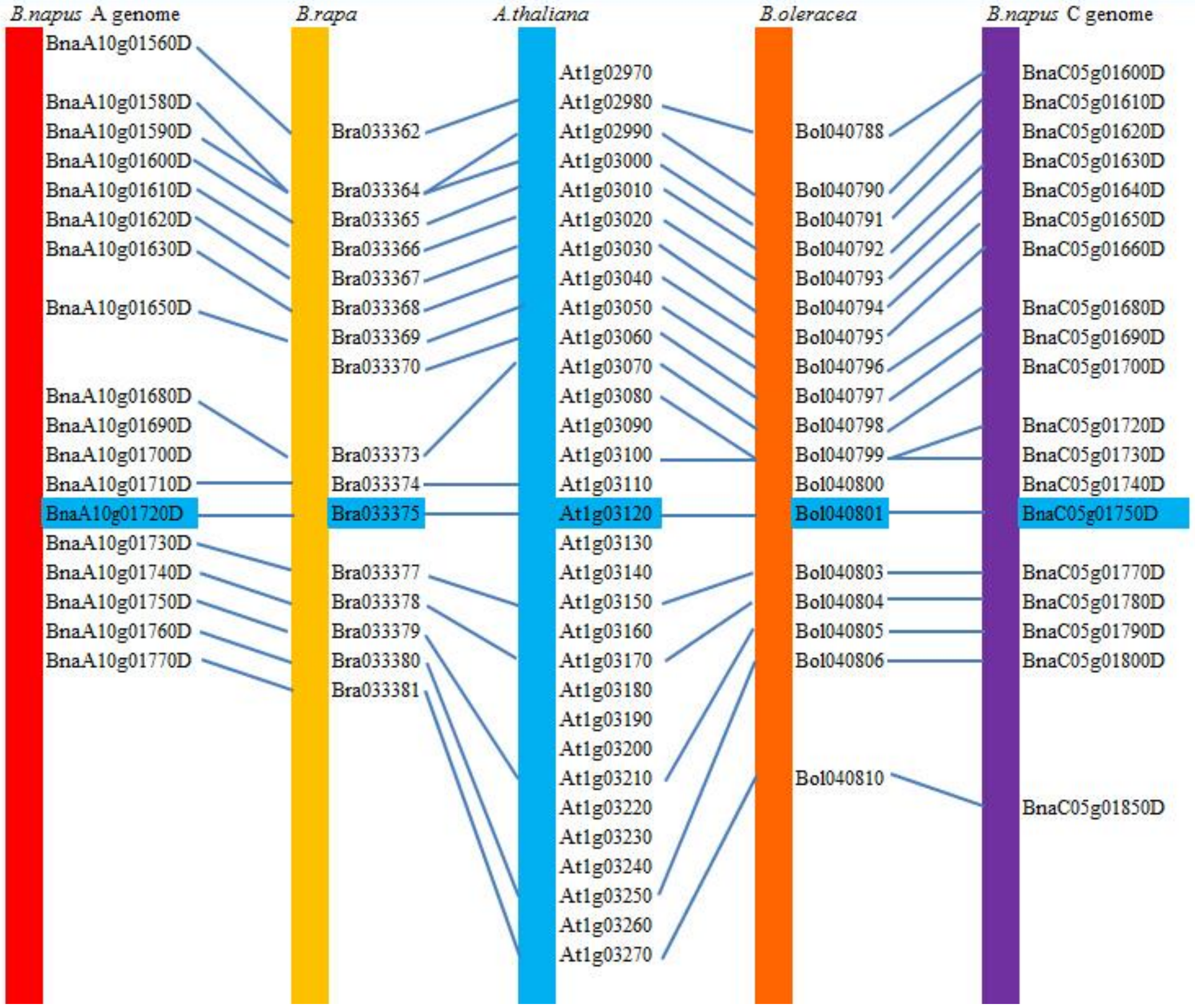
B.napus A genome
B.rapa
A.thaliana
B.oleracea
B.napus C genome
BnaA10g01560D
BnaA10g01580D
BnaA10g01590D
BnaA10g01600D
BnaA10g01610D
BnaA10g01620D
BnaA10g01630D
BnaA10g01650D
BnaA10g01680D
BnaA10g01690D
BnaA10g01700D
BnaA10g01710D
BnaA10g01720D
BnaA10g01730D
BnaA10g01740D
BnaA10g01750D
BnaA10g01760D
BnaA10g01770D
Bra033362
Bra033364
Bra033365
Bra033366
Bra033367
Bra033368
Bra033369
Bra033370
Bra033373
Bra033374
Bra033375
Bra033377
Bra033378
Bra033379
Bra033380
Bra033381
At1g02970
At1g02980
At1g02990
At1g03000
At1g03010
At1g03020
At1g03030
At1g03040
At1g03050
At1g03060
At1g03070
At1g03080
At1g03090
At1g03100
At1g03110
At1g03120
At1g03130
At1g03140
At1g03150
At1g03160
At1g03170
At1g03180
At1g03190
At1g03200
At1g03210
At1g03220
At1g03230
At1g03240
At1g03250
At1g03260
At1g03270
Bol040788
Bol040790
Bol040791
Bol040792
Bol040793
Bol040794
Bol040795
Bol040796
Bol040797
Bol040798
Bol040799
Bol040800
Bol040801
Bol040803
Bol040804
Bol040805
Bol040806
Bol040810
BnaC05g01600D
BnaC05g01610D
BnaC05g01620D
BnaC05g01630D
BnaC05g01640D
BnaC05g01650D
BnaC05g01660D
BnaC05g01680D
BnaC05g01690D
BnaC05g01700D
BnaC05g01720D
BnaC05g01730D
BnaC05g01740D
BnaC05g01750D
BnaC05g01770D
BnaC05g01780D
BnaC05g01790D
BnaC05g01800D
BnaC05g01850D

H

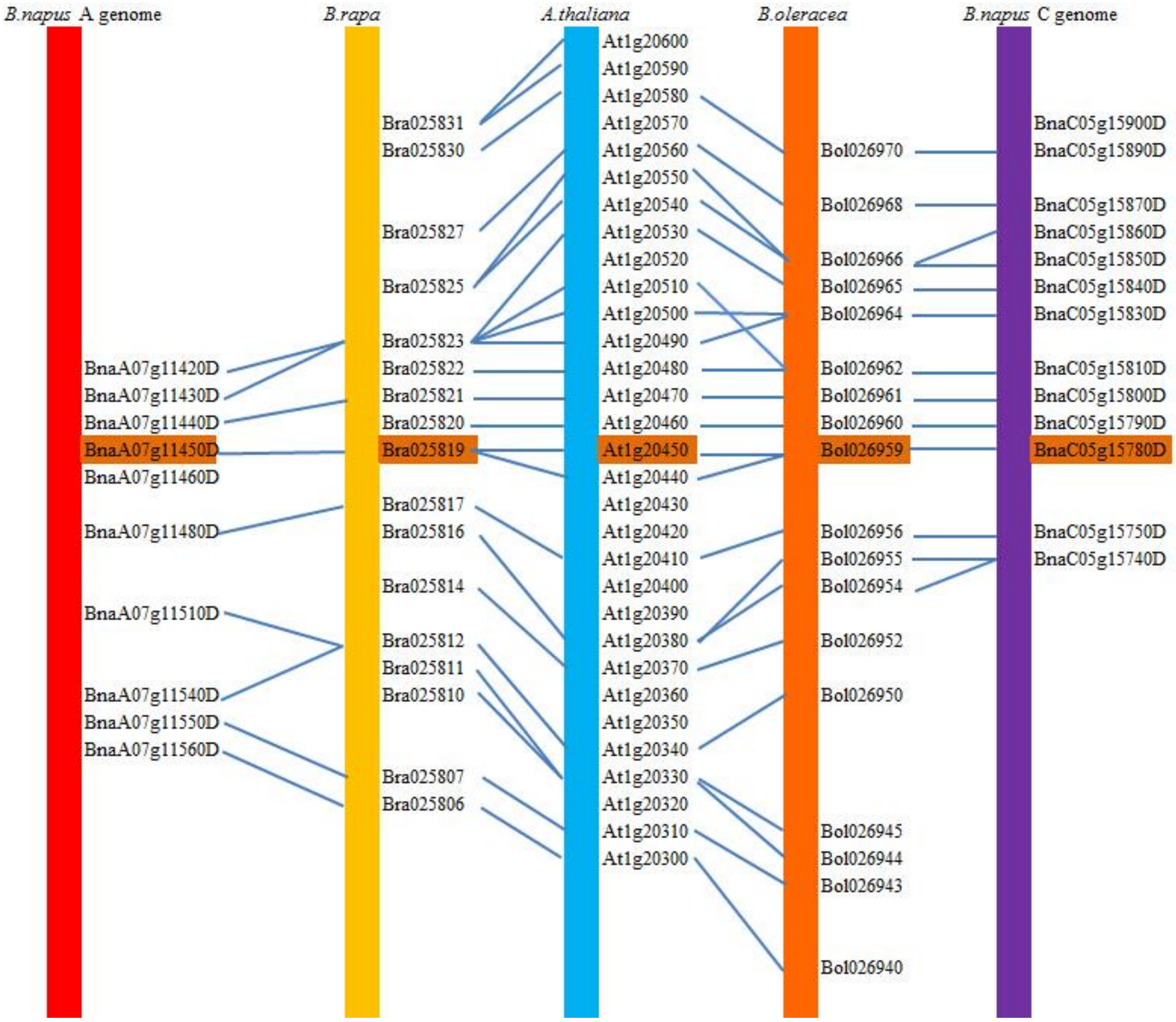
B.napus A genome
B.rapa
A.thaliana
B.oleracea
B.napus C genome
BnaA07g11420D
BnaA07g11430D
BnaA07g11440D
BnaA07g11450D
BnaA07g11460D
BnaA07g11480D
BnaA07g11510D
BnaA07g11540D
BnaA07g11550D
BnaA07g11560D
Bra025831
Bra025830
Bra025827
Bra025825
Bra025823
Bra025822
Bra025821
Bra025820
Bra025819
Bra025817
Bra025816
Bra025814
Bra025812
Bra025811
Bra025810
Bra025807
Bra025806
At1g20600
At1g20590
At1g20580
At1g20570
At1g20560
At1g20550
At1g20540
At1g20530
At1g20520
At1g20510
At1g20500
At1g20490
At1g20480
At1g20470
At1g20460
At1g20450
At1g20440
At1g20430
At1g20420
At1g20410
At1g20400
At1g20390
At1g20380
At1g20370
At1g20360
At1g20350
At1g20340
At1g20330
At1g20320
At1g20310
At1g20300
Bol026970
Bol026968
Bol026966
Bol026965
Bol026964
Bol026962
Bol026961
Bol026960
Bol026959
Bol026956
Bol026955
Bol026954
Bol026952
Bol026950
Bol026945
Bol026944
Bol026943
Bol026940
BnaC05g15900D
BnaC05g15890D
BnaC05g15870D
BnaC05g15860D
BnaC05g15850D
BnaC05g15840D
BnaC05g15830D
BnaC05g15810D
BnaC05g15800D
BnaC05g15790D
BnaC05g15780D
BnaC05g15750D
BnaC05g15740D

Supplementary Information

Table S1. Datas of subcellular location predition.

Gene	Subcellular location PProwler				TargetP			
	SP	MTP	CTP	other	cTP	mTP	SP	other
BnLEA1	0.07	0.17	0.03	0.73	0.16	0.174	0.174	0.351
BnLEA2	0.08	0.17	0.03	0.73	0.16	0.174	0.174	0.351
BnLEA3	0.76	0.14	0.07	0.03	0.063	0.037	0.635	0.193
BnLEA4	0.84	0.08	0.05	0.04	0.067	0.035	0.676	0.163
BnLEA5	0.82	0.07	0.01	0.08	0.115	0.04	0.804	0.034
BnLEA6	0.83	0.08	0.02	0.08	0.102	0.037	0.829	0.035
BnLEA7	0.52	0.23	0.09	0.16	0.108	0.115	0.073	0.354
BnLEA8	0.04	0.14	0.01	0.81	0.13	0.144	0.114	0.445
BnLEA9	0.54	0.23	0.09	0.15	0.088	0.109	0.074	0.404
BnLEA10	0.38	0.23	0.07	0.32	0.078	0.181	0.066	0.411
BnLEA11	0.1	0.21	0.02	0.67	0.184	0.102	0.358	0.225
BnLEA12	0.12	0.22	0.03	0.64	0.173	0.131	0.367	0.197
BnLEA13	0.12	0.22	0.03	0.63	0.173	0.131	0.367	0.197
BnLEA14	0.71	0.17	0.08	0.05	0.067	0.027	0.322	0.29
BnLEA15	0.45	0.18	0.03	0.34	0.067	0.077	0.51	0.297
BnLEA16	0.2	0.2	0.06	0.53	0.427	0.206	0.019	0.426
BnLEA17	0.26	0.21	0.07	0.46	0.441	0.226	0.016	0.417
BnLEA18	0.08	0.21	0.02	0.69	0.144	0.114	0.273	0.22
BnLEA19	0.42	0.17	0.02	0.38	0.094	0.11	0.454	0.111
BnLEA20	0.05	0.15	0.01	0.8	0.132	0.179	0.17	0.293
BnLEA21	0.05	0.18	0.01	0.75	0.098	0.175	0.206	0.352
BnLEA22	0.52	0.15	0.02	0.3	0.101	0.091	0.597	0.06
BnLEA23	0.03	0.11	0.01	0.85	0.263	0.131	0.065	0.638
BnLEA24	0.02	0.09	0	0.89	0.207	0.178	0.029	0.698
BnLEA25	0.05	0.22	0.04	0.69	0.254	0.216	0.087	0.321
BnLEA26	0.03	0.11	0.01	0.85	0.231	0.121	0.197	0.35
BnLEA27	0.3	0.17	0.03	0.5	0.037	0.069	0.28	0.371
BnLEA28	0.33	0.19	0.04	0.44	0.028	0.044	0.418	0.294
BnLEA29	0.81	0.11	0.06	0.02	0.058	0.015	0.866	0.047
BnLEA30	0.8	0.12	0.04	0.04	0.029	0.021	0.842	0.073
BnLEA31	0.81	0.11	0.06	0.02	0.029	0.022	0.818	0.084
BnLEA32	0.23	0.21	0.05	0.52	0.047	0.111	0.27	0.396
BnLEA33	0.17	0.21	0.04	0.58	0.056	0.118	0.321	0.383
BnLEA34	0.04	0.14	0.01	0.81	0.06	0.182	0.308	0.382
BnLEA35	0.5	0.25	0.06	0.2	0.015	0.157	0.508	0.129
BnLEA36	0.61	0.19	0.05	0.14	0.034	0.084	0.608	0.099
BnLEA37	0.04	0.13	0.01	0.82	0.065	0.156	0.351	0.368
BnLEA38	0.17	0.19	0.02	0.62	0.039	0.061	0.537	0.313
BnLEA39	0.56	0.15	0.02	0.27	0.031	0.045	0.859	0.104
BnLEA40	0.12	0.2	0.02	0.66	0.049	0.087	0.634	0.212
BnLEA41	0.12	0.22	0.02	0.64	0.041	0.08	0.593	0.226
BnLEA42	0.12	0.2	0.02	0.66	0.049	0.087	0.634	0.212
BnLEA43	0.14	0.18	0.05	0.63	0.312	0.082	0.099	0.449

BnLEA44	0.06	0.23	0.02	0.69	0.086	0.115	0.156	0.339
BnLEA45	0.05	0.22	0.02	0.7	0.097	0.153	0.134	0.337
BnLEA46	0.37	0.19	0.08	0.36	0.534	0.066	0.262	0.141
BnLEA47	0.16	0.19	0.06	0.59	0.155	0.091	0.108	0.398
BnLEA48	0.7	0.1	0.03	0.17	0.315	0.027	0.615	0.124
BnLEA49	0.07	0.25	0.03	0.66	0.26	0.144	0.111	0.177
BnLEA50	0.05	0.19	0.02	0.75	0.286	0.132	0.047	0.379
BnLEA51	0.07	0.26	0.03	0.63	0.246	0.15	0.101	0.186
BnLEA52	0.05	0.2	0.02	0.73	0.35	0.129	0.041	0.291
BnLEA53	0.06	0.25	0.03	0.67	0.245	0.147	0.088	0.193
BnLEA54	0.89	0.06	0.02	0.03	0.046	0.034	0.739	0.108
BnLEA55	0.75	0.12	0.03	0.1	0.07	0.061	0.645	0.131
BnLEA56	0.88	0.07	0.02	0.03	0.052	0.043	0.795	0.082
BnLEA57	0.94	0.03	0	0.02	0.046	0.032	0.91	0.05
BnLEA58	0.12	0.15	0.05	0.68	0.286	0.209	0.016	0.548
BnLEA59	0.12	0.16	0.05	0.67	0.291	0.189	0.019	0.522
BnLEA60	0.1	0.14	0.04	0.72	0.21	0.296	0.024	0.508
BnLEA61	0.1	0.14	0.04	0.72	0.21	0.296	0.024	0.508
BnLEA62	0.78	0.11	0.05	0.05	0.051	0.044	0.923	0.022
BnLEA63	0.48	0.21	0.05	0.26	0.078	0.058	0.771	0.069
BnLEA64	0.78	0.11	0.05	0.06	0.058	0.043	0.915	0.022
BnLEA65	0.78	0.08	0.01	0.14	0.106	0.037	0.888	0.037
BnLEA66	0.78	0.08	0.01	0.14	0.106	0.037	0.888	0.037
BnLEA67	0.03	0.3	0	0.67	0.076	0.559	0.062	0.632
BnLEA68	0.63	0.19	0.08	0.1	0.047	0.101	0.131	0.543
BnLEA69	0.74	0.12	0.07	0.07	0.027	0.053	0.555	0.251
BnLEA70	0.61	0.2	0.08	0.11	0.047	0.103	0.13	0.53
BnLEA71	0.68	0.14	0.07	0.11	0.051	0.063	0.382	0.282
BnLEA72	0.35	0.19	0.02	0.44	0.072	0.053	0.789	0.096
BnLEA73	0.31	0.18	0.02	0.5	0.074	0.061	0.726	0.128
BnLEA74	0.64	0.23	0.1	0.03	0.405	0.096	0.037	0.317
BnLEA75	0.61	0.24	0.11	0.05	0.388	0.096	0.042	0.316
BnLEA76	0.59	0.25	0.08	0.09	0.512	0.067	0.043	0.379
BnLEA77	0.7	0.17	0.08	0.04	0.404	0.05	0.083	0.258
BnLEA78	0.58	0.22	0.07	0.14	0.455	0.057	0.056	0.405
BnLEA79	0.57	0.26	0.09	0.09	0.427	0.077	0.036	0.44
BnLEA80	0.62	0.21	0.07	0.1	0.321	0.088	0.067	0.307
BnLEA81	0.53	0.23	0.07	0.18	0.465	0.069	0.038	0.476
BnLEA82	0.6	0.17	0.04	0.19	0.231	0.033	0.121	0.608
BnLEA83	0.43	0.18	0.03	0.36	0.34	0.053	0.044	0.615
BnLEA84	0.62	0.16	0.04	0.19	0.117	0.14	0.115	0.456
BnLEA85	0.68	0.19	0.09	0.04	0.046	0.072	0.525	0.062
BnLEA86	0.67	0.21	0.09	0.03	0.553	0.035	0.151	0.102
BnLEA87	0.71	0.19	0.08	0.02	0.388	0.025	0.303	0.065
BnLEA88	0.24	0.22	0.03	0.51	0.136	0.085	0.185	0.387
BnLEA89	0.64	0.16	0.04	0.16	0.056	0.112	0.237	0.381

BnLEA90	0.17	0.21	0.03	0.59	0.068	0.12	0.225	0.378
BnLEA91	0.55	0.22	0.08	0.16	0.24	0.128	0.14	0.176
BnLEA92	0.19	0.18	0.03	0.59	0.229	0.116	0.17	0.177
BnLEA93	0.24	0.21	0.04	0.51	0.238	0.119	0.117	0.25
BnLEA94	0.22	0.2	0.04	0.54	0.247	0.095	0.218	0.144
BnLEA95	0.42	0.17	0.03	0.37	0.13	0.161	0.262	0.273
BnLEA96	0.58	0.13	0.03	0.26	0.291	0.105	0.404	0.152
BnLEA97	0.32	0.23	0.04	0.4	0.172	0.106	0.447	0.09
BnLEA98	0.42	0.17	0.03	0.37	0.13	0.161	0.262	0.273
BnLEA99	0.22	0.14	0.01	0.63	0.206	0.175	0.119	0.431
BnLEA100	0.71	0.14	0.03	0.12	0.156	0.056	0.682	0.073
BnLEA101	0.24	0.25	0.07	0.43	0.309	0.112	0.054	0.313
BnLEA102	0.45	0.3	0.09	0.16	0.393	0.119	0.053	0.301
BnLEA103	0.27	0.26	0.08	0.4	0.289	0.11	0.056	0.323
BnLEA104	0.71	0.12	0.04	0.14	0.034	0.036	0.749	0.261
BnLEA105	0.76	0.12	0.07	0.05	0.014	0.029	0.843	0.217
BnLEA106	0.69	0.13	0.05	0.14	0.036	0.034	0.76	0.252
BnLEA107	0.79	0.09	0.05	0.07	0.019	0.034	0.841	0.164
BnLEA108	0.76	0.12	0.07	0.05	0.014	0.029	0.843	0.217

Supplementary Information

Table S2. Homology alignments data in different *BnLEA* gene families.

Family

	consensus positions	identify positions
LEA_1	60.50%	21.10%
LEA_2	47%	12.40%
LEA_3	65.10%	12.90%
LEA_4	17.50%	0.10%
LEA_5	57.80%	32%
LEA_6	79.20%	44.90%
SMP	57.30%	9.80%
Dehydrin	35.50%	2.40%

Table S3. Primer pairs used in quantitative RT-PCR

Gene name	Primer 1(5'--3')	Primer 2(5'--3')
BnLEA11	TGTACAGTGACCTTGTGCGA	TTCTACGAACATCCGACGAG
BnLEA32	ACCCGAACCGAAATTTAGAA	GTGGAAGTTCGGTACCCATT
BnLEA33	GAGACGACAGAACTTAGGT	GAGGTAAACGTCATCGA
BnLEA91	ACTATAGTAACACGTGGGT	CTTCATCGACTGCATTTT
BnLEA93	GTTAGAAGAAAATGCAGTCG	CAGACTTCGCAGAAGCTG
BnLEA1	AGAGACCTGAAATGTTTCT	GGAAATACGCGTAACGAA
BnLEA2	TAGTTAAAGGGCAAGTA	AATTGCACTATTAGACAC
BnLEA46	ACCTGACCTTTCACAACACGTT	TTGTCTAGGAAACCGCCTTC
BnLEA47	ACTTGAATGACTTCGAC	AACAGAGTCTGATATCTCTGCC
BnLEA48	GGTGCTATGAAGTTGCCTAT	ATACAACACAATGCTCAATC
BnLEA49	TGAACACCAAATGCAGTT	TTGAGACATTGACGAGTA
BnLEA50	CCTTGAGTGCTATTTTGTGA	ATTGCGGAGCGTGACATT
BnLEA51	TAAGTTCAGAGGCTGGA	ATTTGAGACTATGAACAT
BnLEA52	CGAATATGCATAATGGTGTT	CTTAATTGCCGGAGCGTGACA
BnLEA53	AGGAAGTAGGACATAAAT	GGTCCGAAGCATCATGGT
BnLEA3	TAGTTACAGATTACTATTAG	ATGAATGGCTGCGTGAC
BnLEA4	CAAGTTACAAATTATTATTAATT	GCTGCGTCAATCTAGGCTTCT
BnLEA74	CCGTGAGACGCACCATT	TTACACCTGGACAATGAC
BnLEA75	TCAATCCGTAAGACACAC	ACACCTCAATGACCTAT
BnLEA76	CGTGGTTATGCGGCTACGG	GGGATCAGGAATCCAAGAAA
BnLEA77	TACAACACCTAAGTCTAA	ACGGACATGAAGACTGTA
BnLEA78	AACCTCCCAAACAACAAG	ACGAGCCATGTTTATCG
BnLEA79	AAGACCGGTTCTGCCTAA	CTCCATGGGTAGGCTGAAG
BnLEA80	ATAGAGTCATCCTGCT	GGTACCAGGCTCAAATGAGCA
BnLEA81	AGTTTCAACCTTTTACTGTC	AGTTTCAACCTTTTACTGTC
BnLEA82	GTTCACTCTCCGGCGCCGTT	ATCTTCCCCACCGTAACTCTT
BnLEA83	CACTCTCCGCTGGGGTTAA	AAGAGTTACGGTGGGAAAGT
BnLEA84	CTCGTTCCATTGTCTTA	GAGCTTCTCCACGTAACTCTG
BnLEA14	GCTACATGTCGGAGACTGGA	TAAACCCACCGGTCTTATCC
BnLEA16	TCACTGATATTGGGTACATT	TTGCGGTTTTGCCACATC
BnLEA17	AGGTGAATCAATAGGC	TAGTACTCGTAATGGAAG
BnLEA23	ATCTCCGGAGCTGTGCTTAG	CATGACCCATGACTTCTTGC
BnLEA24	TTGATCAACGGAAGCAAGAG	CTAGGGCCCATGACTTGTTT
BnLEA25	CTTGGACTGGTTGGGTTTCT	ACAAAGTCCTTGGCGTATCC
BnLEA26	TGATAATGCGGGAAATTCAA	TATGCCATGTCCTTCACGTT
BnLEA34	TACAAGTAATCAGTTTACA	TAAAATTATTACTATGTGTA
BnLEA35	ATGCTTGGTTATGACTTA	CACGTGGTACATAGGAGCTGC
BnLEA36	GCCAATGACCATCATGCATGA	GTGGTAGATAGGCAGACG
BnLEA37	GGATATTGACACTTATATT	TTAAAATTACTATGTGTACC
BnLEA43	GTCGTTGATCTACGCCGATA	TCAACGCCTCATTTGTCTTC
BnLEA44	CAAAGGGAACGAAGGAGAAG	TCCCGTTCCTTCCTTATCAC
BnLEA45	TGATTACGTGACGGAGAAGG	TTGCCTCTGTCAGCTTGTTC
BnLEA54	TTTCTATATCATCGTCATCTG	ACCCAGACTGATGCACG
BnLEA55	CAACACGTGGGCCGTTGT	CCATTACAGCGAAAGACC
BnLEA56	GCTGCTAATTATACGTG	TGGATATATATTCAATCAGAATTG
BnLEA57	TGCGGATCGCGTTAAT	CATAACCAACCAAAATG

BnLEA58	TATCTATTATAAGCAAAG	AGGCGTGATATCGAAT
BnLEA59	GTAAGCGTTAGGTAAAAATGGG	CTTCATGCTTCAGACCGATC
BnLEA60	GGTAACATATGATATGTAACACGTGT	GAGATGGTTTGTCGACACATG
BnLEA61	CGAGTTCTTAATTATAAA	AGATATGAGTCCGTACGTTGC
BnLEA85	TCCCATTACCGTGTACATGT	GTTGAAGTGAACAGTTTGGC
BnLEA86	AGATTATGTTGGGATG	GATCAGAGCCGTGCCTA
BnLEA87	CCACACGTGTGGACATATT	GTGGTTATCAATAGCAT
BnLEA38	CAACGTGGAGACACTAGC	CGACTTGACGATGATGAATAA
BnLEA39	ATGAACACCAAAGATAAGCCTGG	CAGAAAGCTTTAGATTAGGAAT
BnLEA40	TACCGATTGTTCCTGA	ATGATGATACGAATACTCG
BnLEA41	CCGATCTCTTCGATTTTCTGT	ATAGCTCTTAAAATGTAGC
BnLEA42	TAGACCGGTCTCTTCGA	ACAGACTTGGCTTTAAG
BnLEA72	ACATAAATTGGATTAGGCTACT	AGTTGTATTTAACATGATTACC
BnLEA73	CTAGAAATTGGATTAAGTTACT	GGACTCGAAAGTGTTATG
BnLEA27	TCAACCGAGTAGCTGCCGCGTGT	CGGTGGTATCTTCTCTGCCTCCAT
BnLEA28	TTCAGGCCGCTTTATCTCTCGG	CGCGGTTGATTTTGATCTACGT
BnLEA29	TCACTTGTTTATTCTTTCTA	TCTCTGTTGGTGTCTTTGACAT
BnLEA30	CCATGCCTAGTATTGTGC	TAATATTCCGAACACG
BnLEA31	ACTATCTTTACTCTTTACACAAG	CGTACTTCGTGTACGGAC
BnLEA5	TAGTTTTAACTCTTTCACATTAA	GCAAACTGTAACGTTATCA
BnLEA6	CCCACAAGTATCTCTCTAAGCA	ATGCCCGAGATAACTCGAAC
BnLEA62	TCTCAAACACATTTTCAGCGT	GTTTATCCTCTTGTAACTTCC
BnLEA63	CAAGCTAGGGAGAGTTGTT	TGTCGTCCAGGGACGGACACGTT
BnLEA64	CTCATGAGAACAGCAACCAAAGT	TCATGAGTTTTGCCAAGTGT
BnLEA65	CAAGCTATGGCCTAGGGAG	TGTCGTTCCAAGAACGGAC
BnLEA66	TTGTCTTCATCATCTATGC	TACAAGTTACAACAGCTCGT
BnLEA95	GTCCGAACGAAGAAGACGGAAGCT	AATACTATCCGGTTTAGC
BnLEA96	GACGTTACGGCGGAGTAAAT	ACACTTGGTTCGGTTTAGCC
BnLEA97	CAATTATCATATGGGCCTT	ATTAGGTATGAGCCTGAACC
BnLEA98	GCGGCGATCTTCACGGTTT	CATATAAGTACATGTATAAGC
BnLEA99	TCACTGTCAGACAAACCCGCC	TGTCTCATGCCCACTTGGTT
BnLEA100	TGATTGTCCCTGGCCATT	TATTATACATACACGGACA
BnLEA101	TGCATGGAAGGTCACTGTCG	CTCGTTTAAATAAGCCT
BnLEA102	ATAGAAAGAGGCGATAGTGTAACC	GACAATGTCGCGGAGACGAATCT
BnLEA103	GGAAATGCACGTGCTAGT	CGAGAGTTGCATCGGTG
BnLEA7	AGGATGTACTTCGACCACGTA	AGATTGGTTACGTTTAGGCC
BnLEA9	CCGATCCAATAGCTCCTC	ACCATCTTCTTCTTCTTCTCCTT
BnLEA10	TCCACACGGAACAAAGACAG	TTCTCCTCCGGATGCTCTA
BnLEA15	CCACAGCAGCGGAGACC	GCTGCTGTGGTGACCA
BnLEA19	TAACTACTACTGAAGGAG	GGTGCCGGTGAAGGCTCT
BnLEA20	CCGTGTTTCGATTTTCTTGTTCTAA	TACCTTTGGCACCTCCTGC
BnLEA21	ACCGACCAAATCTTGATCTA	CGTTGCTACCTTTGGCACTTCAT
BnLEA22	ACTGCTACTGAAGGAGAGG	AAGCGTCTTCAGGCTT
BnLEA67	GGATCCAGCTCTATCTTGGC	ATTAACGATGACGACCACCA
BnLEA68	CACATACATCGACGGATCTAG	ACATAATAACGAGCTGGTTTG
BnLEA69	CATCGTTGATGAGCTTA	CCTAGCTCTTCGACTTGT
BnLEA70	GTATCGAACACCAGCAG	GTTGCTGCTCCGTGGTA
BnLEA71	GACGGCGTTTACACAGG	AATGTATACCGTAGACA

BnLEA88	GAAGGTGTTTGGTCACAAGG	GTGACGCCCTTCTTCTC
BnLEA89	CACTGCTTACTATTTGTA	TCACAAAATACATATGATCTC
BnLEA90	CATGTATACACTACACAGAGCGC	CCGCCATTCTAATTAGC
BnLEA104	GATACGGGACAGCTGGTG	GTATCCTCCACCTGCAGTC
BnLEA105	CCATAGGCCATAACGTA	TCCATCGATCCGTGTTA
BnLEA106	GGATAAGGGTTACGTGTCTAACAT	CGTACGTGTTCGTAACAGTCCA

Table S4 original data of qRT-PCR

Gene name	root	stem	leaf	flower	late seeds	early seeds
BnLEA1	1	2.541098	0	0	0.233583	0.039281
BnLEA2	0	0	0	0	1	0.017638
BnLEA3	0	0	0	0.148644	0	0
BnLEA4	1	0.667705	4.181051	1.49595	26.43316	0.015015
BnLEA5	0	1	0	1.899564	0.040679	0.016027
BnLEA6	1	0.805288	0.699471	0.10548	1.771727	0.163397
BnLEA7	1	0.735774	0.287537	0.236895	15.17326	0.04504
BnLEA9	1	2.547075	278.0441	3.322812	0.07232	0.199703
BnLEA10	1	1.344228	1.068047	0.172241	0.173199	0.041161
BnLEA11	1	110.9442	262.7602	1.895805	56.57732	3.356233
BnLEA14	1	0.045191	4.346247	0.003726	10.92877	0.006716
BnLEA15	1	0.195225	9.055104	0.145055	0.007573	0.012842
BnLEA16	1	0.969162	0.049164	0.997421	0.000513	0.995356
BnLEA17	1	2.332914	1.646034	1.024453	0.085465	0.002112
BnLEA19	1	6016.802	2595.645	31.13654	237.723	4.643462
BnLEA20	1	2.055903	0.556764	1.057532	0.178701	4.439177
BnLEA21	1	4.906597	1.049947	1.309568	0.641293	0.791175
BnLEA22	1	5.051798	5.981155	0.080373	0.122063	0.664013
BnLEA23	1	1.70153	4.099184	110835.3	3.671506	0.638316
BnLEA24	1	0.034321	0.049888	24.63423	0.000288	0.155792
BnLEA25	1	0.058345	0.198678	0.207012	521.1775	0.016204
BnLEA26	1	5.133577	44.98784	3.241733	0.982237	0.175698
BnLEA27	1	0.0001	0.0001	0.0001	0.212648	0.177159
BnLEA28	1	0.695077	0.778437	0.435158	2.582023	0.08727
BnLEA29	0	0	0	1	0.063641	0.015582
BnLEA30	1	0	0	0.105822	0.028698	0.012406
BnLEA31	0	1	0.068139	0.572239	0.001358	0.000113
BnLEA32	1	0.330679	1.775648	0.032753	0.02155	0.018191
BnLEA33	0	0	18.73236	0	1	0
BnLEA34	1	0.951818	7.404595	0.392604	265.7652	0.208255
BnLEA35	1	0	0	2.280157	0.288117	0.059159
BnLEA36	0	0	0	0	0	0.026583
BnLEA37	1	0.169338	0.966688	0.017887	250.0952	0.052042
BnLEA38	0	1	3.349947	0.384188	6.203597	0.028341
BnLEA39	0	1	0.88129	0.077932	0.181572	0.020287
BnLEA40	1	0	0	1.034464	0.067545	0.01007
BnLEA41	0	0	0	1	15.49914	0.00985
BnLEA42	1	0	0	3.381656	3.078882	0.007675
BnLEA43	1	4.168476	1147.974	13.12549	0.052241	0.022366
BnLEA44	1	9.773567	1.260936	0.03518	2470.527	1.324243
BnLEA45	1	5.29064	80.58621	1.657996	1952.236	3.505423
BnLEA46	1	0.001143	0.417273	0.97591	0	0.399359
BnLEA47	1	1.634202	0.816958	1.566147	0.86303	3.856773
BnLEA48	1	0.554866	0.815221	0.692552	0.02492	0.007367
BnLEA49	1	0.116582	1.037946	1.327683	0.664045	0.121729
BnLEA50	1	1.153702	3.560597	9.892049	0.253996	0.101155

BnLEA51	0	0	0	0	0	0.007811
BnLEA52	1	1.06107	8.794036	0.190089	0.382486	0.030312
BnLEA53	0	1	0	2.57561	0.310061	0.008847
BnLEA54	0	1	4.799426	0	0.082492	0.009989
BnLEA55	0	1	3.393019	0	0.040642	0.009008
BnLEA56	0	1	5.164953	0	0.058777	0.009103
BnLEA57	0	0	0	0	1	0.027494
BnLEA58	0	1	3.582447	0	0.157271	0.04569
BnLEA59	1	0.481871	4.560507	0.154985	0.123187	0.026178
BnLEA60	1	1.914201	0.073002	0.019802	0	0
BnLEA61	0	1	50.48073	1.474899	0.31884	0.051832
BnLEA62	1	9.487723	4.446258	4.936671	307.4763	0.474311
BnLEA63	1	0.258459	1.237096	0.582995	0.574114	0.052441
BnLEA64	1	8.084862	3.758511	0.412072	0.024147	0.0093
BnLEA65	1	0.768053	8.774093	0.111724	0.16252	0.00624
BnLEA66	1	0.333631	2.456282	0.664292	0.088346	0.024869
BnLEA67	1	20.56304	39.28774	1.541578	0.054599	0.011904
BnLEA68	1	0.7476	2.48177	1.092153	8.266982	0.201108
BnLEA69	1	22.89523	58.2357	1.238962	86.05143	1.701657
BnLEA70	1	16.35712	30.77326	1.298147	29.7844	0.000934
BnLEA71	0	1	0.845795	2.849186	0.016003	0.036261
BnLEA72	1	0	0	0.207813	0.025556	0.00294
BnLEA73	1	0	0	0	0.009106	0.002461
BnLEA74	1	2.004148	15.89673	6.028977	0.081077	0.233529
BnLEA75	1	0.217921	3.909333	4.040809	0.503321	0.82265
BnLEA76	1	44.12028	45.86194	7.387607	173.7975	0.428784
BnLEA77	1	1.300973	7.767284	0.679927	0.025447	0.016502
BnLEA78	1	69.36906	125.5024	10.0982	0.881269	0.003569
BnLEA79	1	7.609014	28.52784	24.22768	0.524057	0.124889
BnLEA80	1	9.028133	19.67562	13.69503	0.943077	0.022962
BnLEA81	1	20.46095	41.36716	2.559547	3.760859	0.024209
BnLEA82	1	0.085182	0.094267	2.616891	442.8661	0.176242
BnLEA83	0	0	0	0	1	0.12329
BnLEA84	1	0.372586	1.26197	0.207254	0.168052	0.040914
BnLEA85	0	1	4.33222	0.231087	0.033904	0.006081
BnLEA86	0	1	2.70491	0.084289	0.0184	0.003015
bnLEA87	0	1	2.582334	0	0.044789	0
BnLEA88	1	0.615991	2.051897	0.780017	0.305035	0.051331
BnLEA89	0	1	4.719979	1.772336	1.837245	0.059876
BnLEA90	1	1.354827	4.044779	1.432372	0.050908	0.010104
BnLEA91	0	0	2218.021	0	1	0.527197
BnLEA93	1	45.3287	2.942474	2.190184	12900.86	5.123171
BnLEA95	1	2.036396	11.6482	4.886874	0.027502	0.004433
BnLEA96	1	1.655928	25.14076	5.573581	0.247099	0.514967
BnLEA97	1	20.4452	29.14897	3.581191	0.114878	0.004079
BnLEA98	1	0.095459	6.205002	3.171006	0.023748	0.040814
BnLEA99	1	26.52606	42.74647	26.80348	9.77097	0.036897
BnLEA100	1	0.000119	0.00058	0.000468	0.000169	0

BnLEA101	1	0.053438	0.749324	0.117413	334.9102	0
BnLEA102	1	0.909144	0.488847	0.240814	24.08767	0.030616
BnLEA103	1	0	17.38267	19.14147	0.190782	0.02797
BnLEA104	1	1.307572	4.584392	1.539896	0.110077	0.022053
BnLEA105	1	1.133613	3.395185	1.036285	0.696729	0.055004
BnLEA106	1	0.404	0.041683	0.34391	0.926538	0.98483

Supplementary information

Figure legends

Figure S1. The phylogenetic relationship of the seventeen plant species.

Figure S2. Relatively high homology regions of *BnLEA* gene families. A: SMP, B: LEA_3, C: LEA_6, D: LEA_2, E: LEA_5, F: LEA_1, G: LEA_4, H: dehydrin.

Figure S3. Alignment of BnLEA protein sequences in each families.

Figure S4. Synteny analysis of genes of each family between *A. thaliana*, *B. rapa*, *B. oleracea* and *B. napus*.
A: LEA_1, B: LEA_2, C: LEA_3, D: LEA_4, E: LEA_5, F: LEA_6, G: SMP, H: Dehydrin.
